# Supplementary material for: Dehydration of a crystal hydrate at subglacial temperatures
Source: Nature. 2023 Apr 12;616(7956):288–92. doi: 10.1038/s41586-023-05749-7 (PMC10097597; doi:10.1038/s41586-023-05749-7)
Supplement: Supplementary file 1 — This file contains Supplementary Text, Figs. 1–71, Tables 1–17, captions for Videos 1–11 and references. [file 41586_2023_5749_MOESM1_ESM.pdf]

nature portfolio

https://doi.org/10.1038/s41586-023-05749-7

Supplementary information

Dehydration of a crystal hydrate at subglacial temperatures

In the format provided by the authors and unedited

Supplementary Materials for

Dehydration of a crystal hydrate at sub-glacial temperatures

Alan C. Eaby[1], Dirkie C. Myburgh[1], Akmal Kosimov[2], Marcin Kwit[2], Catharine Esterhuysen[1*], Agnieszka M. Janiak[2*] and Leonard J. Barbour[1*]

Correspondence to: ljb@sun.ac.za; agnieszk@amu.edu.pl; ce@sun.ac.za.

This PDF file includes:

Supplementary Text
Supplementary Figs. 1 – 71
Supplementary Tables 1 – 17
Captions for Supplementary Videos 1 – 11
References (79 – 178)

Table of Contents

Supplementary Table 1 3

Supplementary Text 8

Supplementary Text 1. Synthetic Procedures 8

Supplementary Text 2. Crystallization 9

Supplementary Text 3. Initial Crystallographic Assessment of T1 10

Supplementary Text 3.1. Crystal Structure at –173 °C 10

Supplementary Text 3.2. Crystal Structure at 25 °C 10

Supplementary Text 3.3. Initial Assessment of Crystallographic Porosity 12

Supplementary Text 4. Optical Microscopy of Crystals at Controlled RH 15

Supplementary Text 5. Gravimetric Water Vapor Sorption 15

Supplementary Text 5.1. Kinetic Measurements 15

Supplementary Text 5.2. Isotherm Measurements 21

Supplementary Text 5.3. Estimation of Adsorption Enthalpy 25

Supplementary Text 6. Simulated Water Uptake 34

Supplementary Text 6.1. Simulated Water Vapor Sorption Isotherms 34

Supplementary Text 7. Experimental Investigation of Tautomerism 41

Supplementary Text 7.1. Comparison of Salicylimine Bond Lengths 41

Supplementary Text 7.2. UV-vis Spectroscopy 45

Supplementary Text 7.3. Solid-State Nuclear Magnetic Resonance Spectroscopy 46

Supplementary Text 7.4. Attenuated Total Reflectance Fourier Transform Infrared Spectroscopy 49

Supplementary Text 8. Computational Study of Tautomerism 51

Supplementary Text 8.1. Density Functional Theory Orbital Calculation 51

Supplementary Text 8.2. Stabilization of the Ketoenamine Form in the Presence of Water 55

Supplementary Text 9. Single Crystal X-ray Diffraction Analysis of Red Crystals at 25 and –173 °C 60

Supplementary Text 10. Observation of Low-Temperature Water Loss 66

Supplementary Text 11. Variable Temperature Single-Crystal X-ray Diffraction Analysis 66

Supplementary Text 12. Differential Scanning Calorimetry 82

Supplementary Text 13. Kinetic Analysis of Low-Temperature Water Release 87

Supplementary Text 13.1. Microscopic Analysis of the Temperature-Dependence of the Rate of Dehydration 87

Supplementary Table 16 98

Supplementary Text 13.2 Computational Analysis of Diffusion 99

Supplementary Text 14. Commentary on Materials That May Possess a Low-Temperature T_{on}. 104

Captions for Supplementary Videos 106

References 108

Supplementary Table 1. Examples of channel hydrates with their published dehydration temperatures (where available) as determined by TGA at 1 atm.

Reference	Composition	Water loss temperature (°C)
79	(His-Tyr-Phe)·6.08(H_2O)	not reported
	(Asp-Tyr-Phe-NH_2)·3(H_2O)	not reported
	(Tyr-Phe-Asp-NH_2)·2(H_2O)	not reported
5	N-(pyridin-4-ylmethylene)quinolin-5-amine·H_2O	30-90
	N,N'-(1H-Imidazol-1-yl)-N-(pyridin-4-ylmethylene)aniline·H_2O	50-90
	N,N'-(1,4-phenylenebis(methan-1-yl-1-ylidene))diquinolin-5-amine·4(H_2O)	50-120
80	{[Ru(H_2bim)$_3$](TATC)·20(H_2O)}$_n$	177
	{[Ru(H_2bim)$_3$](TATC)·20(H_2O)}$_n$	137
81	{[Ru(H_2bim)$_3$](TMA)·20(H_2O)}$_n$	105
82	β-cyclodextrin·12(H_2O)	30-100
83	NiNbOF$_5$(pyr)$_2$·2(H_2O)	30-150
84	4-Nitrostyrylpyridine hydrochlorid·3H_2O	20-47
85	{[Ni(cyclam)]$_3$(TMA)$_2$·35-34H_2O}n	37-90
86	{[Zn(mip)(3-pna)]·2H_2O}$_n$	90-120
87	[Co(H_2bim)$_3$](TATC)·H_2O}$_n$	not reported
88	Modafinil·2(H_2O)	100
89	{[Cu(atc)(H_2O)]·H_2O}$_n$	25-245
90	{[Co(H_2bim)$_3$](TMA)·20H_2O}$_n$	150

Supplementary Table 1. continued

Reference	Composition	Water loss temperature (°C)
91	$[Co(H_2bim)_3](TMA){\cdot}21.8H_2O$	not reported (dehydration under vacuum at 200°C for 28 h)
	$[Ru(H_2bim)_3](TMA){\cdot}20.7H_2O$	
92	$\{[Co(H_2bim)_3](TMA){\cdot}20H_2O\}_n$	150
	$\{[Ru(H_2bim)_3](TMA){\cdot}20H_2O\}_n$	200
	$\{[Rh(H_2bim)_3](TMA){\cdot}20H_2O\}_n$	100
93	Peptide cyclo[(D-^{Me}N-Ala-L-γ-Ach)$_4$-]	not reported
94	$[Ag_2(bpp)_2(suc)\ {\cdot}12H_2O]_n$	25-105
	$[Ag_2(bpe)_2(suc)\ {\cdot}9H_2O]_n$	25-108
95	$\{[Ru(H_2bim)_3](TMA){\cdot}20H_2O\}_n$	200
96	$\{[Co(H_2bim)_3](TMA){\cdot}20H_2O\}_n$	not reported (the mass loss is reported but not temperature)
97	$\{[Zn_2(TPOM)(bdc)_2]_3{\cdot}4(H_2O)\}_n$	20-140
	$\{[Zn_2(TPOM)(bdc)_2]_3{\cdot}(H_2O)\}_n$	20-180
	$\{[Co_2(TPOM)(5\text{-}OH\text{-}bdc)_2(H_2O)_2]_3{\cdot}5(H_2O)\}_n$	20-170
	$\{[Cd_2(TPOM)(5\text{-}OH\text{-}bdc)_2]_3{\cdot}2(H_2O)\}_n$	20-110
98	$[Cr(H_2bim)_3](TMA){\cdot}23.5(H_2O)$	200 (under vacuum)
99	$Na_5[Ho(THB^{4-})_2{\cdot}7H_2O$	not reported
100	β-cyclodextrin$\cdot 7.5(H_2O)$	not reported

Supplementary Table 1. continued

Reference	Composition	Water loss temperature (°C)
101	$\{[Cd_3(phen)_3(HL)_2(H_2O)_2]\cdot 4.25(H_2O)\}_n$	25-146
102	$[Yb_2(pztc)_{1.5}(H_2O)_6]\cdot 7(H_2O)$	30-250
103	$[Ni(phen)_3](C_{10}H_{16}O_4)\cdot(CO_{10}H_{18}O_4)_{0.5}\cdot 11H_2O$	48-146
104	$[Ru(H_2bim)_3(TMA)]\cdot DMF\cdot 9H_2O$	20-170 (loss of one DMF and nine water molecules)
105	$[Zn_6(IDC)_4(OH)_2(Hprz)_2]\cdot 13H_2O$	35-70
106	$\{[Ca(AIP)(H_2O)_4]\cdot H_2O\}_n$	30-91
107	$[Cu_2(phen)_2(CH_3COO)(CH_3COOH)(H_2O)_2][Al(OH)_6Mo_6O_{18}]\cdot 28H_2O$	25-100
108	(E)methyl 3-(4-((1H-imidazol-1-yl)methyl)phenyl)acrylate · 2(H_2O)	40-120
	(E)-ethyl 3-(4-((1H-imidazol-1-yl)methyl)phenyl)acrylate ·2(H_2O)	45-115
109	(dec-acid)(α-CD)$_2$·25(H_2O)	not reported
110	$[Cu_2(PNO)_2(H_2O)_6]\cdot[(H_6TCAS)_2]\cdot 22H_2O$	30-250
111	$[Ni(cyclam)(H_2O)_2]_3(TMA)_2\cdot 24H_2O$	not reported (dehydration under vacuum at 24°C)
112	$[Co(H_2bim)_3](TMA)\cdot 20H_2O$	20-105
	$[Co(H_2bim)_3](TMA)\cdot 20D_2O$	20-98
113	Trianglamine·6(CH_3OH)·0.5(Br_2)·11.5(H_2O)	not reported
114	proximal calix[4]arene dihydroquinone ·3.23(H_2O)	25-75
115	$\{Zn_2(ptaH)_2\cdot 11H_2O\}_n$	80-270
116	$C_8H_7N_3O_3\cdot 0.03(H_2O)$	not reported

Supplementary Table 1. continued

Reference	Composition	Water loss temperature (°C)
117	$[Ni(Me_2tsc)_2(H_2O)_2][fumarate]\cdot H_2O$	80-150 (no distinction between coordinated and lattice water)
	$[Ni(Me_3tsc)_2(H_2O)_2][isophthalate]\cdot 3.2H_2O$	50-130 (no distinction between coordinated and lattice water)
	$[Ni(Me_2tsc)_2(itaconate)]\cdot 6H_2O$	40-150
118	$\{[Yb(PDA)_3Mn1.5(H_2O)_3]\cdot 1.5H_2O\}_n$	20-120
119	sym-(hydroxy)dibenzo-14-crown-4	not reported
2	Cephalexin 1.9(H_2O)	not reported
	Raffinose · 5D_2O	not reported (dried in vacuum at 60°C)
	Trehalose · 2D_2O	not reported (dried in vacuum at 60°C)
120	Theophylline · H_2O	not reported
	Caffeine · 4/5H_2O	not reported
	Sodium cromoglycate· 7-7.8H_2O	not reported
121	per-2,6-OMe-β-CD·14.7(H_2O)	not reported
	per-2,6-OMe-β-CD · 1.08(H_2O)	not reported
122	Cyclodecaamylose · 27.18(H_2O)	not reported
123	$\{[Cu_3(TMA)_2(H_2O)_3]\cdot 10(H_2O)\}_n$	100 (dehydration with a colour change from turquoise to dark blue)
124	octakis(2,3,6-tri-O-methyl)-γ-cyclodextrin·19.3(H_2O)	not reported

Supplementary Table 1. continued

Reference	Composition	Water loss temperature (°C)
125	i-Cyclodextrin · 9(H_2O)	not reported
126	β-cyclodextrin·10.7(H_2O)	30-100
127	β-cyclodextrin·12.3(H_2O) (at 100% RH)	not reported
128	β - cyclodextrin·11(H_2O)	not reported
129	$C_{24}H_{30}0_8$ · [$LaCl_{2.5}(OH)_{0.5}$]·4.5H_2O	not reported
130	β - cyclodextrin·12(H_2O)	not reported
131	α-cyclodextrin·7.57(H_2O)	not reported

Supplementary Text

Supplementary Text 1. Synthetic Procedures

The general synthetic procedure is presented in Supplementary Scheme 1. All spectra are in accordance with literature data[132–134].

2,5-Dimethoxyterephthalaldehyde was obtained from 1,4-dimethoxybenzene according to the previously published procedure[134].

m.p. 214 – 214.5 °C (lit. 193 – 195 °C)[134];

^{1}H NMR (400 MHz, $CDCl_3$): δ = 10.50 (s, 1H), 7.45 (s, 1H), 3.94 (s, 3H);

^{13}C NMR (75.5 MHz, $CDCl_3$): δ = 189.21, 155.71, 129.10, 110.88, 56.20;

IR (ATR): ṽ = 3336, 3052, 2990, 2955, 2870, 2761, 1668, 1480, 1393, 1301, 1210, 1127, 1017, 876, 657 cm^{-1}.

2,5-Dihydroxyterephthalaldehyde was obtained from 2,6-dimethoxyterephthalaldehyde according to the previously published procedure[132].

m.p. 169 – 169.5 °C;

^{1}H NMR (300 MHz, $CDCl_3$): δ = 10.23 (s, 2H), 9.96 (s, 2H), 7.24 (s, 2H);

^{13}C NMR (75.5 MHz, $CDCl_3$): δ = 196.42, 153.26, 125.19, 121.61;

IR (ATR): ṽ = 3487, 3264, 3053, 2890, 1663, 1475, 1459, 1277, 1122, 888, 832, 792, 665, 507 cm^{-1}.

Trianglimine T1 was obtained according to a slightly modified published procedure[133]. A solution of *trans*-(1*R*,2*R*)-diaminocyclohexane (56 mg, 0.5 mmol), 2,5-dihydroxy-terephthalaldehyde (81.5 mg, 0.5 mmol) and $CHCl_3$ (25 mL) was stirred under argon atmosphere at room temperature for 7 days. The mixture was then evaporated to dryness providing **T1** in quantitative yield. The product crystallised from ethanol solution as a yellow-orange solid.

m.p. decomposed above 300 °C;

^{1}H NMR (400 MHz, $CDCl_3$): δ=1.45-1.86 (m, 4H), 3.30 (m, 1H), 6.68 (s, 1H), 8.16 (s, 1H), 12.25 (s, 1H);

^{13}C NMR (400 MHz, $CDCl_3$): δ= 24.17, 32.95, 73.82, 118.37, 121.00, 152.49, 163.92;

MS (HR ESI-TOF^{+}): m/z found 733.3709 $[M+H]^{+}$, calcd $C_{42}H_{49}N_6O_6$ 733.3714;

$[\alpha]^{20}_{D}$ -407.1 (c = 1, $CHCl_3$);

IR (ATR): $\tilde{\nu}$ = 810.81, 854.50, 1040.78, 1097.74, 1157.78, 1215.59, 1309.96, 1362.40, 1448.25, 1510.23, 1622.36, 2653.40, 2700.34, 2858.07, 2928.19 cm^{-1}

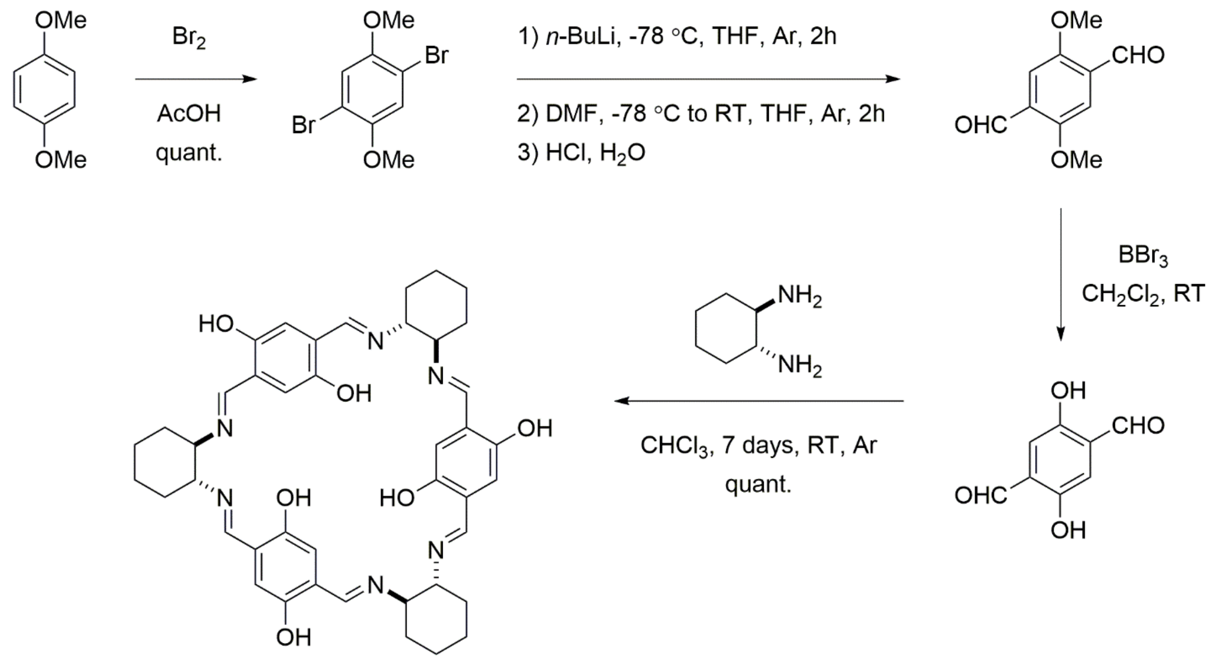

Supplementary Scheme 1. Synthesis of T1.

<u>Supplementary Text 2. Crystallization</u>

T1 (*ca*. 15mg) was dissolved in a 1:1 mixture of absolute ethanol (1 mL) and chloromethane (1mL). The solution thus obtained was slowly evaporated at room temperature and relative humidity not exceeding 40%. T1 initially formed a gel, which later liquified and deposited trigonal prismatic crystals (Supplementary Scheme 2 and Supplementary Fig. 1).

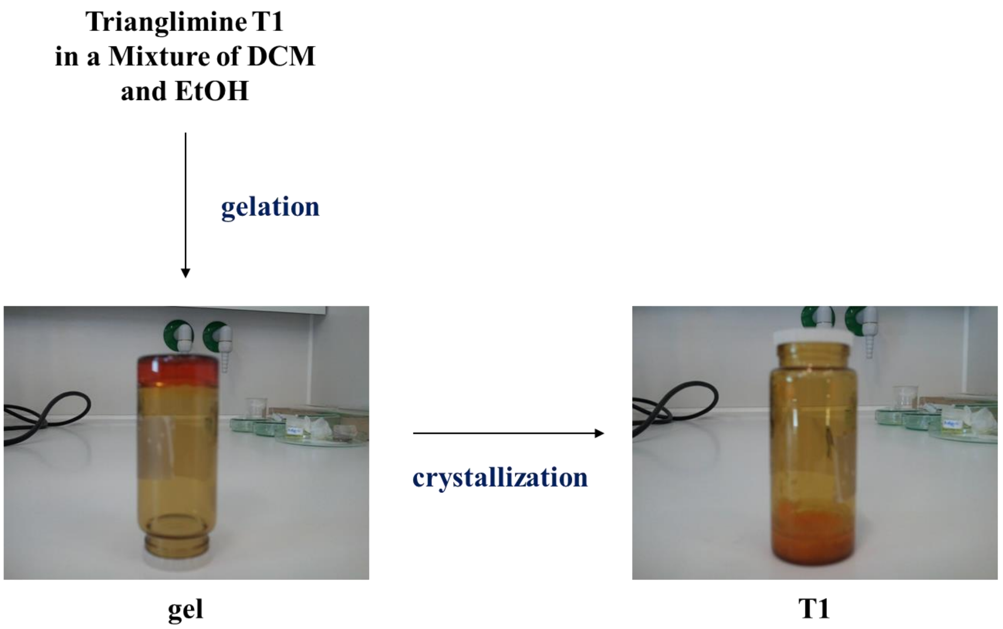

Supplementary Scheme 2. Crystallization of **T1**.

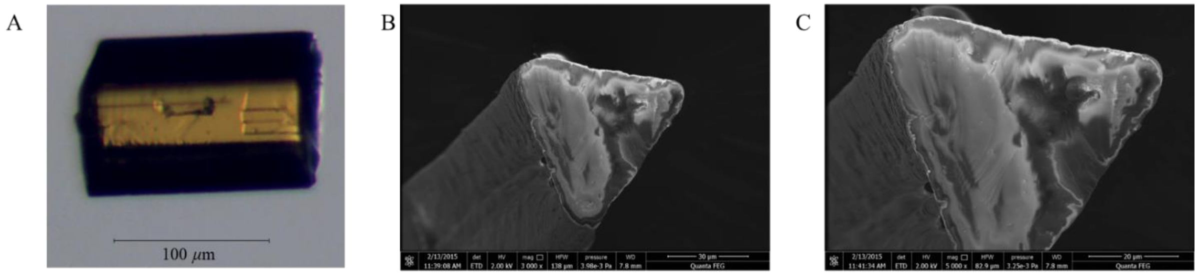

Supplementary Fig. 1. (A) Photomicrograph of a yellow single crystal of **T1**. (B and C) Scanning electron micrographs showing the morphology of the trigonal crystals.

Supplementary Text 3. Initial Crystallographic Assessment of T1

Supplementary Text 3.1. Crystal Structure at –173 °C

Structure solution yielded electron density peaks consistent with the non-hydrogen atoms of two T1 host molecules in the asymmetric unit. These atoms were refined anisotropically and hydrogen atoms were placed using riding models. After convergence of the atoms of the two host molecules, a residual peak of 0.12 e^{-} Å^{-3} emerged as the most intense feature in the difference electron density map. Crystallographic parameters relevant to the final refinement are listed in Supplementary Table 2. The atomic labelling scheme is shown in Supplementary Fig. 2.

Supplementary Text 3.2. Crystal Structure at 25 °C

During intensity data collection the crystal was immersed in a temperature-controlled stream of dry nitrogen gas (i.e., *ca* 0% relative humidity). Structure solution and refinement proceeded as for the data recorded at –173 °C. After convergence of the host molecules, the height of the largest residual peak was 0.15 e^{-} Å^{-3}. Crystallographic parameters relevant to the final refinement are listed in Supplementary Table 2.

Supplementary Table 2. Selected crystallographic data for **T1-Y** (yellow) and **T1-R** (red) crystals at 25 °C and –173 °C.

Structure	**T1-Y**		**T1-R**	
Temperature (°C)	–173	25	–173	25
Chemical formula	$C_{42}H_{48}N_6O_6$	$C_{42}H_{48}N_6O_6$	$C_{42}H_{48}N_6O_6 \cdot 3.5H_2O$	$C_{42}H_{48}N_6O_6 \cdot 3.5H_2O$
Formula weight	732.86	732.86	795.92	795.92
Space group	Trigonal, *R*3:H	Trigonal, *R*3:H	Trigonal, *R*3:H	Trigonal, *R*3:H
a (Å)	50.3926(7)	50.6673(15)	51.0014(19)	50.5800(30)
b (Å)	50.3926(7)	50.6673(15)	51.0014(19)	50.5800(30)
c (Å)	9.4039(2)	9.6414(3)	9.2632(5)	9.6866(7)
α (°)	90	90	90	90
β (°)	120	120	120	120
γ (°)	90	90	90	90
Z	18	18	18	18
V (Å^3)	20681.0 (7)	21435.1(14)	20866.8(19)	21462(3)
D_{calc} (g cm^{-1})	1.023	1.022	1.140	1.1083
N_{total}	76862	188245	71426	48850
N_{ind}	18574	19501	23394	19348
N_{obsd}	12550	9616	18094	6326
R_1 ($I > 2\alpha(I)$)	0.087	0.087	0.102	0.095
R_{int}	0.110	0.100	0.049	0.084
wR_2	0.182	0.214	0.288	0.305
GooF	1.14	1.04	1.05	0.95
μ (mm^{-1})	0.07	0.07	0.08	0.08

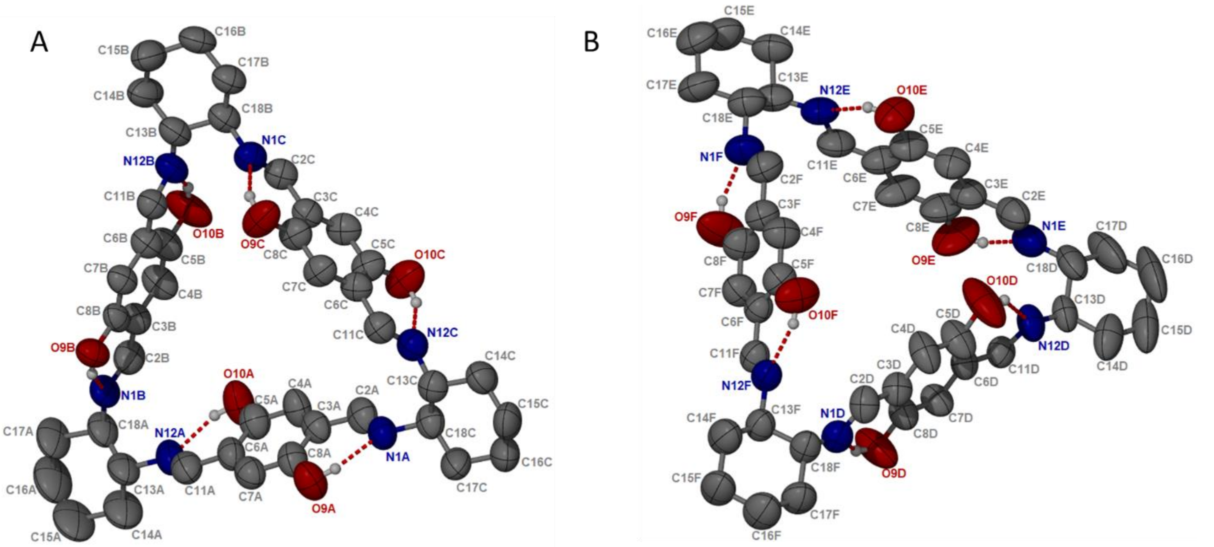

Supplementary Fig. 2. Atomic displacement plots and atomic numbering scheme of the two molecules of T1 in the asymmetric unit at –173 °C. Ellipsoids are shown at the 50% probability level. Except for hydroxyl groups, hydrogen atoms have been omitted for clarity. Colours: grey, carbon; red, oxygen; white, hydrogen.

Supplementary Text 3.3. Initial Assessment of Crystallographic Porosity

A packing diagram of **T1-Y** (Supplementary Fig. 3A) reveals 1 nm wide extrinsic channels propagating along the crystallographic *c* axis. Each of the host molecules also encloses an intrinsic cavity that is seemingly inaccessible from the channels. The probe-accessible volumes (Supplementary Fig. 3B) were calculated using the program MSRoll[135]. At 25 °C each of the channels occupies a volume of 1016 Å^3 per unit cell and the volumes of the two unique intrinsic cavities are 40 and 49 Å^3. At –173 °C the channels also occupy a volume of 1016 Å^3 per unit cell and the volumes of the two unique intrinsic cavities are 39 and 50 Å^3. Only 3 of the 12 symmetry-independent OH groups are exposed to the interior of the channel (Supplementary Fig. 4), where they serve as hydrophilic sites for possible host-guest interactions.

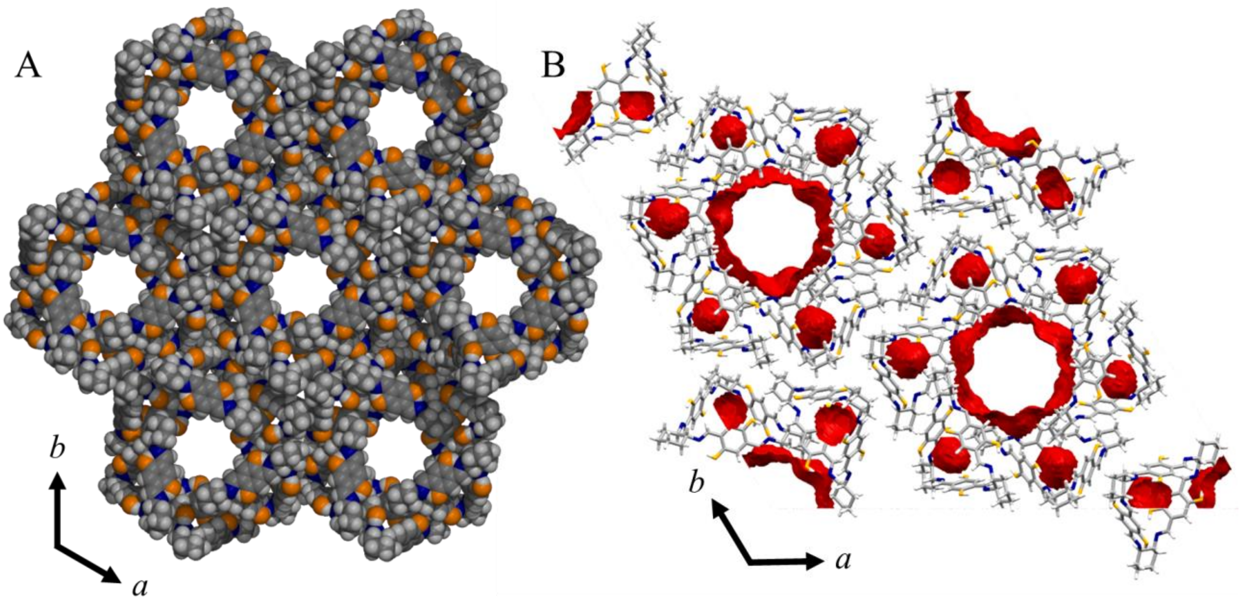

Supplementary Fig. 3. 1D channels in the crystal structure of **T1-Y**, as viewed along [00$\bar{1}$]. (A) Space filling model of **T1-Y** showing the trigonal arrangement and 1D channels propagating along the crystallographic *c* axis. (B) Capped-stick model of **T1-Y** showing the probe-accessible surface generated by Mercury[136] using a probe radius of 1.5 Å and grid spacing of 0.2 Å. The intrinsic cavities of the T1 molecules are isolated and seemingly inaccessible from the extrinsic channels. Colours: red, probe-accessible surface; grey, carbon; white, hydrogen; blue, nitrogen; orange, oxygen.

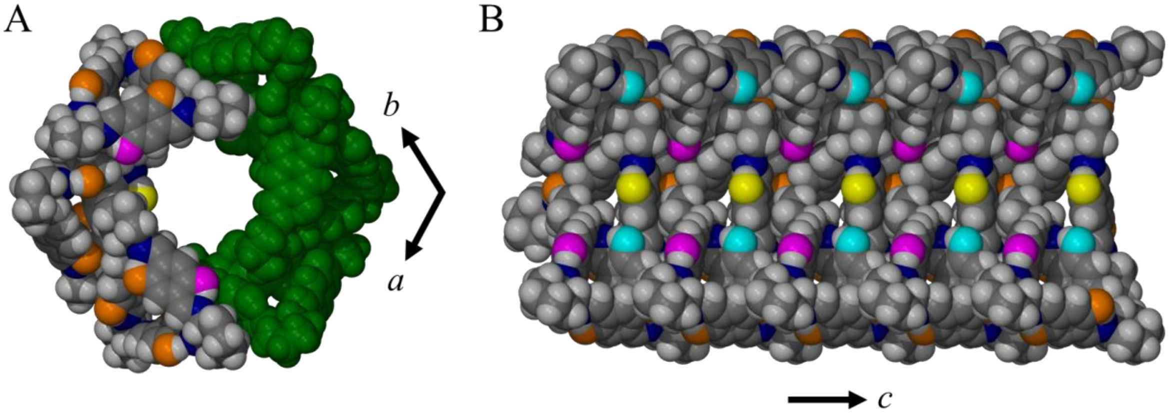

Supplementary Fig. 4. Exposed hydroxyl groups lining the channel walls of **T1-Y**. (A) A projection along the channel axis [001] and (B) a perspective view along [120] with host molecules in the foreground omitted. Three crystallographically unique hydroxyl groups (coloured yellow, cyan and magenta) protrude into the channels, thus forming potential hydrogen bonding acceptor sites for binding guest molecules. Colours: yellow, O9D; cyan, O9B; magenta, O9A; orange, remaining host oxygen atoms; grey, carbon; white, hydrogen; green, omitted host molecules. Note: O9B is obscured by a salicylimine moiety in (A) and cannot be seen in this projection.

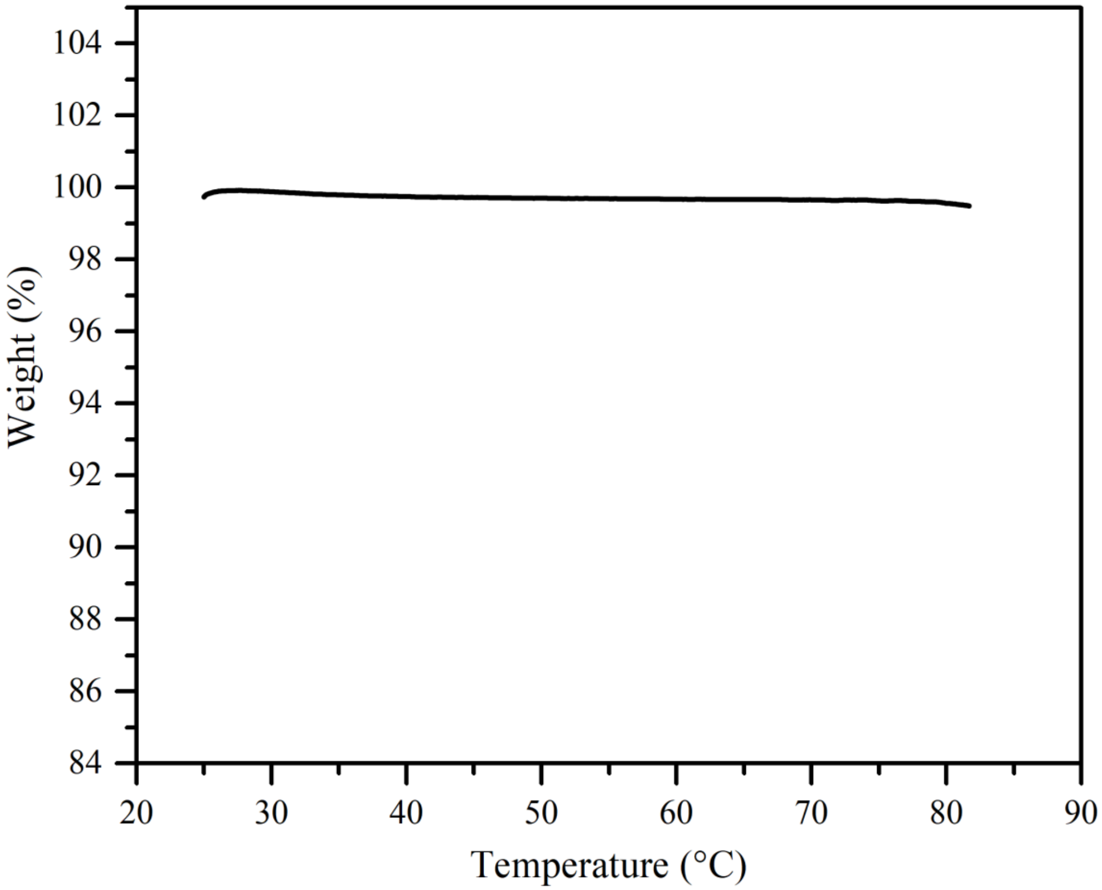

Supplementary Fig. 5. TGA of **T1-Y**. The sample was equilibrated in the furnace at 25 °C and heated to 80 °C at a rate of 10 °C min^{-1}.

Supplementary Text 4. Optical Microscopy of Crystals at Controlled RH

We noticed that the crystals were yellow on some days and red on others, and that these colour changes were correlated to fluctuations in RH within our laboratory, with the transition occurring between 50 and 60%. These observations were confirmed by optical microscopy (Figs. 2a and 2c); a single crystal was placed on a microscope slide and observed in an airconditioned room at 45% RH and 20 °C. When a circular strip of water-soaked cotton wool was placed around the crystal, thus saturating its immediate environment with water vapor, the initially yellow crystal rapidly became red (Supplementary Video 1). Moreover, the colour transition occurred as two red fronts emanating from the opposite ends of the elongated crystal and progressing towards its centre along the channel axes [001] and [00$\bar{1}$]. When the wet cotton wool was removed, the crystal gradually reverted to yellow, with the colour change again progressing along [001] and [00$\bar{1}$] (Supplementary Video 2).

Two yellow crystals of **T1** were placed on a circular glass cover slip of diameter 22 mm, which was in turn placed on the sample stage of a stereo zoom optical microscope (Zeiss Stemi 508). The temperature and relative humidity of the laboratory were approximately 25 °C and 40%. A brass cylinder (diameter 40 mm, height 15 mm) with a clear glass window was placed over the cover slip to provide a semi-hermetic environment. A small dish containing a saturated solution of $Mg(NO_3)_2$ was inserted into the assembly to adjust the relative humidity around the sample to 53%[137]. The crystals remained yellow for 30 minutes. The salt solution was then replaced by a saturated solution of NaBr (58% RH at 25 °C)[137] and time-lapse photographs were recorded at intervals of 30 seconds (Supplementary Video 3). The crystals changed colour from yellow to red over a period of approximately 10 to 15 minutes. These visual experiments were carried out repeatedly, with no discernible degradation of the crystals over multiple cycles.

Supplementary Text 5. Gravimetric Water Vapor Sorption

Supplementary Text 5.1. Kinetic Measurements

The rates of adsorption (k_a) and desorption (k_d) of water vapor by powdered **T1** were quantified at 25 °C by means of a gravimetric vapor balance system[138]. For each adsorption experiment, the initial RH was regulated at 11% using a saturated solution of LiCl[137]. By replacing the salt solution with one of NaBr, KI, NaCl, KBr, KCl or KNO_3 the RH was increased to 58, 69, 75, 81, 84 or 92%, respectively. In each case the process was reversed for desorption. Supplementary Figs. 6 and 7 show plots of the extent of reaction α ($\alpha \in [0;1]$) for adsorption α_a and desorption α_d, respectively. These data were interpreted using the method proposed by Brown[139] based on the kinetic theories of Avrami[140]. The reaction progress for adsorption α_a and desorption α_d are defined as:

$$\alpha_a = \frac{(m_t - m_0)}{(m_\infty - m_0)}\ ,\ \alpha_d = 1 - \frac{(m_t - m_0)}{(m_\infty - m_0)}$$

where m_0 is the initial mass, m_t is the adsorbate mass at time t and m_∞ is the mass at equilibrium. The values of k_a and k_d were determined by fitting an appropriate conversion function (Supplementary Figs. 8 and 9, respectively) to the α–t data:

$$f(\alpha) = 1 - \sqrt{1 - \alpha}$$

The magnitude of the change in RH (i.e., difference between the initial and final RH, ΔRH), appeared to directly influence the values of k_a and k_d (Supplementary Fig. 10 and Supplementary Table 3); an increase in ΔRH resulted in a corresponding increase in the value of k_a, whereas the value of k_d decreased with an increase in ΔRH. If the transport of water in the channels is the rate determining process, then the value of k_a should be constant at each ΔRH step[141]. The difference is due to differences in the rate of diffusion of water vapor from the salt solution at higher ΔRH. Water vapor has a higher chemical potential at higher ΔRH and the threshold value of 55% RH is reached in a shorter period of time. The opposite is observed for k_d, where higher ΔRH values yield slower desorption times.

The values of k_a and k_d are also influenced by the slow diffusion of water from the surface of the sample to the centre of the sample. This delay was observed by monitoring the change in colour of the sample during adsorption and desorption within the vapor balance (Supplementary Fig. 11). During adsorption, the colour change of the sample occurred after 2 min. However, this corresponded to α_a = 0.21, which implies that 22% of sample has adsorbed water vapor to *ca* 7.3 wt% (most likely the outer particles of the powdered sample). This is slow when compared with the rates of adsorption determined by measuring the progress of the red boundary in a single crystal (Supplementary Videos 1 and 2). Unlike the gravimetric experiment, the increase in RH was almost immediate in the latter experiment. Moreover, the (001) and (00$\bar{1}$) faces of the single crystal were not obstructed and it adsorbed moisture as it became available. The rate of desorption in the bulk sample similar to that of the single crystal since the RH was more rapidly reduced below the threshold value of 55%. The colour change to yellow of the surface of the bulk sample occurred within 100 s, corresponding to α_d = 0.28 (i.e., 28% of the sample had reached *ca* 0.5 wt% water).

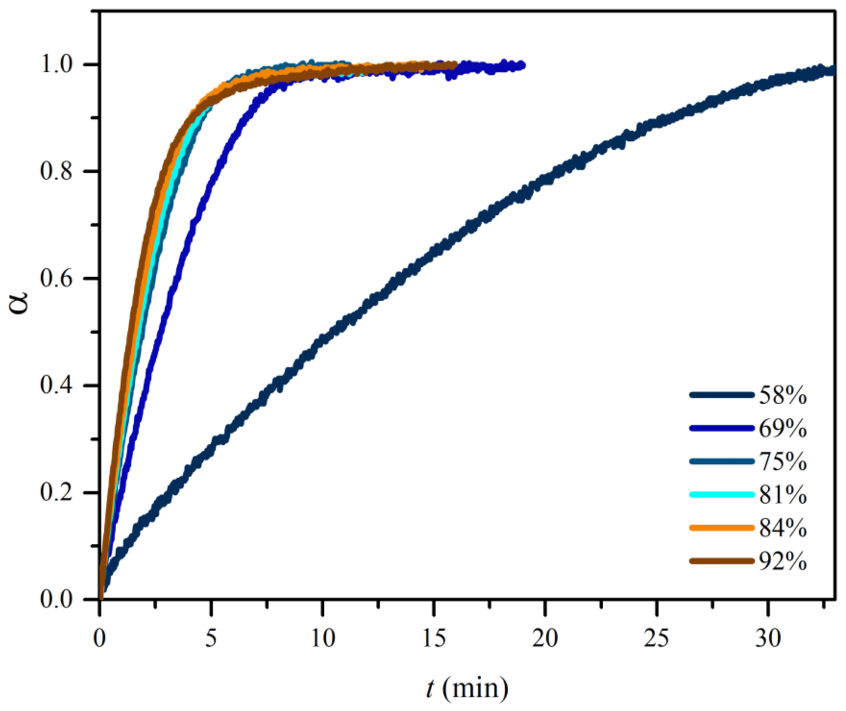

Supplementary Fig. 6. Relaxation plots for adsorption at 25 °C between 11% RH and the target RH shown in the legend.

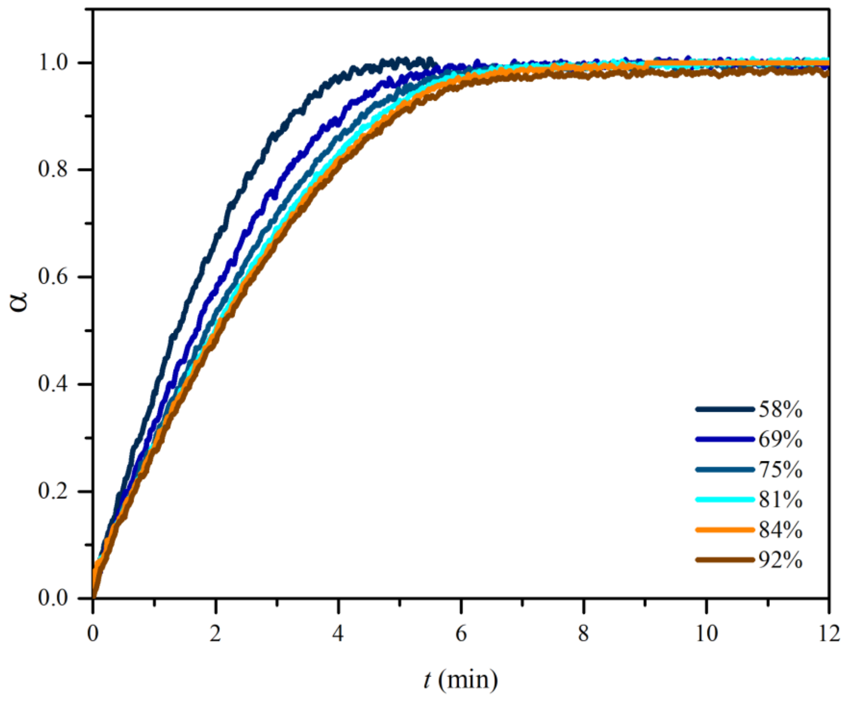

Supplementary Fig. 7. Relaxation plots for desorption at 25 °C between the RH shown in the legend, and 11% RH.

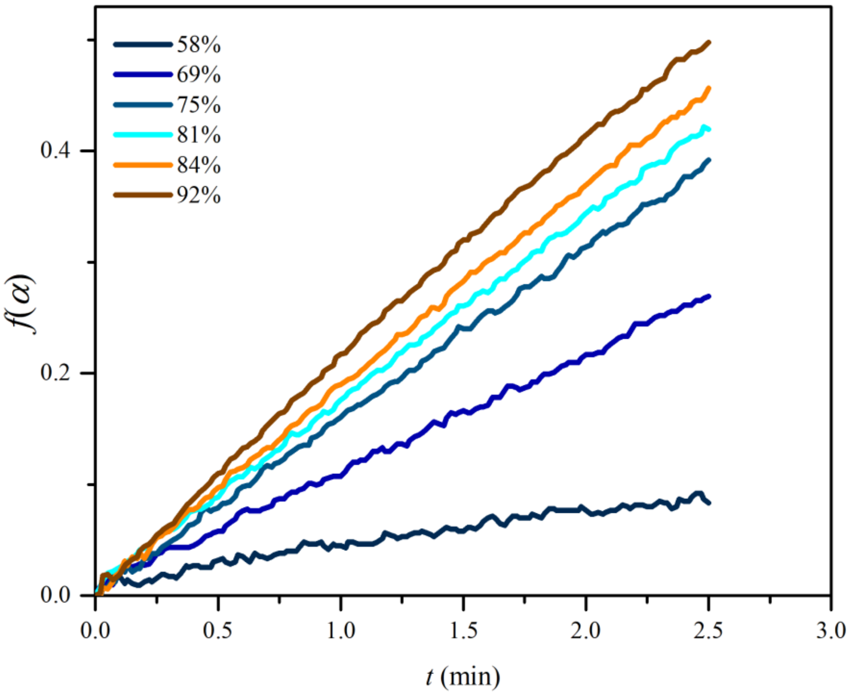

Supplementary Fig. 8. Plots of the linear regions of the conversion function $f(\alpha)$ for adsorption at 25 °C between 11% RH and the target RH shown in the legend.

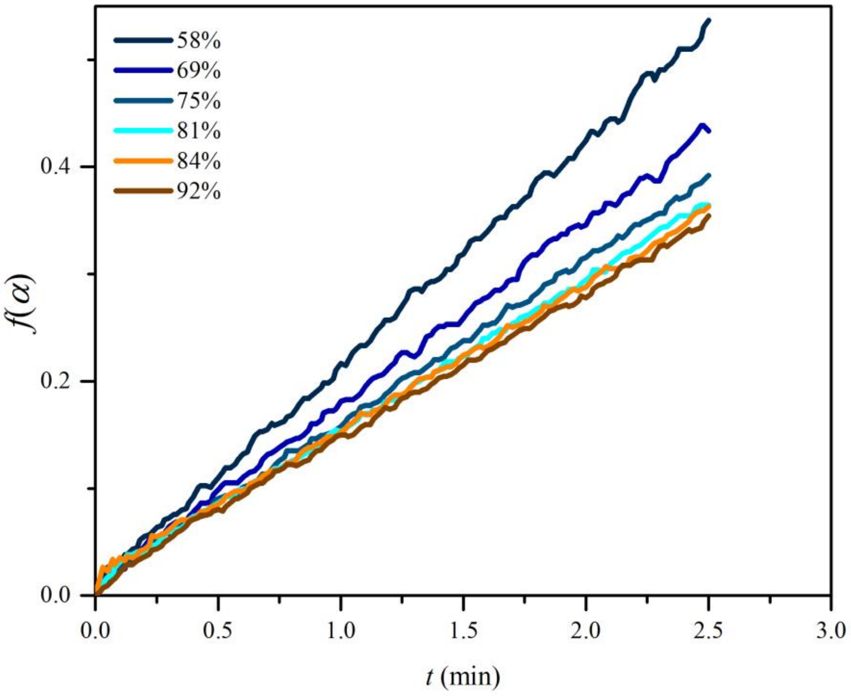

Supplementary Fig. 9. Plots of the linear regions of the conversion function $f(\alpha)$ for desorption at 25 °C between the RH shown in the legend and 11% RH.

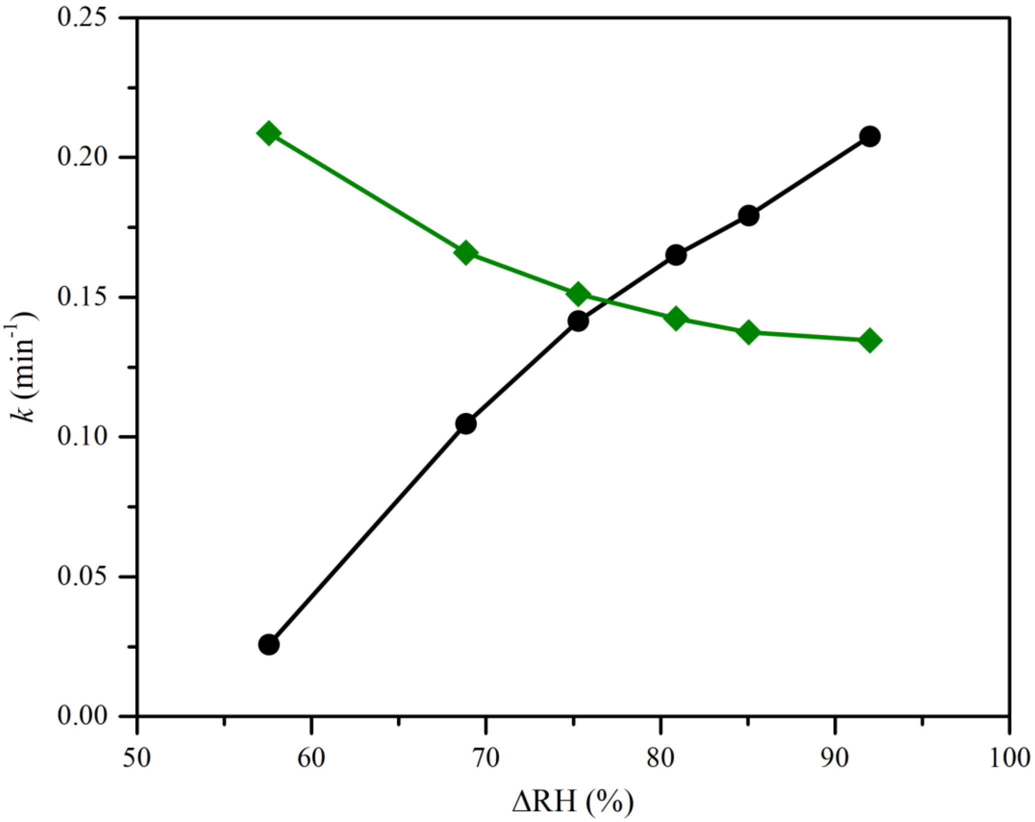

.

Supplementary Fig. 10. Plots of k_a (black, circles) and k_d (green, diamonds) *vs* ΔRH for **T1** at 25 °C.

Supplementary Table 3. Rate constants k_a and k_d for water vapor sorption experiments carried out on **T1**.

ΔRH (%)	58	64	70	73	81
Final RH (%)	69	75	81	84	92
k_a (× 10^{-2} min^{-1})	1.05 ± 0.02	1.42 ± 0.03	1.65 ± 0.02	1.79 ± 0.03	2.08 ± 0.05
k_d (× 10^{-1} min^{-1})	1.66 ± 0.01	1.51 ± 0.01	1.42 ± 0.01	1.37 ± 0.01	1.35 ± 0.01

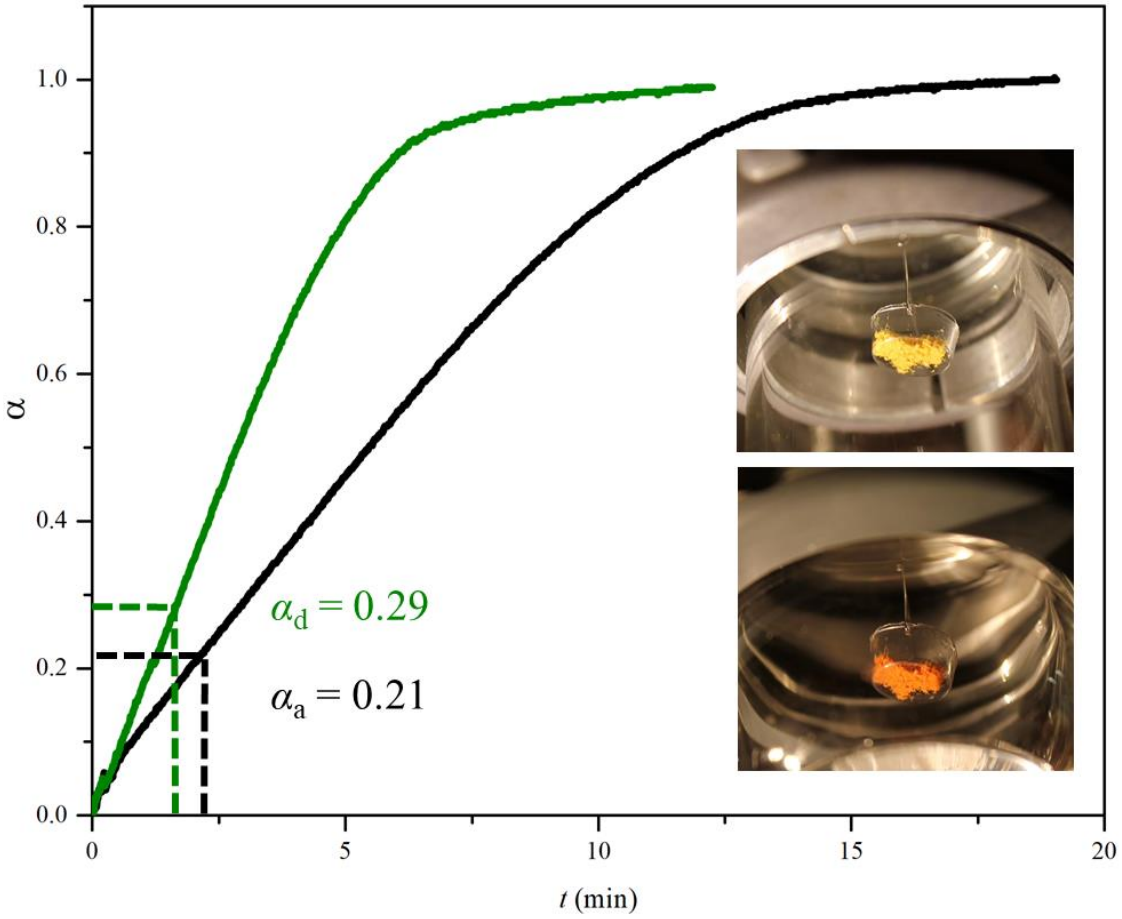

Supplementary Fig. 11. Relaxation plots for adsorption (black) and desorption (green) of water at 25 °C by **T1** at a starting RH of 11% and a final RH of 75%. The value of α_a and α_d at the onset of the colour change as determined by visual inspection is indicated by the dotted lines. Inset: images of the anhydrous (top) and hydrous (bottom) crystals on the vapor balance.

Supplementary Text 5.2. Isotherm Measurements

Supplementary Figs. 12 to 15 show isotherms at 10, 25 and 40 °C, confirming that **T1-Y** retained its water sorption capability over multiple cycles and a range of temperatures. Based on the crystallographic and DVS data, water occupies 42% of the accessible van der Waals space within the host channels at full loading, which corresponds to a water density of 0.64 g cm^{-3}. Although lower than the approximately 51(4)% guest occupancy expected for porous crystals[142], nanoconfined water is typically less dense than bulk water, with its density decreasing with a decrease in pore size[143,144].

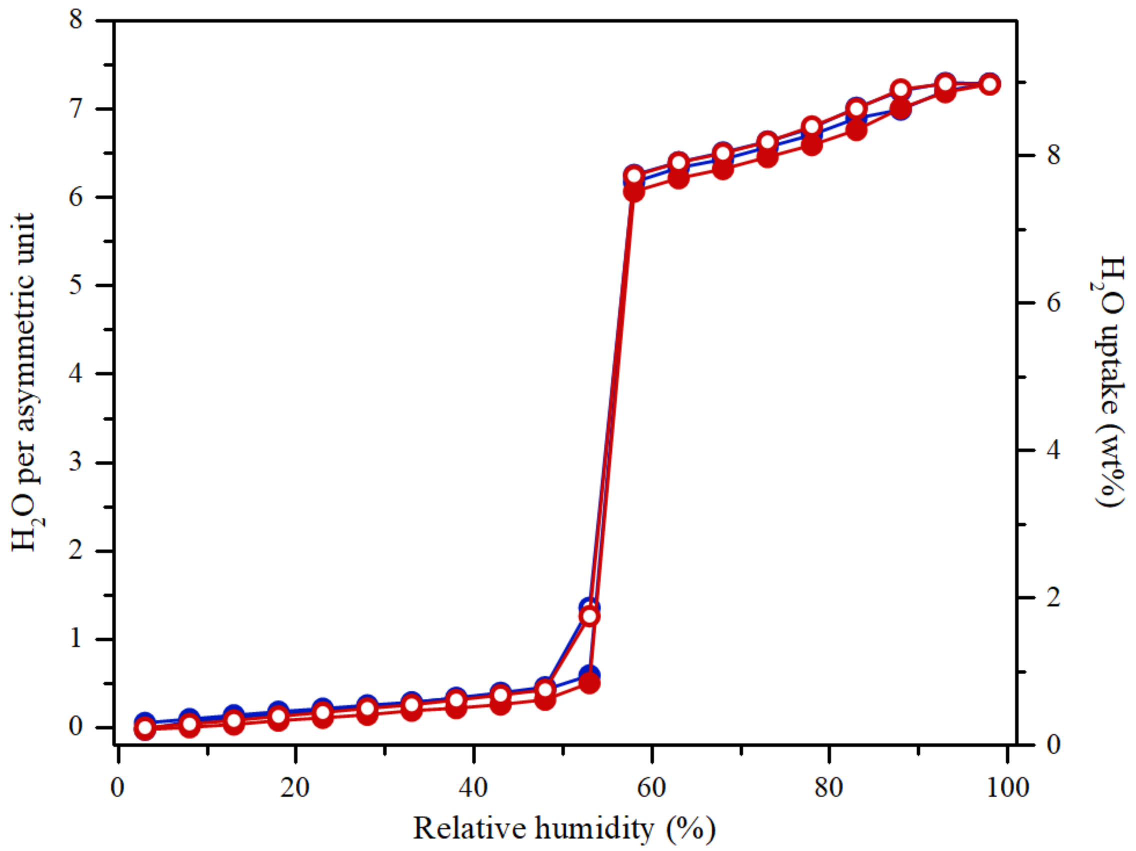

Supplementary Fig. 12. Two consecutive water vapor adsorption (filled circles) and desorption (open circles) cycles for **T1**, recorded at 10 °C using a dynamic vapor sorption analyser.

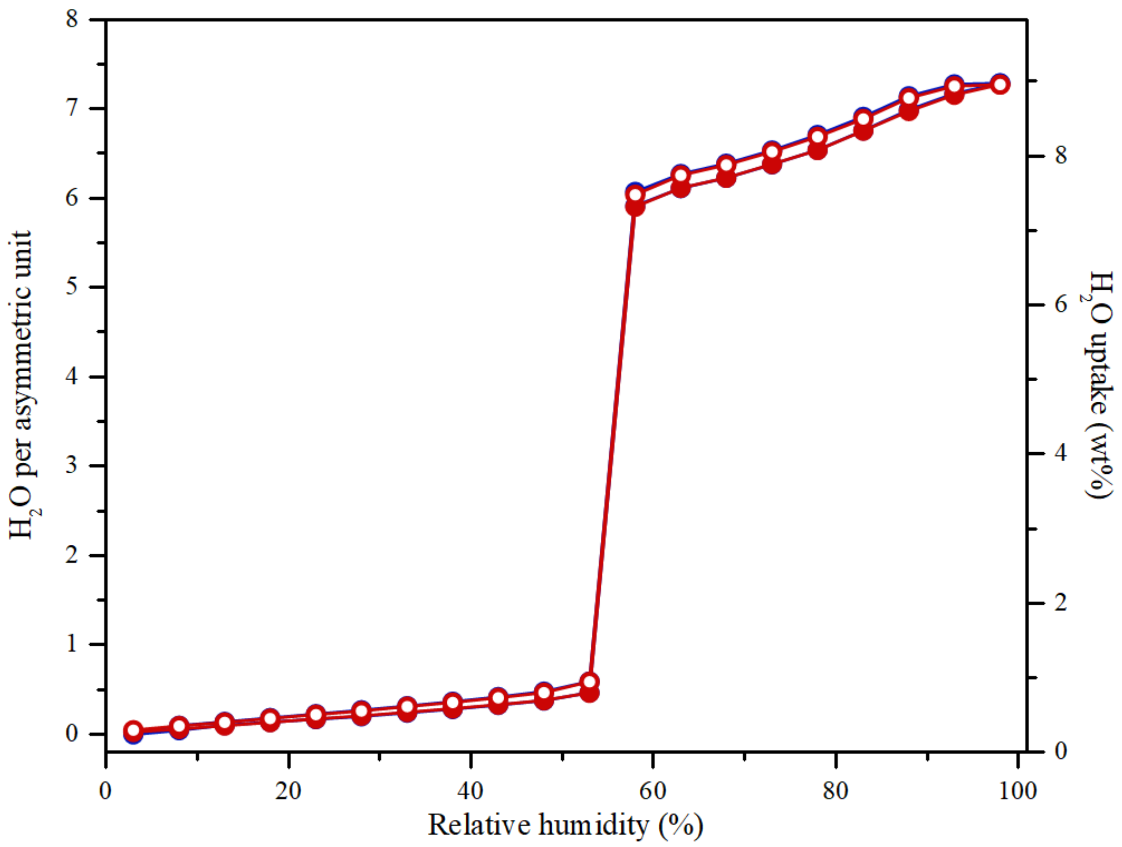

Supplementary Fig. 13. Two consecutive water vapor adsorption (filled circles) and desorption (open circles) cycles for **T1**, recorded at 25 °C using a dynamic vapor sorption analyser.

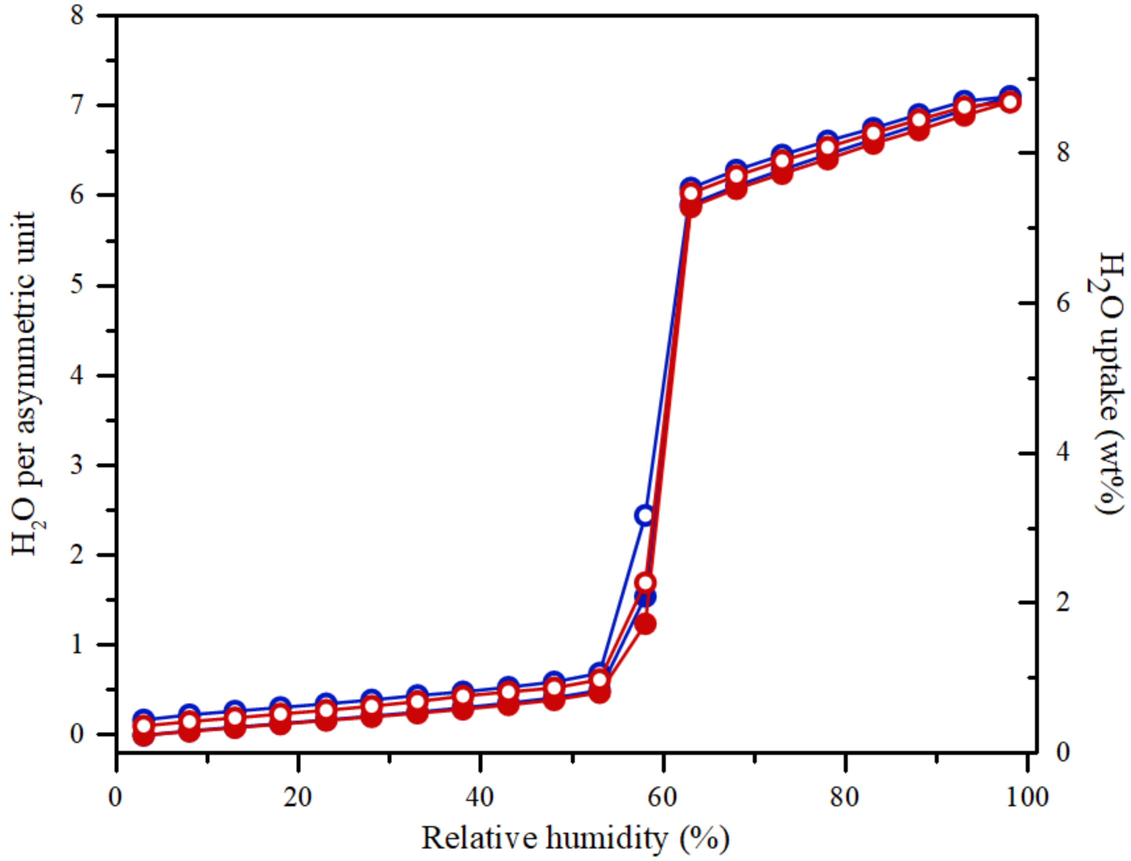

Supplementary Fig. 14. Two consecutive water vapor adsorption (filled circles) and desorption (open circles) cycles for **T1**, recorded at 40 °C using a dynamic vapor sorption analyser.

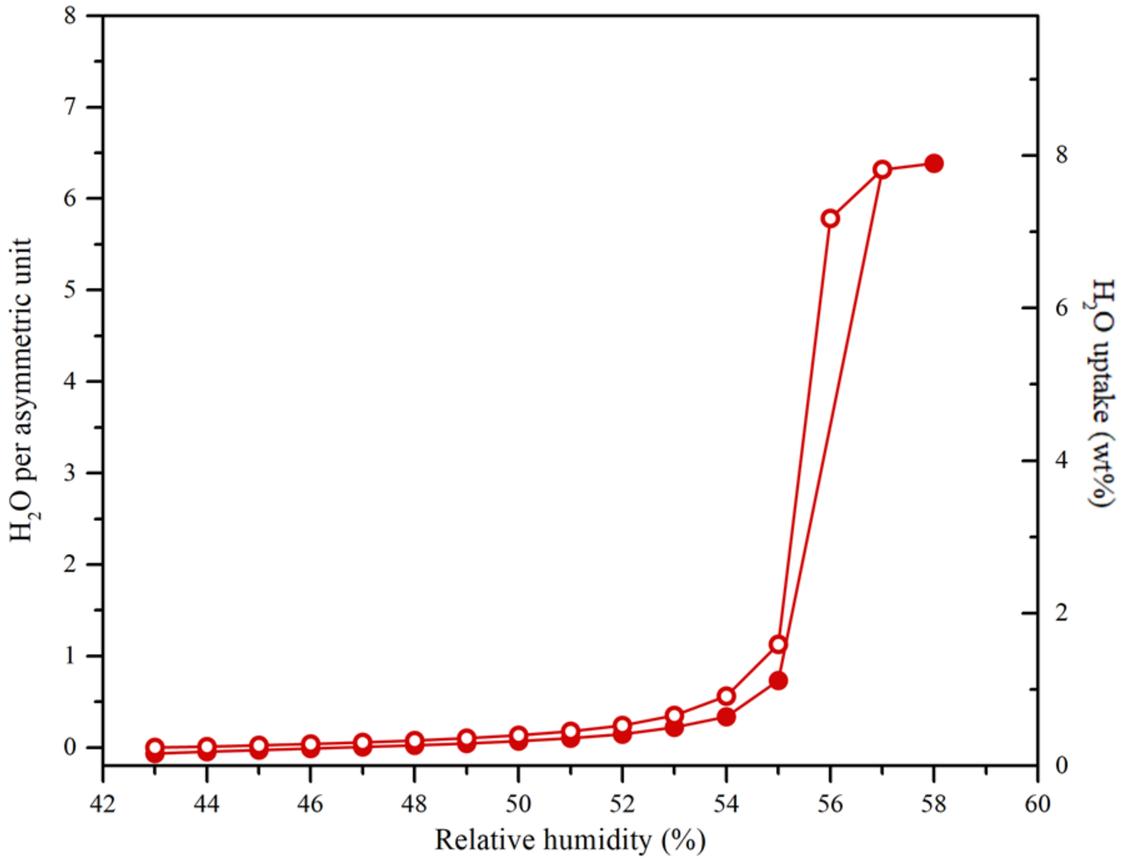

Supplementary Fig. 15. Water vapor adsorption (filled circles) and desorption (open circles) isotherm for **T1** recorded over a narrow RH range at 25 °C using a dynamic vapor sorption analyser.

Supplementary Text 5.3. Estimation of Adsorption Enthalpy

The enthalpy of adsorption ΔH_{ads} (per mole of host) with respect to H_2O loading in **T1** was estimated in the 10 to 40 °C temperature-range using the Clausius-Clapeyron relation

$$\ln P = -\frac{\Delta H_{\mathrm{ads}}}{R}\frac{1}{T} + C$$

where the P is the partial pressure (Pa) of water at temperature T (K), R is the gas constant (8.314 kJ mol^{-1}), and C is a constant[145]. The values of ΔH_{ads} for water adsorption in the low (3 to 55%, Supplementary Fig. 22) and high (58 to 98%, Supplementary Fig. 23) RH ranges were obtained from the isosteres of adsorption (Supplementary Figs. 20 to 21) derived from the first cycle of the three sorption isotherms at 10, 25 and 40 °C (Supplementary Figs. 16 to 19). The value of ΔH_{ads} at zero coverage was estimated from low coverage data (0.01 Wt%) to be $\Delta H_{\mathrm{ads,\,zero}}$ = –44.4(1) kJ mol^{-1}. It should be noted that since there is insufficient separation of the isotherms at low coverage across this temperature–range (Supplementary Fig. 16), the Clausius–Clapeyron relation may give more accurate results at higher loading[146].

It was not possible to calculate ΔH_{ads} in the RH range of the step as there were too few data points in this region of the isotherms. However, even with improved resolution, the water uptake mechanism is not incremental i.e. the crystal converts from **T1–Y** to **T1–R** with a discrete increase in loading. Hence, the absolute value for the enthalpy of adsorption across the RH range of the step $\Delta H_{\mathrm{ads,\,step}}$ can only be approximated by taking the average of the last and first data points from the ΔH_{ads} calculated for the low and high RH regions: these were ΔH_{ads} = –43.6(9) kJ mol^{-1} (0.57 Wt%) and ΔH_{ads} = –47.9(3) kJ mol^{-1} (7.52 Wt%), respectively, yielding $\Delta H_{\mathrm{ads,\,step}}$ = –46 (2) kJ mol^{-1}. It should be noted that the ΔH_{ads} values reported here are only valid across the reported temperature–range in keeping with Kirchhoff's law[145] i.e. ΔH_{ads} should be reported as a function of temperature due to the difference in the partial molar heat capacities of the guest and the host (at constant pressure and composition) at different temperatures.

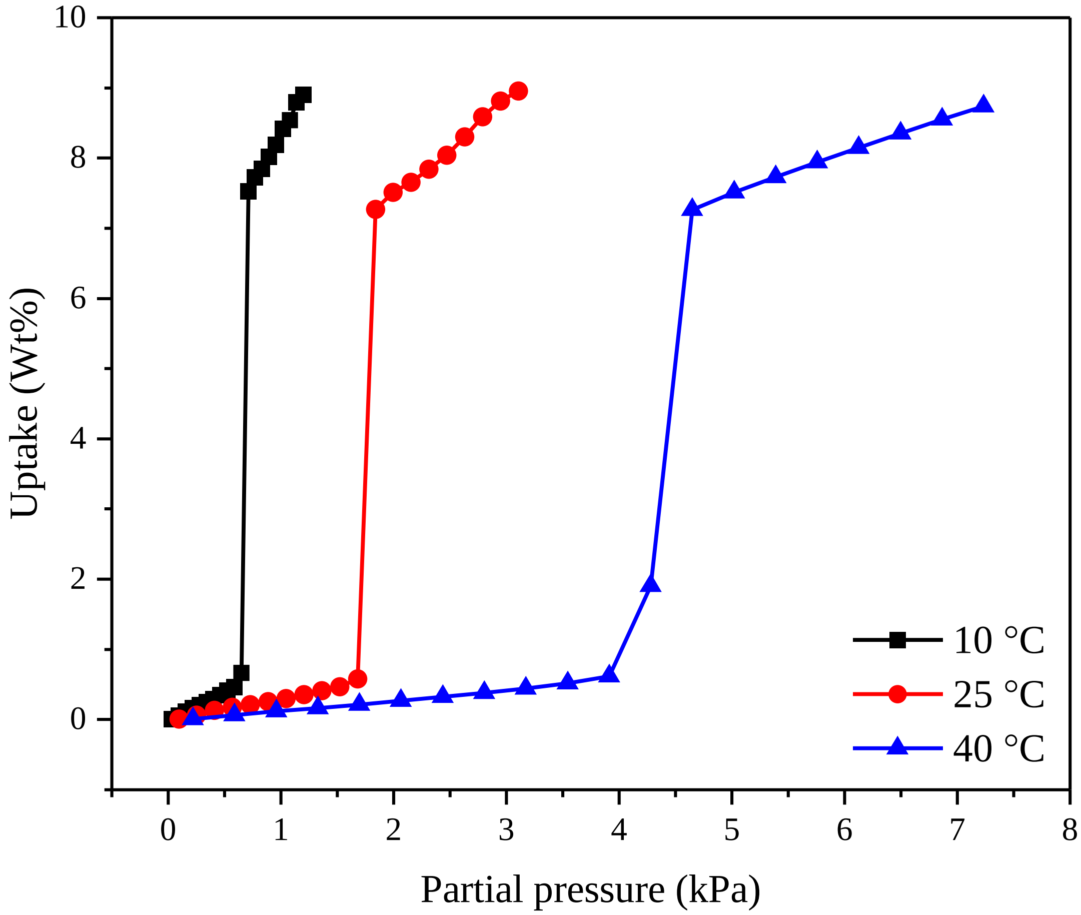

Supplementary Fig. 16. Water vapor adsorption isotherms for **T1** recorded at 10 (black), 25 (red) and 40 °C (blue) using a dynamic vapor sorption analyser.

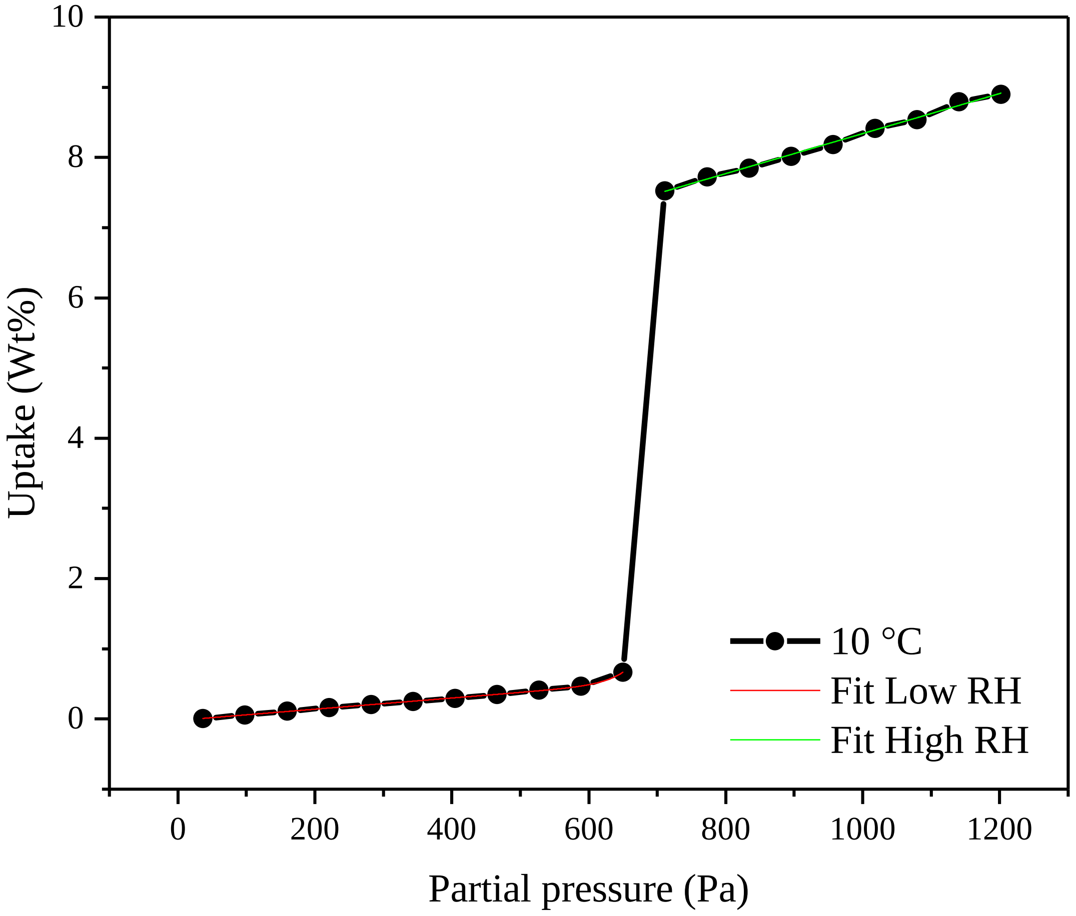

Supplementary Fig. 17. Water vapor adsorption isotherm used for calculating ΔH_{ads} of **T1** recorded at 10 °C using a dynamic vapor sorption analyser. Function overlay for loading at RHs below and above the step are shown in the legend.

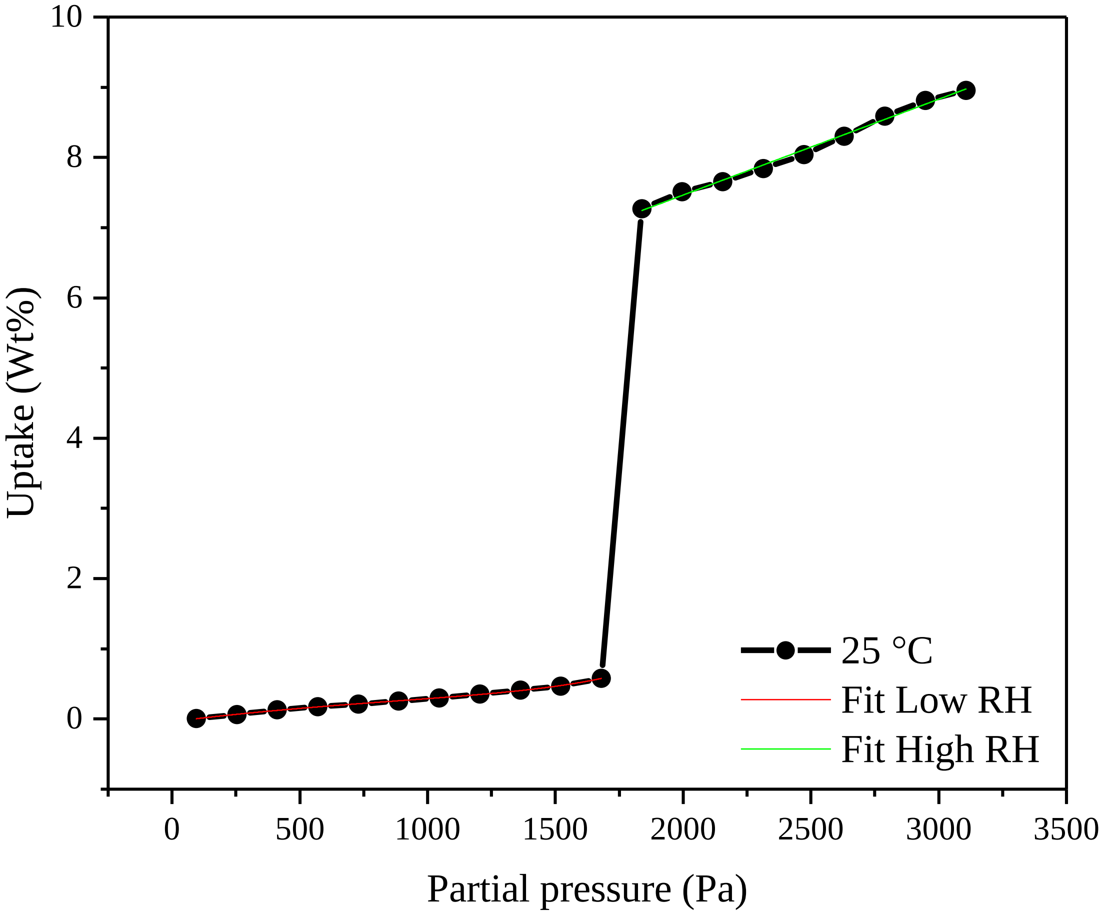

Supplementary Fig. 18. Water vapor adsorption isotherm used for calculating ΔH_{ads} of **T1** recorded at 25 °C using a dynamic vapor sorption analyser. Function overlay for loading at RHs below and above the step are shown in the legend.

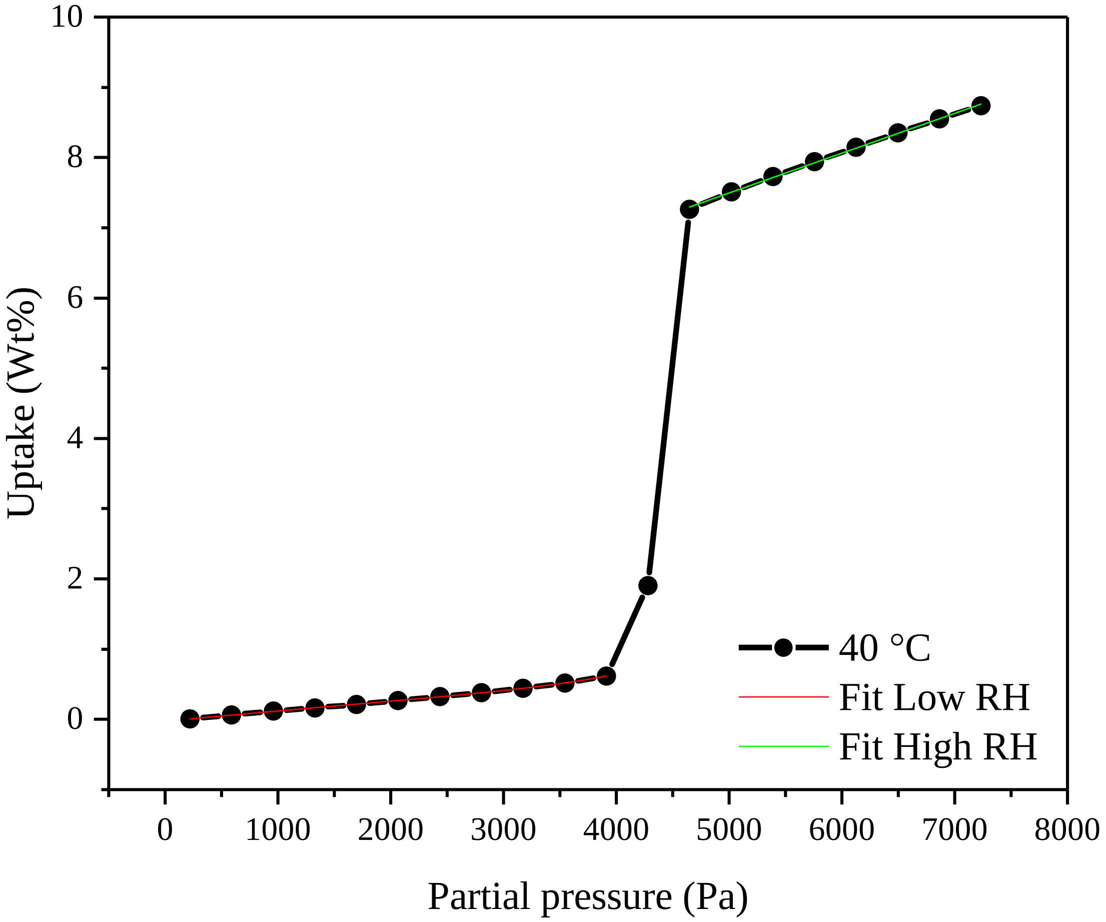

Supplementary Fig. 19 Water vapor adsorption isotherm used for calculating ΔH_{ads} of **T1** recorded at 40 °C using a dynamic vapor sorption analyser. Function overlay for loading at RHs below and above the step are shown in the legend.

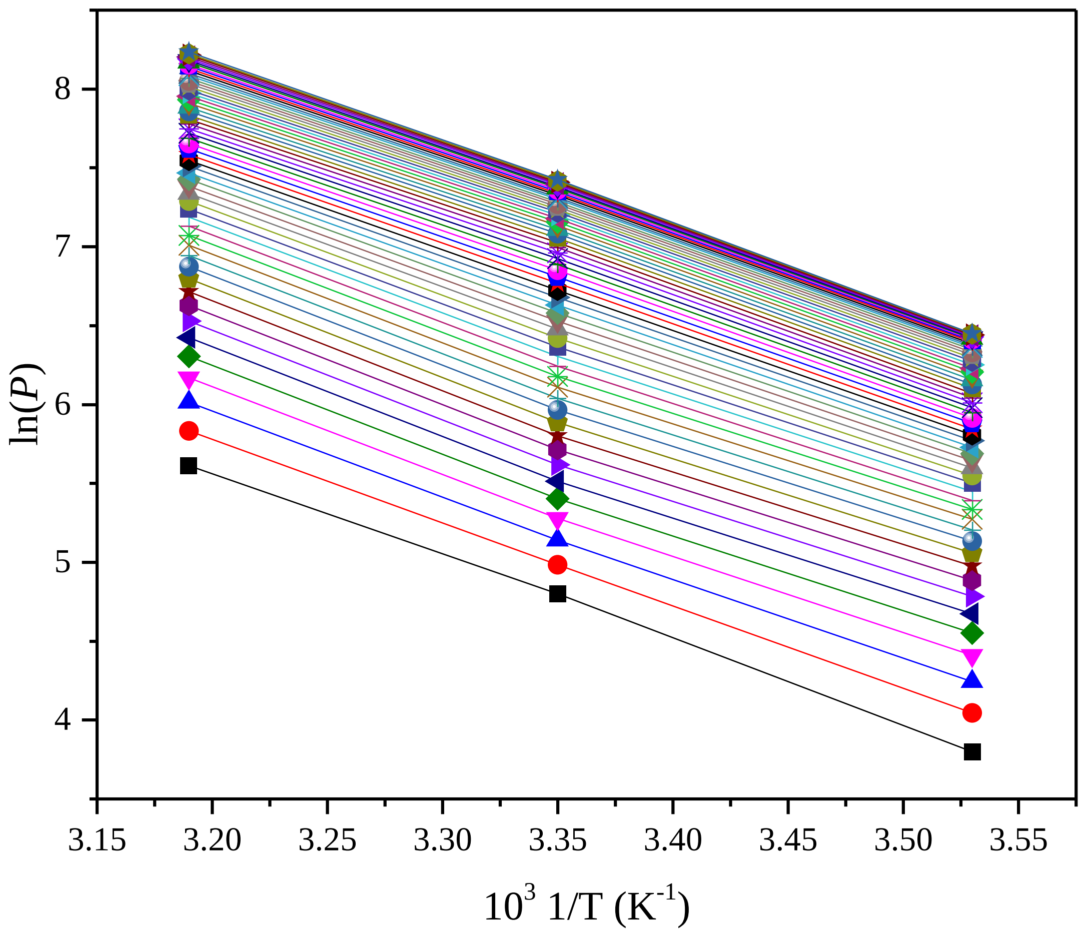

Supplementary Fig. 20 Adsorption isosteres used to calculate ΔH_{ads} over the low RH range. Separate colours and symbols indicated for ln (*P*) and 10^3 1/T at discrete loading values.

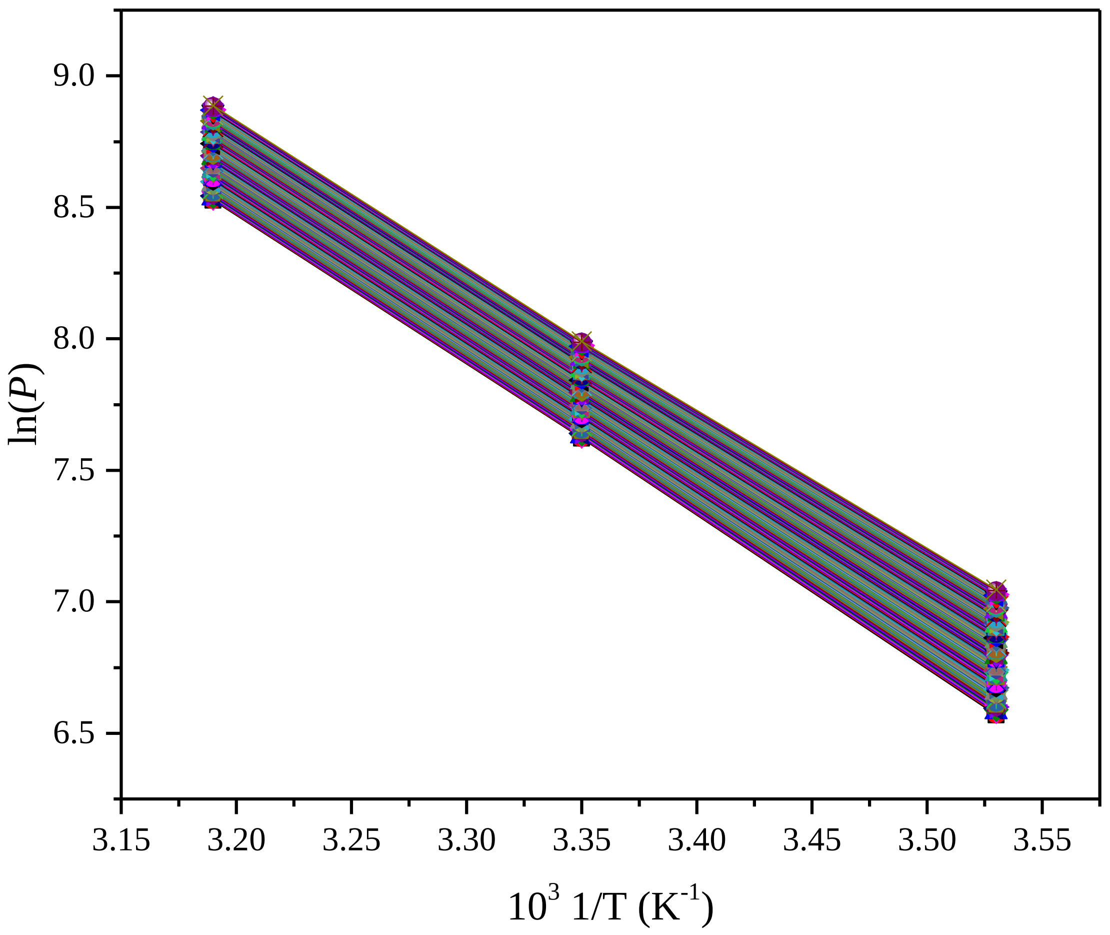

Supplementary Fig. 21 Adsorption isosteres used to calculate ΔH_{ads} over the high RH range. Separate colours and symbols indicated for ln (*P*) and 10^3 1/T at discrete loading values.

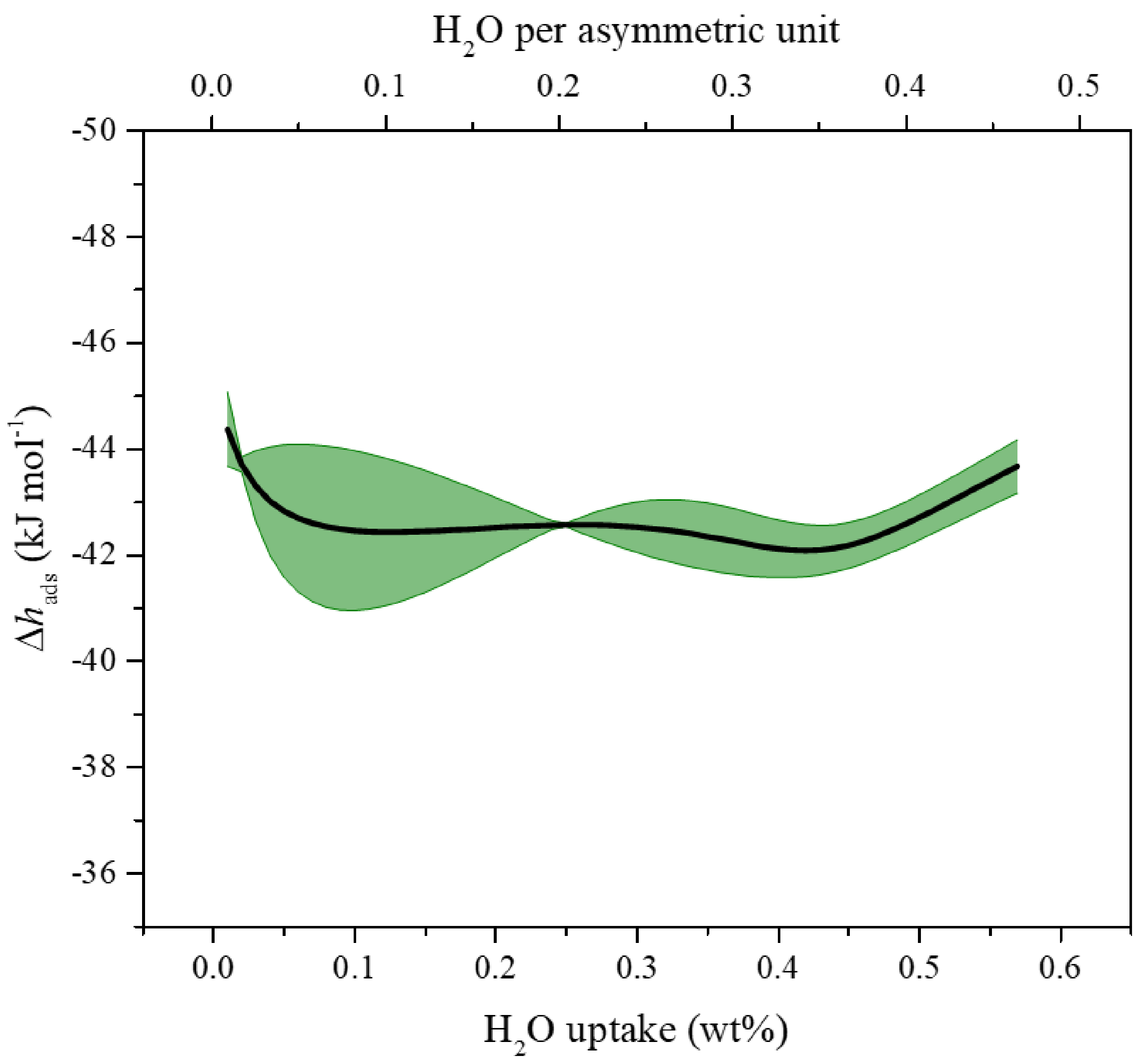

Supplementary Fig. 22 Enthalpy of adsorption ΔH_{ads} with respect to H_2O loading in **T1**. These values were calculated using the Clausius–Clapeyron equation with parameters measured from water vapour sorption isotherms at 10, 25 and 40 °C (Supplementary Figs. 16 to 19) for H_2O coverage in the low RH range. The standard error is shown as the shaded region (green).

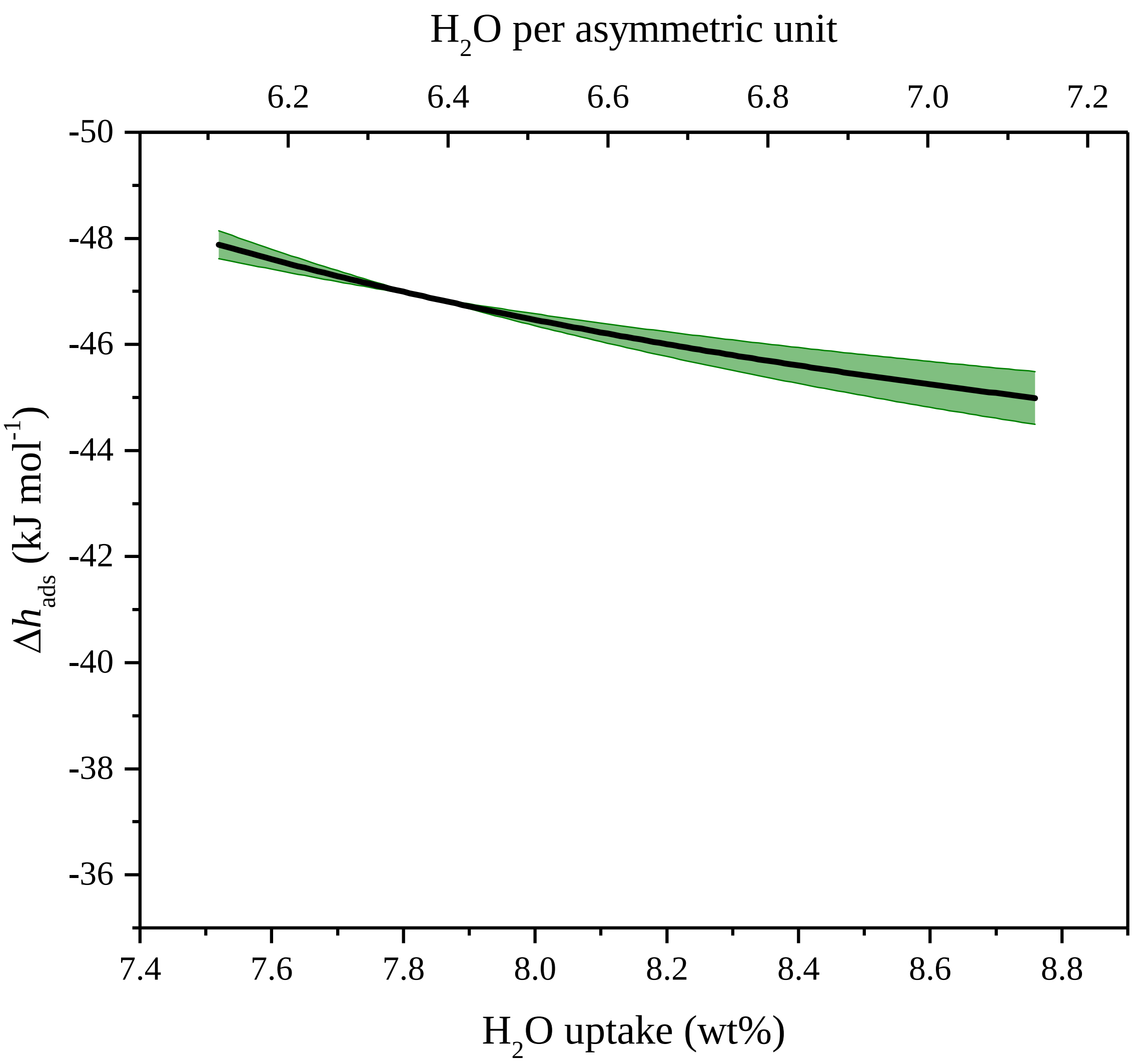

Supplementary Fig. 23 Enthalpy of adsorption ΔH_{ads} with respect to H_2O loading in **T1**. These values were calculated using the Clausius–Clapeyron equation with parameters measured from water vapour sorption isotherms at 10, 25 and 40 °C (Supplementary Figs. 16 to 19) for H_2O coverage in the high RH range. The standard error is shown as the shaded region (green).

Supplementary Text 6. Simulated Water Uptake

Supplementary Text 6.1. Simulated Water Vapor Sorption Isotherms

An adsorption isotherm was simulated by virtually loading water molecules into a single channel of the unit cell. The calculation was carried out at 27 °C for water fugacity values ranging from 2 – 29 kPa in 3 kPa increments (Supplementary Fig. 24). The profile of the simulated sorption isotherm agreed with the experimental results (Fig. 1, Supplementary Figs. 12 to 15), containing a step that occurred at 11 kPa and a maximum loading of 8.5 H_2O molecules per asymmetric unit at 29 kPa. Channel locations that are frequently occupied by water molecules were identified with the aid of a water density distribution map (Supplementary Fig. 25). It was found that water occupied the walls of the channel at first loading and were primarily located at hydroxyl sites O9D and O9B. The dramatic increase in water uptake at higher fugacities resulted in a more evenly distributed network of water throughout the channel.

The energies corresponding to a range of interactions experienced by water molecules were evaluated using energy distribution plots (EDPs) at 5, 8, 11 and 14 kPa (Supplementary Fig. 26). Predominant intermolecular interactions were assigned by comparing the water density distribution contour maps with the EDPs. A single peak at *ca* –6.15 kcal mol^{-1} (–25.7 kJ mol^{-1}) seen at low loadings corresponds to the primary hydrogen bond interactions between water molecules and the hydroxyl groups. The narrow peak profile is indicative of water interacting with the interior of a microporous channel[147]. At the step, an additional peak centred around –15.5 kcal mol^{-1} (–64.8 kJ mol^{-1}) emerged corresponding to the increase in water-water interactions in the channel. The wide distribution of water-water interactions is responsible for the broadness of the peak. The relative frequency of water-water interactions is far higher than water framework interactions, explaining the low intensity peak height at –6.15 kcal mol^{-1} (–25.7 kJ mol^{-1}).

The sorption process was further investigated by performing fixed loading (NVT ensemble) molecular dynamics simulations. It was determined from the simulated sorption isotherms that a single channel in the unit cell contains 25.5 water molecules at full loading. However, since we aimed to model the interactions between included water and the hydroxyl groups O9D, O9B and O9A, fixed loading simulations were limited to between one and six water molecules per channel. Water density distribution plots were used to identify the high probability locations of included water in the channel (Supplementary Fig. 27) and corroborated the findings obtained from the grand conical ensemble calculations: included water tends to occupy the channel walls at first loading and increased loading correspondingly increases the concentration of water in the centre of the channel. From these results, in addition to the DVS analyses, we could conclude that primary water-host interactions at low humidity serve as secondary nucleation sites for incoming water molecules above the RH threshold, resulting in a conceptually infinite network of hydrogen-bonded water molecules.

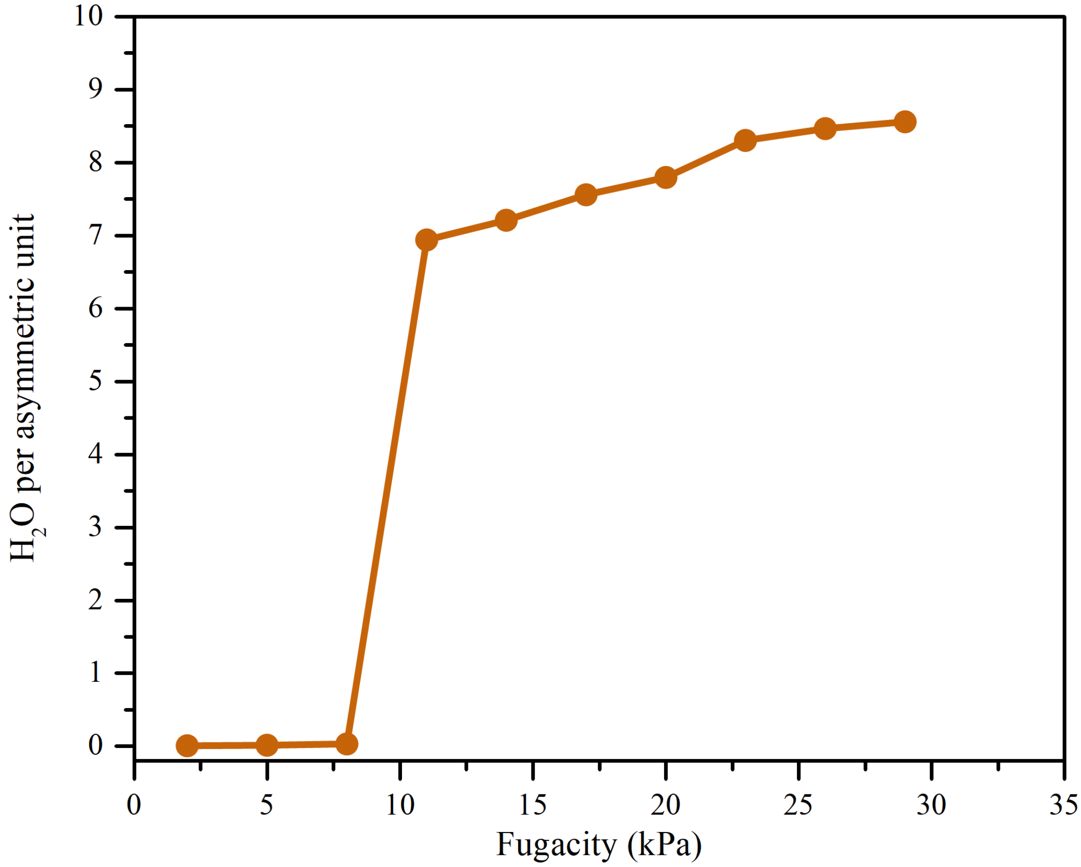

Supplementary Fig. 24. Water sorption isotherm calculated using Grand Canonical Monte Carlo simulation at fixed fugacities within a single channel of the unit cell.

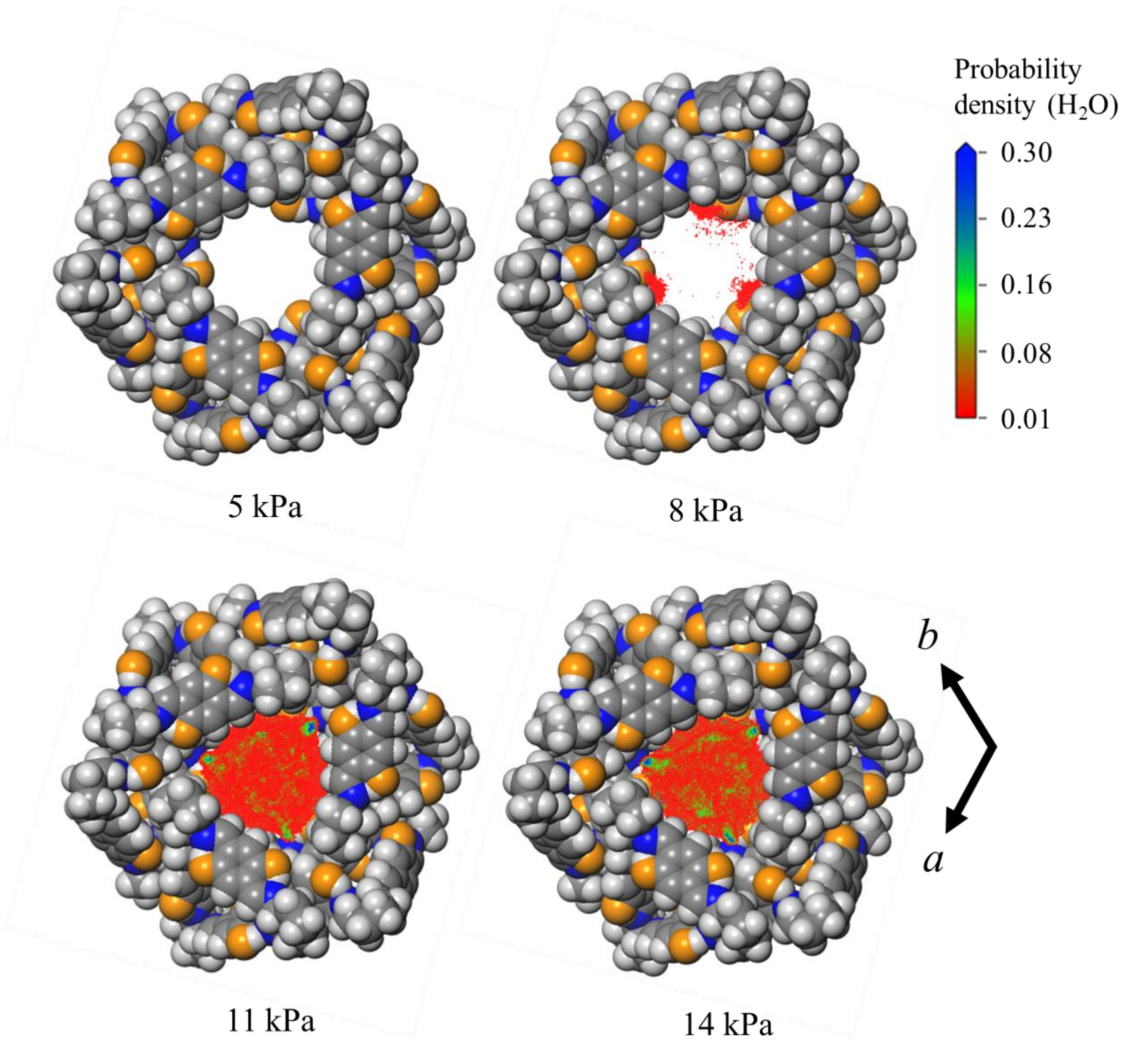

Supplementary Fig. 25. Probability density of included water displayed as a 3D contour map obtained from Grand Canonical Monte Carlo calculations at loadings of 5, 8, 11 and 14 kPa. Colours: colour gradient, probability density of water; grey, carbon; white, hydrogen; blue, nitrogen; orange, oxygen.

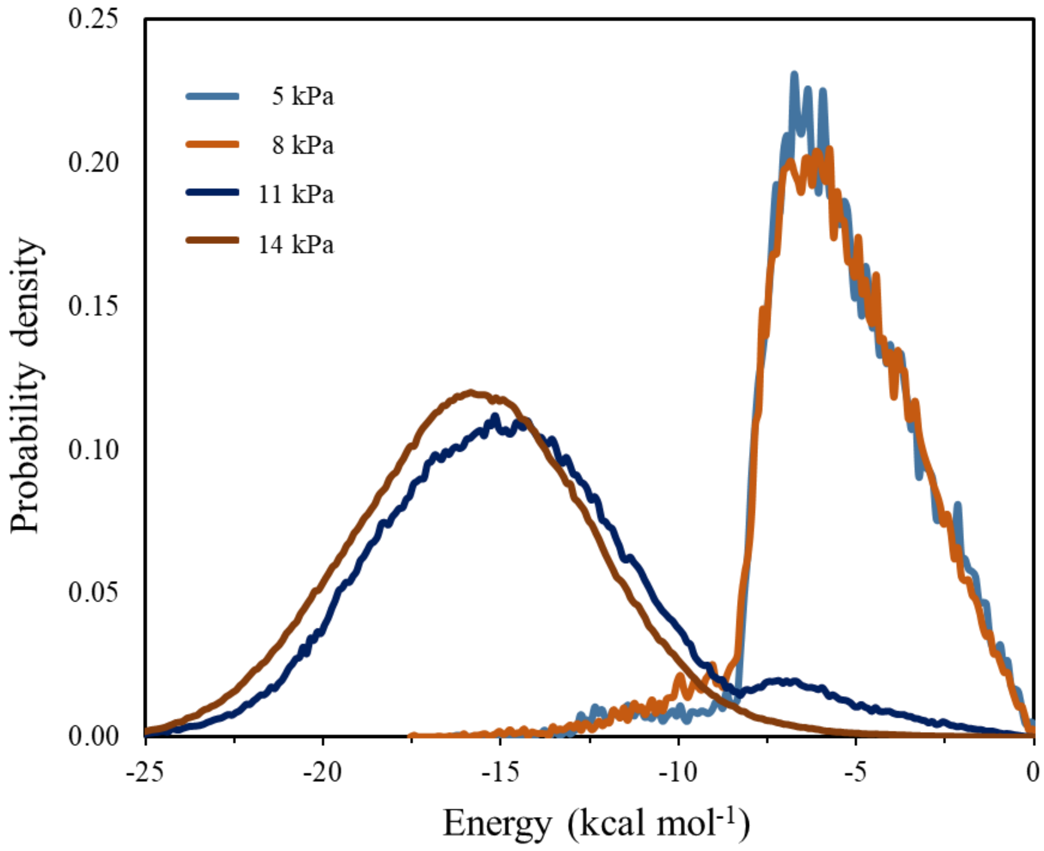

Supplementary Fig. 26. Normalised energy distribution plots generated from Grand Canonical Monte Carlo calculations at loadings of 5, 8, 11 and 14 kPa.

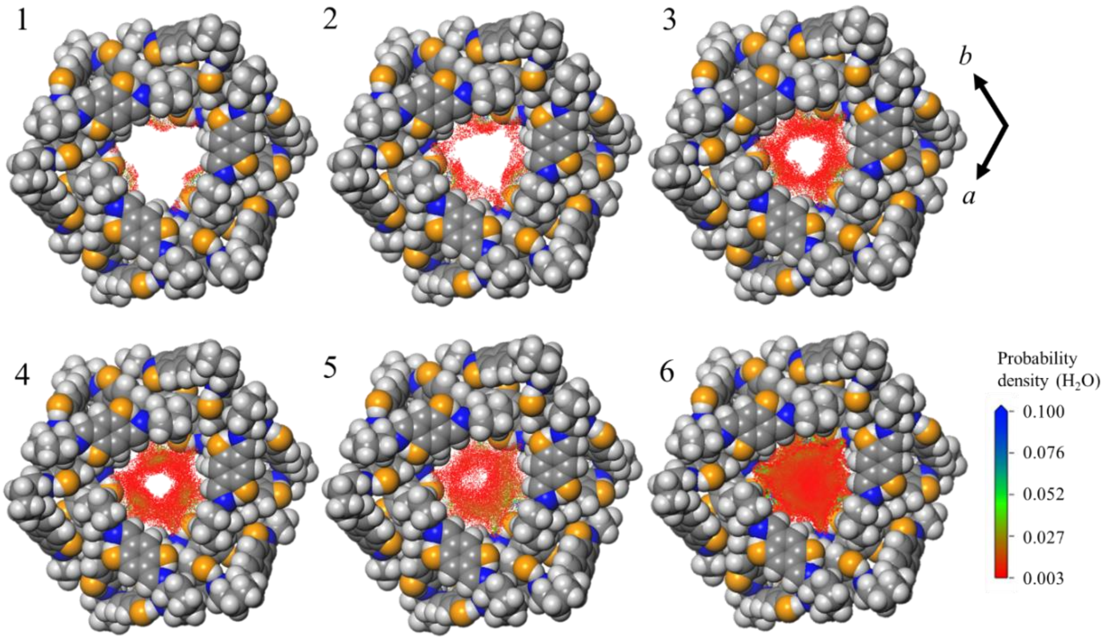

Supplementary Fig. 27. Probability density of included water displayed as a 3D contour map obtained from Canonical Monte Carlo calculations at loadings of 1 to 6 H_2O molecules. Colours: colour gradient, probability density of water; grey, carbon; white, hydrogen; blue, nitrogen; orange, oxygen.

Supplementary Text 6.2. Radial Distribution Function Analysis

To gain insight into the configurational distributions of water in **T1**, the radial distribution functions (RDFs) were calculated for the oxygen-oxygen distances between water and the species in the channel with which it could form hydrogen bonds. The RDF quantifies the probability of finding a chemical species, *a*, at a distance *r* (Å), from a reference species, *b*. The function is often described by the unitless symbol *g* (***r***) and is defined as

$$x_a x_b \rho g_{ab}(\boldsymbol{r}) = \frac{1}{N} \langle \sum_{i=1}^{N_a} \sum_{j=1}^{N_b} \delta(\boldsymbol{r} - \boldsymbol{r_i} + \boldsymbol{r_j}) \rangle$$

where x_i are the mole fractions of atoms *i*, N_i are the number of atoms of type *i*, *N* is the total number of atoms and ρ is the number density determined by the unit cell contents. Equivalent chemical types are excluded (e.g., *i* = *j*). The function tends to unity at sufficiently large *r*. The value of *r* was plotted as a function of *g*(*r*) for all frames (Supplementary Fig. 28). The relative strengths of the intermolecular interactions were inferred from the interatomic distances on the RDF plots; O···O distances shorter than 3.2 Å were considered to be hydrogen-bond interactions[148]. The atomic pairs analysed in this study were those involved in hydrogen-bond interactions between water (W) and the channel hydroxyl sites O9D, O9B and O9A. These include oxygen water to hydroxyl group (O_W···O9x, x = D, B or A) and water-water (O_W···O_W) interatomic distances. The distribution of the distances between H_2O oxygen atoms and the O9D oxygen (O_W···O9D) exhibited a single, narrow, high-intensity peak around 2.9 Å. The narrow distribution of these peaks also suggests that the modal distance is consistent across all frames. The frequency with

which these interactions are observed, and hence the probability, can be inferred from the peak intensity, which increased with loading. A broader distribution of interatomic distances was seen for O_W···O9B and O_W···O9A ranging between 3.0 – 3.7 Å and 2.8 – 3.2 Å, respectively. The modal values of the O_W···O9B and O_W···O9A distances are 3.2 Å and 3.0 Å, respectively, suggesting that these hydrogen bonds will be weaker than those involving O9D. The likelihood of water-water interactions occurring at high loadings is confirmed by the O_W···O_W RDF plot, as the peaks are two orders of magnitude larger than those for interactions with O9D and become more prevalent with an increase in loading. Even though the frequency of water-framework interactions increases overall, water-water interactions are more prevalent for higher loadings. A modal O_W···O_W distance of 2.8 Å suggests that water forms stronger hydrogen bonds with other water molecules than with the framework. The large number of water-water interactions at high loadings suggest that the formation of chains of water molecules extending along the channels and connecting the hydroxyl sites may play a role in the rapid sorption of water beyond 55% RH.

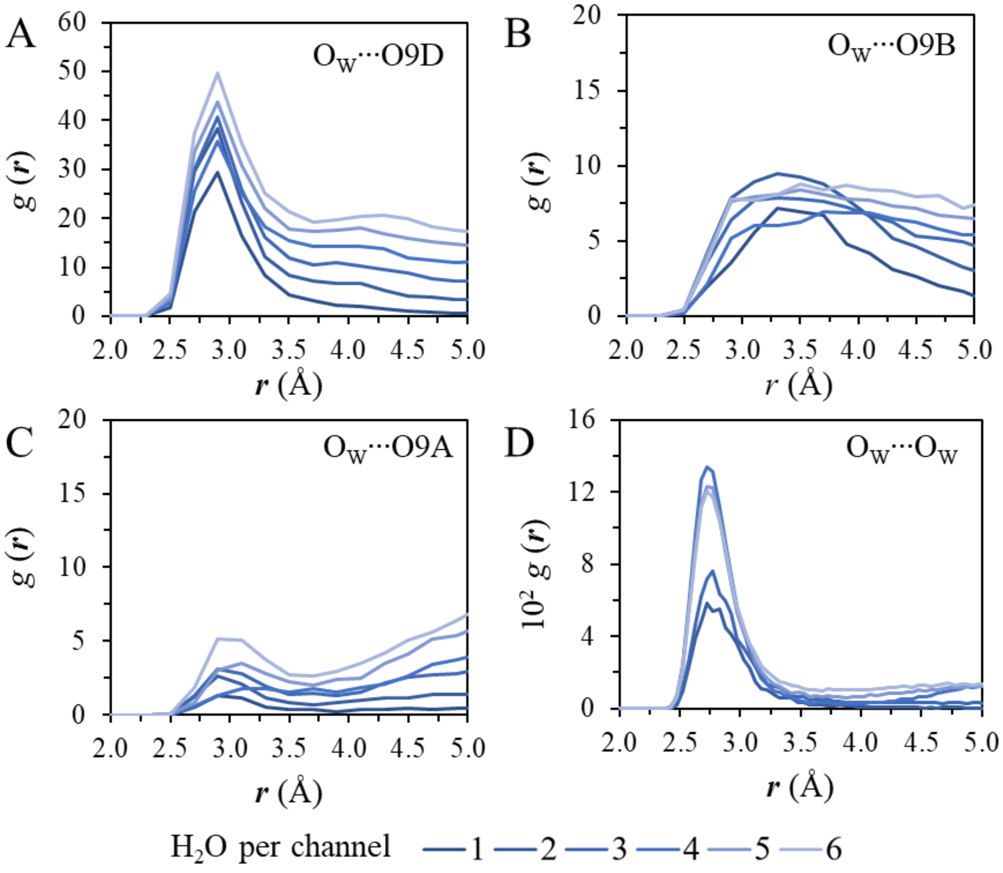

Supplementary Fig. 28. Radial distribution plots of the O_W···O9D (A), O_W···O9B (B), O_W···O9A (C) and O_W···O_W (D) interatomic distances determined from Canonical Monte Carlo calculations for loadings of 1 to 6 H_2O molecules per channel per unit cell.

Supplementary Text 6.3. Mechanism of Water Uptake in **T1**

Monte Carlo simulations at different loadings confirm that high probability locations of the water molecules at low loadings are solely along the channel walls. Higher loading increases the concentration of water in the centre of the channel and radial distribution function analysis further supports these findings. Below 55% RH the water molecules are attracted to the exposed host OH groups, gradually creating a more uniformly hydrophilic channel[149]. These water molecules then serve as secondary nucleation sites for incoming water molecules above 55% RH, resulting in the formation of a conceptually infinite but fluid network of hydrogen-bonded water molecules. The large number of water-water interactions at high loadings suggest that 'flickering' water clusters and chains that connect the hydroxyl sites may play a role in the rapid sorption of water beyond 55% RH.

Supplementary Text 7. Experimental Investigation of Tautomerism

Previous studies have shown that porous systems decorated with salicylimine moieties undergo hydrochromism due to hydrogen bond donation from included water molecules[25,150]. This mechanism is well understood and one of several established for rapid and reversible vapochromism in response to water[25–27]. Proton transfer does not affect the planarity of the salicylaldimine unit, which is independent of the position of the hydrogen atom along the O···H···N bond. We devised a procedure to record diffraction data for a hydrous crystal (see Supplementary Text 9) with the dual aims of (i) confirming the occurrence of tautomerism and (ii) determining the positions of the water molecules in the channel. In the SCXRD model of **T1-R** at −173 °C, changes in the bond lengths of the channel-exposed enolimine moieties were subtle and suggested only partial enolimine/ketoenamine tautomerism. However, more conclusive evidence of shifts in the tautomeric equilibrium was obtained by means of diffuse reflectance UV-vis spectroscopy (Supplementary Text 7.2).

Supplementary Text 7.1. Comparison of Salicylimine Bond Lengths

Structural changes resulting from tautomerism in molecules containing salicylimine groups have been identified using SCXRD[151]. This involves reduction of the imine to form a secondary amine and oxidation of the hydroxyl group to form a carbonyl group. However, owing to unreliable location of the labile hydrogen atom by means of X-ray diffraction, the major tautomer is usually assigned by monitoring structural changes in the imine C−N and hydroxyl C−O bond distances. The C−O and C−N bond lengths of the channel-exposed salicylimine groups were assessed for yellow and red crystals at −173 and 25 °C (Supplementary Table 4). The geometric parameters of

Supplementary Table 4. Comparison of C−O and C−N bond lengths for salicylimine moieties containing O9D, O9B and O9A in **T1-Y** and **T1-R**.

Temperature (°C)	Bond	OH site	Bond length (Å)		Bond length difference (Å) (**T1-R** – **T1-Y**)
			**T1-Y**	**T1-R**	
−173	C–O	O9D	1.370(3)	1.334(6)	-0.036
		O9B	1.378(3)	1.382(6)	0.004
		O9A	1.373(3)	1.370(5)	-0.003
	C–N	O9D	1.257(8)	1.269(4)	0.012
		O9B	1.267(8)	1.276(6)	0.009
		O9A	1.276(7)	1.275(4)	-0.001
25	C–O	O9D	1.348(6)	1.350(10)	0.002
		O9B	1.356(8)	1.350(10)	-0.006
		O9A	1.359(7)	1.380(10)	0.021
	C–N	O9D	1.270(10)	1.270(20)	0.000
		O9B	1.280(10)	1.280(20)	0.000
		O9A	1.280(10)	1.280(20)	0.000

the most exposed OH group, O9D, differed most between the structures determined at both temperatures; at −173 °C the C−O bond length of the red crystal decreased relative to that of the yellow crystal by 0.036 Å, while the C−N bond length increased by 0.012 Å. However, these changes are relatively small compared to the enolimine and ketoenamine bond lengths retrieved from the CSD. Distributions of published C−O, C−C and C−N bond lengths of enolimine and ketoenamine tautomers are shown in Supplementary Figs. 29 to 31. The active site bond lengths in the yellow crystals correspond to enolimine bond lengths reported in the literature. Conversely, with the exception of the C−C bond (Supplementary Fig. 30), the bond lengths of the red crystals do not fall within the distribution of the ketoenamine tautomers. It should be noted that the crystal structure yields a weighted average of the C−O and C−N bond lengths, affording a structural model that is a superposition of enolimine and ketoenamine forms[152]. The absence of changes in these bond lengths is consistent with previously reported X-ray structural studies of similar transitions[153–155].

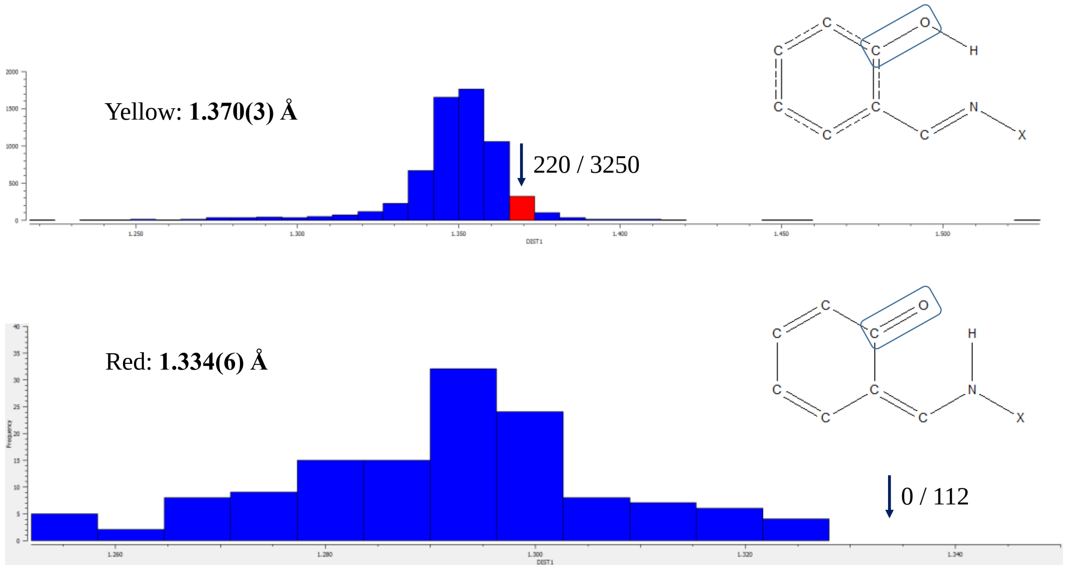

Supplementary Fig. 29. The distribution of C–O bond lengths of a fragment derived from the salicylimine and salicylamine moieties in the Cambridge Structural Database. The bond lengths measured for **T1-Y** (top) and **T1-R** (bottom) are given in boldface. Arrows indicate the position of the bond lengths of the salicylimine unit containing O9D determined for **T1** with respect to the lengths from the CSD. Bond lengths of **T1** that fall within the range of a bin are coloured red and the relative fraction of that bin with respect to the entire range is given.

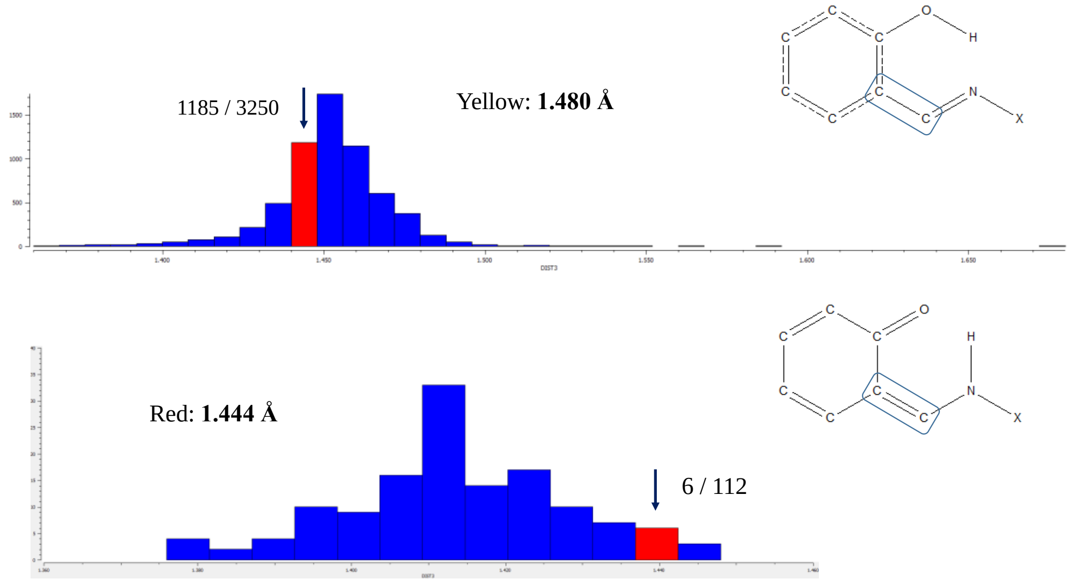

Supplementary Fig. 30. The distribution of C–C bond lengths of a fragment derived from the salicylimine and salicylamine moieties found in the Cambridge Structural Database. The bond lengths measured for **T1-Y** (top) and **T1-R** (bottom) are given in boldface. Arrows indicate the position of the bond lengths of the salicylimine unit containing O9D determined for **T1** with respect to the lengths from the CSD. Bond lengths of **T1** that fall within the range of a bin are coloured red and the relative fraction of that bin with respect to the entire range is given.

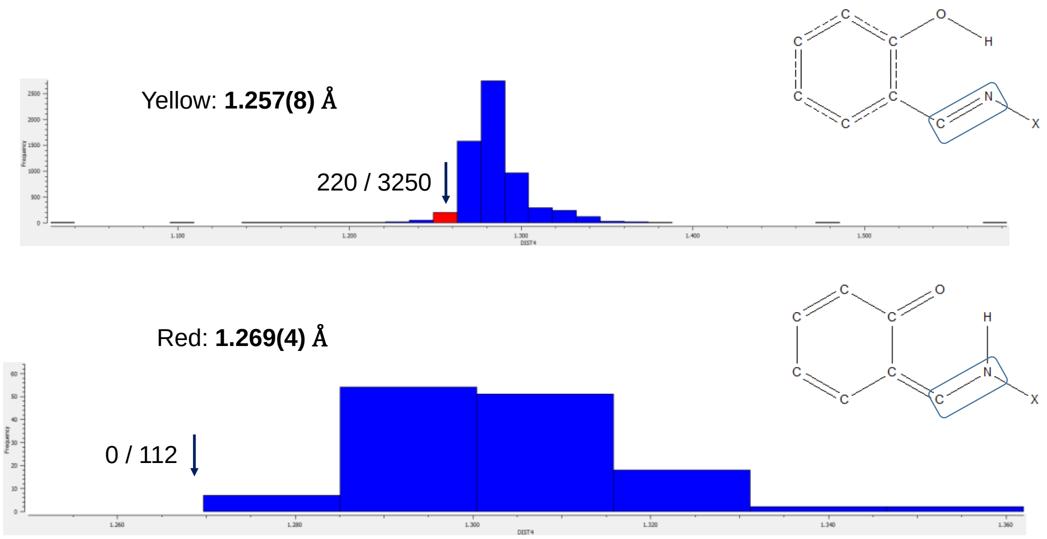

Supplementary Fig. 31. The distribution of C–N bond lengths of a fragment derived from the salicylimine and salicylamine moieties found in the Cambridge Structural Database. The bond lengths measured for **T1-Y** (top) and **T1-R** (bottom) are given in boldface. Arrows indicate the position of the bond lengths of the salicylimine unit containing O9D determined for **T1** with respect to the lengths from the CSD. Bond lengths of **T1** that fall within the range of a bin are coloured red and the relative fraction of that bin with respect to the entire range is given.

Supplementary Text 7.2. UV-vis Spectroscopy

UV-vis spectroscopy is routinely employed to monitor tautomeric equilibrium in salicylimine-containing groups[25,156–159]. The diffuse reflectance UV-vis spectrum was recorded for a powdered sample of **T1-Y**. Upon hydration of the sample, the crystals changed colour to red and a new peak emerged at λ_{max} = 530 nm (Supplementary Fig. 32). The contribution to the spectrum due to hydration can be evaluated by subtracting the spectrum of the anhydrous sample from that of the hydrous sample (Supplementary Fig. 32, inset). Further insights into the emergence of this peak were provided by density functional theory (DFT) calculations, as described in Supplementary Text 8.

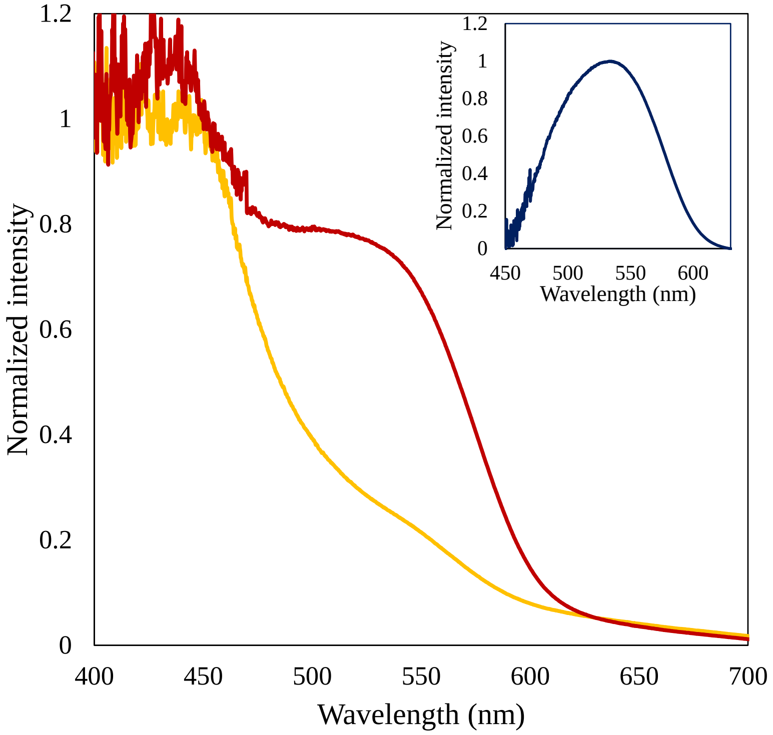

Supplementary Fig. 32. Solid state UV-vis spectra at 0% and 90% RH, recorded at 25 °C. Inset: difference spectrum (i.e., spectrum at 0% RH subtracted from the spectrum at 90% RH).

Supplementary Text 7.3. Solid-State Nuclear Magnetic Resonance Spectroscopy

Direct-polarisation (DP) and cross-polarisation (CP) magic angle spinning (MAS) solid-state NMR spectroscopy (SS-NMR) was carried out at 25 °C on **T1-Y** and **T1-R** using a 500 MHz Varian VNMRS with a 4 mm HX magic angle spinning (MAS) probe (spinning range 1kHz to 18 kHz), managed by the Central Analytical Facility of Stellenbosch University. ^{1}H SS-NMR was not carried out because MAS spinning frequencies greater than 30 kHz are required to yield meaningful results[160]. Furthermore, hydrating **T1-Y** would likely result in strongly overlapping peaks that would be difficult to deconvolute in the ^{1}H spectrum[161]. We expected to obtain more meaningful results for ^{13}C (Supplementary Fig. 33), ^{14}N and ^{15}N nuclei. However, only the ^{13}C NMR spectra (Supplementary Fig. 34) yielded discernible peaks; ^{14}N and ^{15}N NMR did not yield sufficient resolution for peak identification for either form**.** No difference in the ^{13}C spectra of **T1-Y** and **T1-R** could be discerned with confidence (Supplementary Table 5).

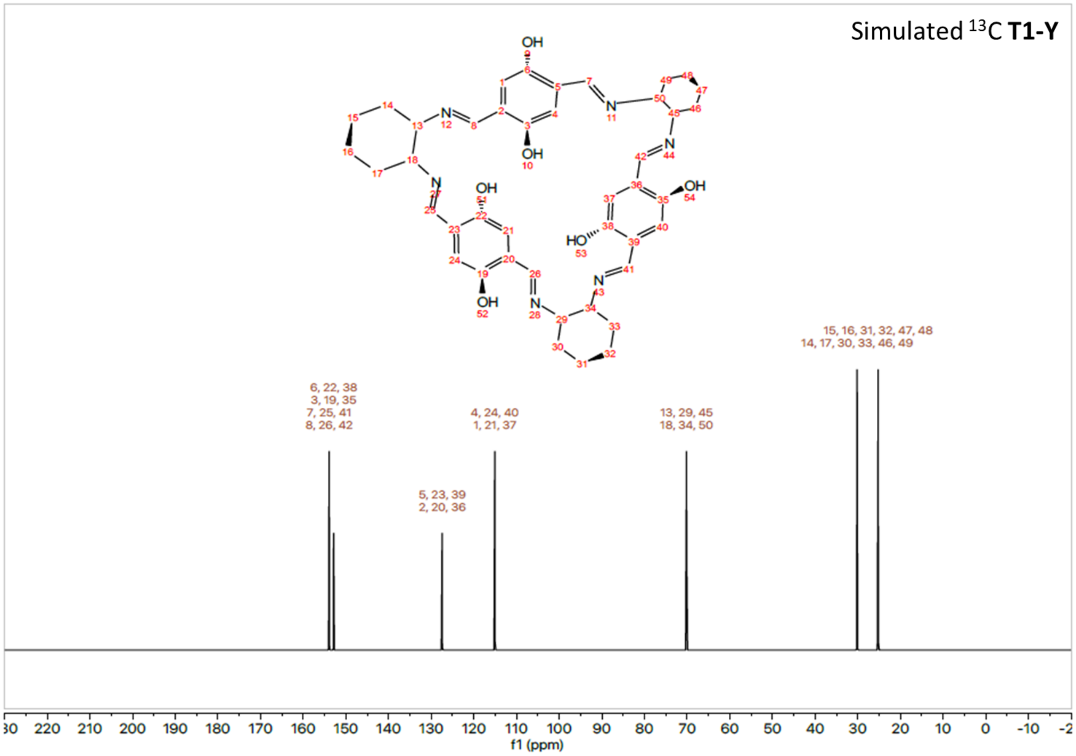

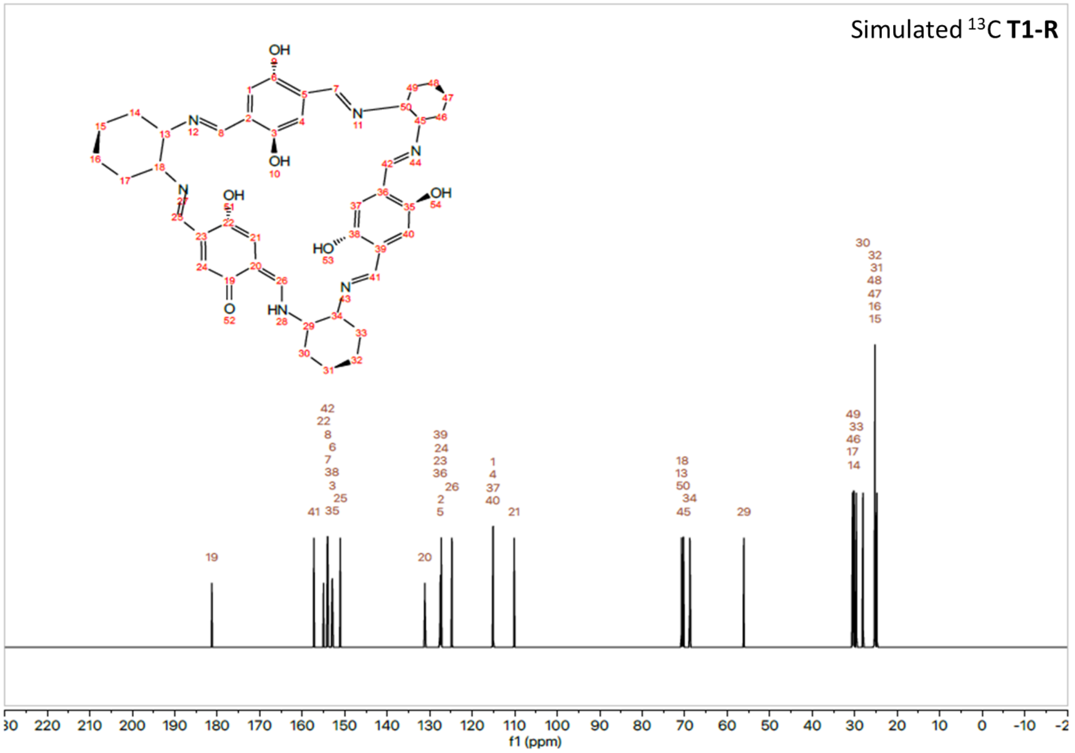

Supplementary Fig. 33. ^{13}C chemical shifts of the enolimine (top) and ketenamine (bottom) tautomers of **T1** were simulated using *Mestrelab Research Ensemble NMR Prediction*. Since water cannot access all the hydroxyl groups some will remain in the enolimine form. Therefore, a spectrum of **T1-R** was simulated using a combination of the enolimine and ketoenamine tautomers.

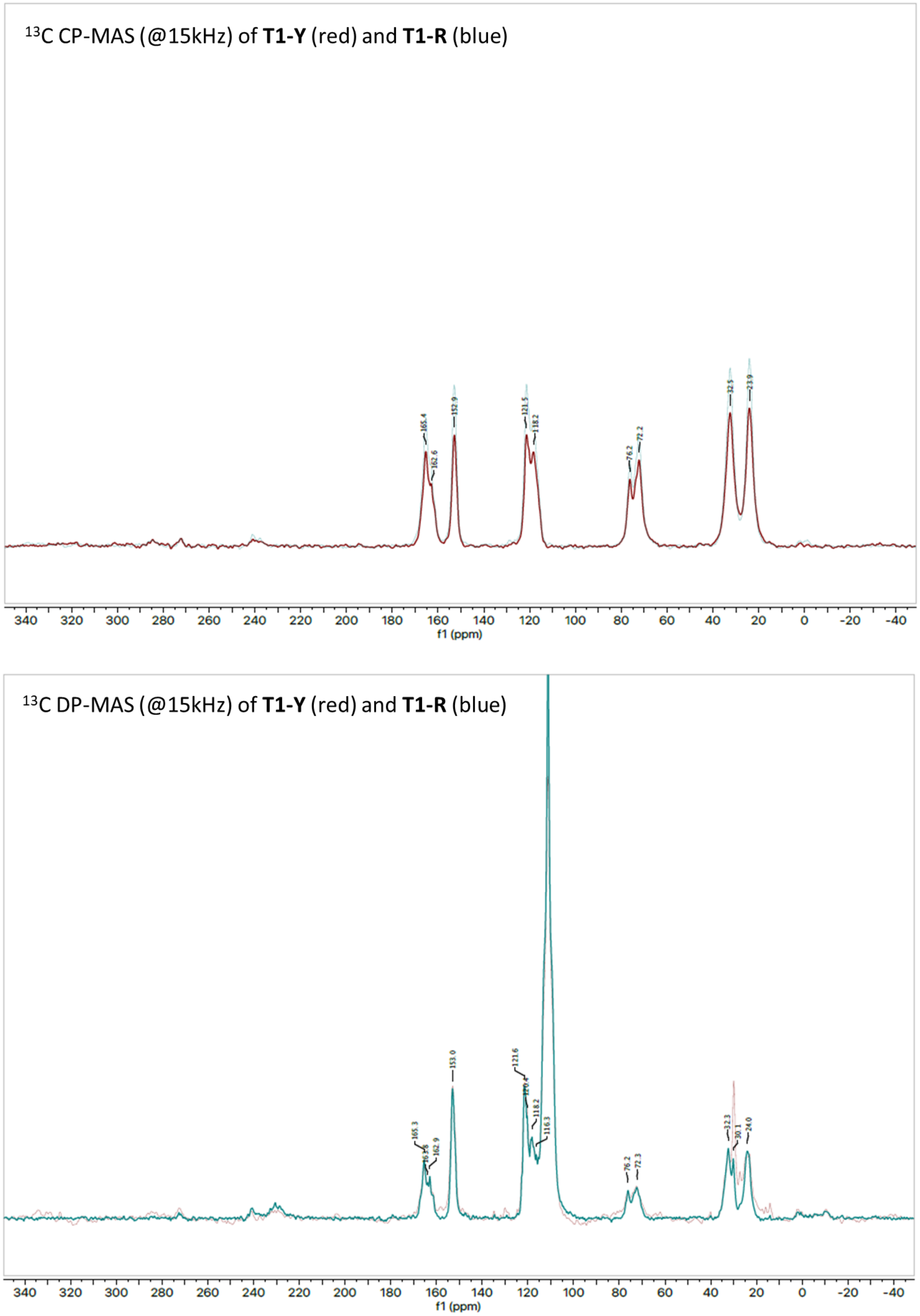

Supplementary Fig. 34. ^{13}C CP-MAS (top) and DP-MAS (bottom) SS-NMR carried out on **T1-Y** (red) and **T1-R** (blue).

Supplementary Table 5. Experimental, simulated and solution-state chemical shift tensors (ppm) for carbon atoms in **T1** determined by DP- and CP-MAS-SS-NMR. Atom numbers correspond to Supplementary Fig. 2. See note*.

Atom number	Experimental (both spectra)		Simulated		Solution NMR
	DP	CP	**T1-Y**	**T1-R**	**T1-Y**
2, 11	165.3<br>**163.8**<br>162.9	165.4<br><br>162.6	153.9	151.0-157.3	163.92
5, 8	**153.0**	**152.9**	152.9	152.9-155	152.9
3,6	**121.6**<br>120.4	**121.5**	127.4	127.4-127.6	121
4,7	**118.2**<br>116.3<br>111.2	**118.2**	115.1	110.1-127.3	118.37
13, 18	76.2<br>**72.3**	76.2<br>**72.2**	70.1	68.8 - 70.8	73.82
14, 17	**32.3**<br>30.1	**32.5**	30.1	28.1-30.5	32.95
15, 16	**24.0**	**23.9**	25.2	24.8-25.3	24.17

*Peaks with a sufficient signal-to-noise ratio were selected and their chemical shifts are reported in column 2. These correspond to the solution-state NMR spectrum of **T1**. Fewer peaks are expected for **T1-Y** as all chromophores are present in the enolimine form, retaining the C_3 symmetry with seven distinct chemical environments for carbon[20]. In contrast to **T1-Y**, more peaks are expected in the spectra of **T1-R** due to a reduction in symmetry to C_1, as a mixture of the ketoenamine and enolimine form are present.

Supplementary Text 7.4. Attenuated Total Reflectance Fourier Transform Infrared Spectroscopy

ATR-FTIR spectra were measured at 0% and 90% RH and additional spectrum of **T1** in a drop of liquid H_2O (Supplementary Fig. 35). The sharp peak at 1623 cm^{-1} in the spectrum of a dry crystal corresponds to a C=N stretching frequency[162]. The O-H stretching frequencies appear as a raised background between 2700 cm^{-1} and 3100 cm^{-1}. Exposing **T1** to water vapour resulted in additional broad peaks at 3456 cm^{-1} and 3259 cm^{-1} and a low intensity peak at 2358 cm^{-1}. The position and shape of the peaks correspond to those observed for a hydrogen-bonded network of nanoconfined water[163]. It is worth noting that exposing the sample to liquid water results in a red shift of the peak at 3428 cm^{-1} to 3378 cm^{-1}. This corresponds to the stronger intermolecular hydrogen-bonded network in liquid water, resulting in less molecular vibrational energy and thus a lower frequency. The absence of a peak at 3378 cm^{-1} also suggests the absence of liquid surface water at 90% RH. Computational vibrational frequency analysis of the model compound confirms that increasing the number of H_2O molecules that can interact with a hydroxyl group favours the

formation of the ketoenamine form and indicates that the peak present in the hydrated form at 2358 cm^{-1} corresponds to a frequency mode of trifurcated water.

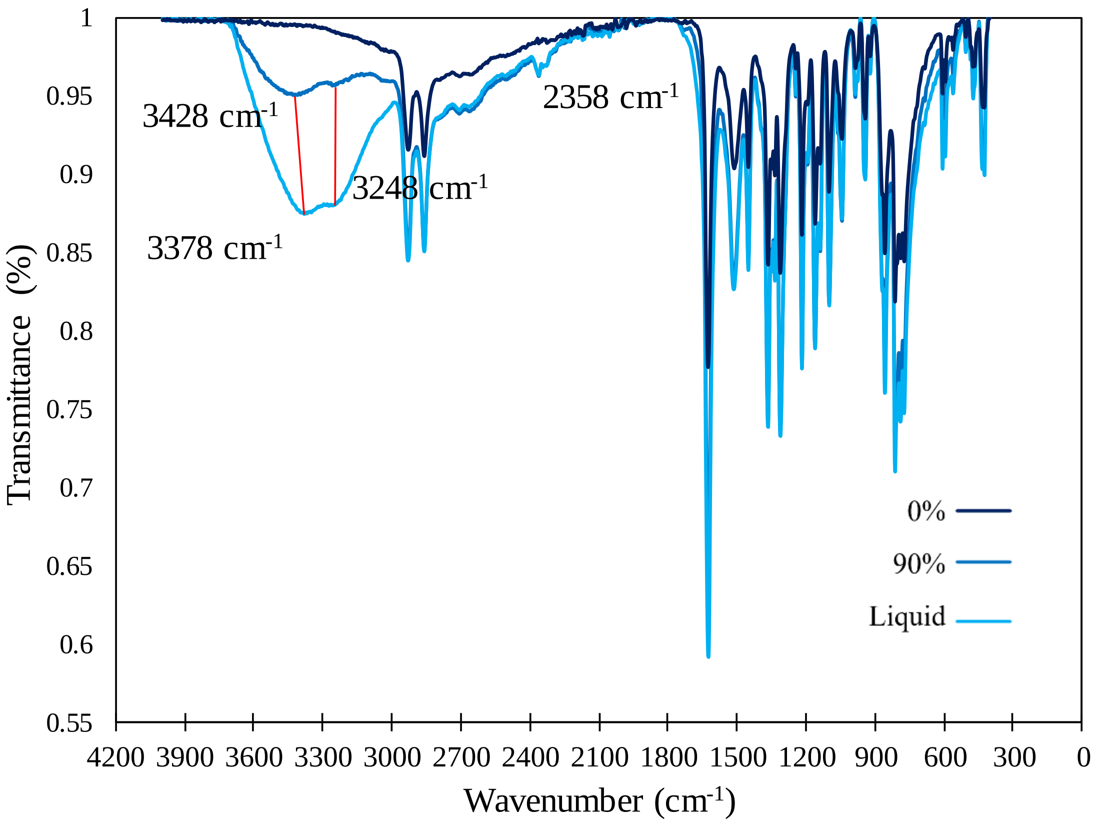

Supplementary Fig. 35. ATR-FTIR spectrum of **T1** at 0% and 90% RH. An additional spectrum was recorded for **T1** in a drop of liquid H_2O. Important frequencies annotated. Red-shift in water peak shown with a red line.

Supplementary Text 8. Computational Study of Tautomerism

Supplementary Text 8.1. Density Functional Theory Orbital Calculation

Calculations to identify the energy conversions between the enolimine and ketoenamine tautomers were performed using density functional theory (DFT) at the B3LYP-D3/6-311G level of theory. The location of the orbitals that contribute to peaks observed in the UV-Vis spectrum was based on the atomic coordinates of the formula unit obtained from high quality crystallographic data. The excitations used to generate the profile of the UV-Vis spectrum take place between pairs of occupied (particularly HOMO down to HOMO-5) and unoccupied (LUMO up to LUMO+5) molecular orbitals. These twelve molecular orbitals (Supplementary Fig. 36) were used to confirm that the salicylimide group is the chromophore of the system and showed that the chromophore is virtually independent of neighbouring groups; i.e. each pair of occupied and unoccupied orbitals responsible for a signal in the UV-Vis spectrum is located on the same phenyl ring with a negligible amount of overlap with other groups.

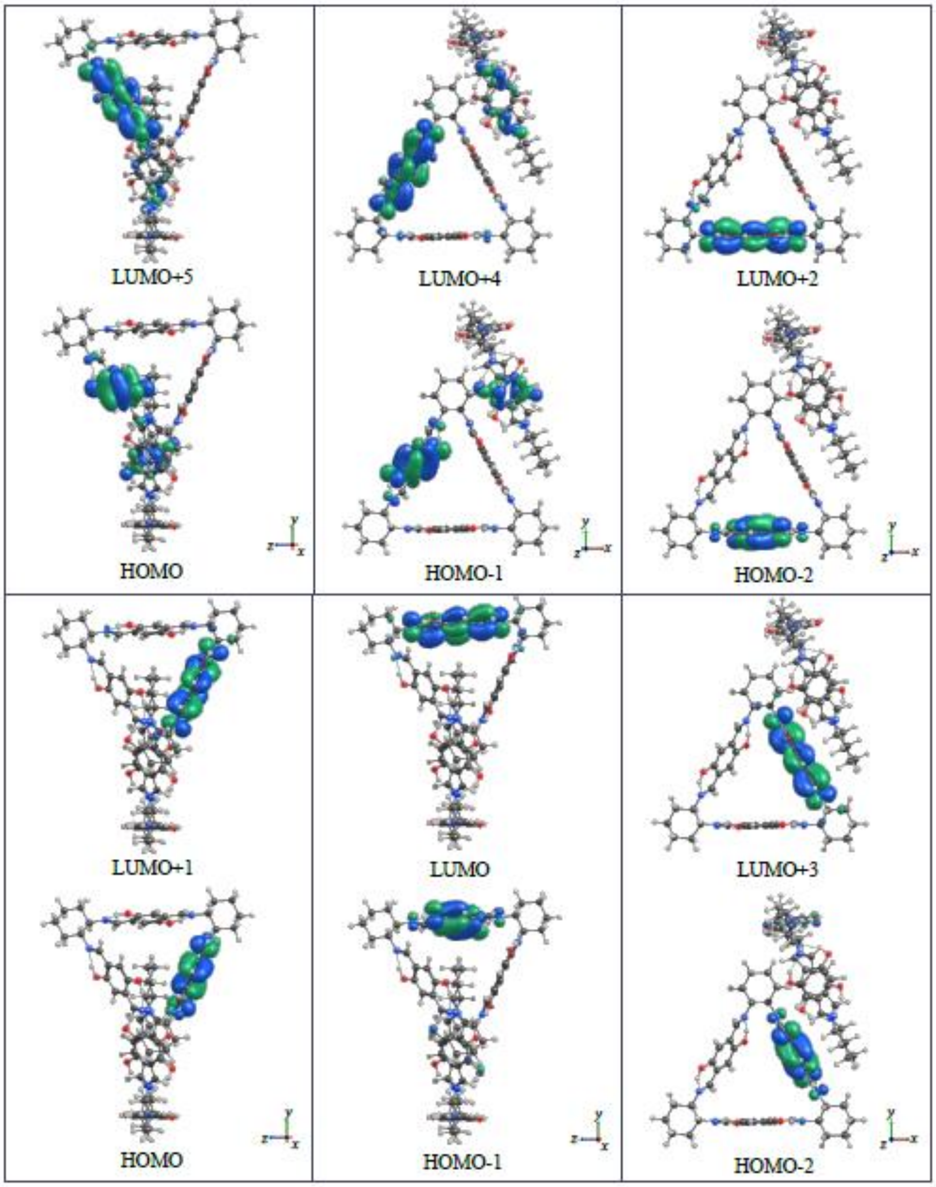

Supplementary Fig. 36. The six highest occupied molecular orbitals and six lowest unoccupied molecular orbitals are displayed (isovalue set to 0.03) on the asymmetric unit and in pairs of occupied and unoccupied orbitals.

Since the orbitals responsible for the colour change are limited to the salicylimine groups, the molecule 2,5-bis(E-(cyclohexylimino)methyl)benzene-1,4-diol (**CID**, Supplementary Fig. 37) was used as a model compound for T1. Reducing T1 to this compound allows for the calculation of energy differences and the modeling of interactions with water at a high level of theory to explain the yellow-to-red colour conversion.

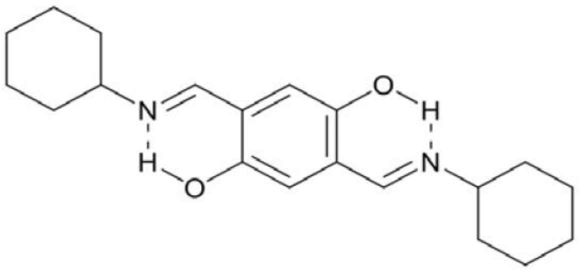

Supplementary Fig. 37. 2,5-bis(E-(cyclohexylimino)methyl)benzene-1,4-diol, the model compound **CID** used in DFT calculations.

The HOMO/LUMO excitation energies of the enolimine (Supplementary Fig. 38A) and ketoenamine (Supplementary Fig. 38B) forms of **CID** were calculated using the 6-311++G(d,p) basis set in combination with a range of methods, namely B3LYP-D3, B97D, B98, CAM-B3LYP-D3, M06-D3, PBEPBE-D3 and ωB97XD (Supplementary Table 6). The calculations suggest that a peak should be observed between 356 and 495 nm in the spectrum of a yellow crystal. Since only ¼ of the hydroxyl groups are accessible to the channel, it stands to reason that only a fraction of the salicylimine groups could undergo tautomerism. Therefore, any peaks present in the spectrum of yellow crystals should also appear in the spectrum of red crystals, with their magnitudes attenuated. The B3LYP-D3 method yielded a wavelength of 516 nm which correlates with the peak (λ_{max} = 530 nm) observed in the experimental spectrum of the hydrous crystals, and was hence used for further calculations. We infer from these results that the spectrum of yellow crystals should contain a peak with λ_{max} = 405 nm (Supplementary Fig. 39).

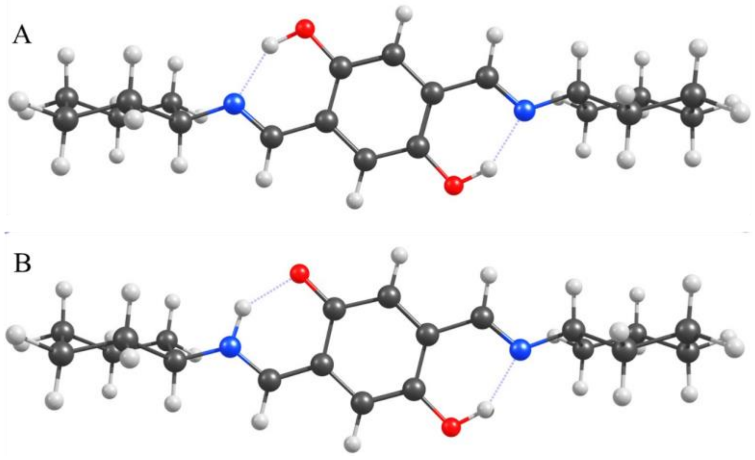

Supplementary Fig. 38. Enolimine (A) and ketoenamine (B) tautomers of **CID**. Colours: grey, carbon; white, hydrogen; blue, nitrogen; red, oxygen.

Supplementary Table 6. HOMO-LUMO transitions in wavelength (nm) for the enolimine and ketoenamine tautomers of **CID** calculated with the 6-311++G(d,p) basis set.

Method	Transition wavelength (nm)	
	Enolimine	Ketoenamine
B3LYP-GD3	405	516
B97D	475	602
B98	402	509
CAM-B3LYP-GD3	356	449
M06-GD3	392	500
PBEPBE-GD3	495	606
ωB97XD	357	450

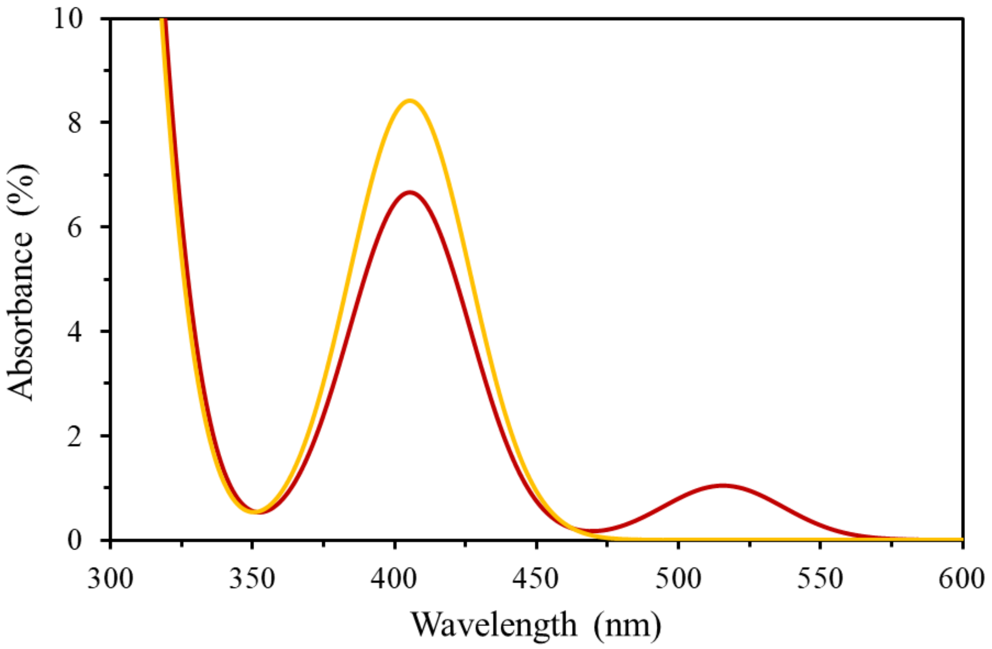

Supplementary Fig. 39. Predicted UV-vis spectra for enolimine (yellow) and ketoenamine (red) tautomers of **CID**.

Supplementary Text 8.2. Stabilization of the Ketoenamine Form in the Presence of Water

We hypothesised that the tautomeric proton shift could only occur when a hydroxyl group forms a sufficient number of hydrogen bonds with included water. To identify the critical number of hydrogen bond interactions necessary to favour tautomerism in **T1**, water molecules were placed within a hydrogen bond distance of the oxygen atoms of the enolimine and ketoenamine forms of **CID** and their positions optimised until convergence (Supplementary Figs. 40 to 43) using either a gas phase or continuum solvent model. The difference in energy between the enolimine (E_e) and ketoenamine (E_k) tautomers ($\Delta E = E_k - E_e$) calculated at the B3LYP-D3/6-311++G(d,p) level of theory was compared for models consisting of $n = 0$ to 3 water molecules (Supplementary Table 7). Gas-phase calculations showed that two hydrogen bond interactions are necessary for $\Delta E < 0$ (i.e., to stabilise the ketoenamine form), whereas in an aqueous environment described by the continuum solvent model only one hydrogen bond interaction is necessary for $\Delta E < 0$. These results suggest that a stoichiometric ratio of six interacting water molecules per asymmetric is sufficient to stabilise the ketoenamine form, which is consistent with the DVS experiments and Monte Carlo simulations.

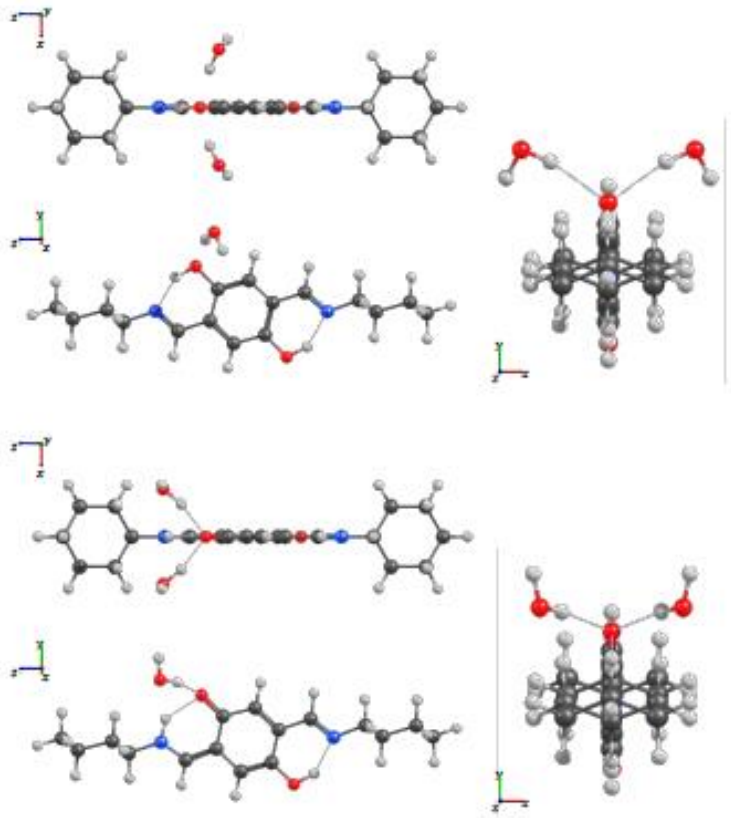

Supplementary Fig. 40. Bifurcated systems in the enolimine (top) and ketoenamine (bottom) form, optimized in the gas phase. Colours: grey, carbon; white, hydrogen; blue, nitrogen; red, oxygen.

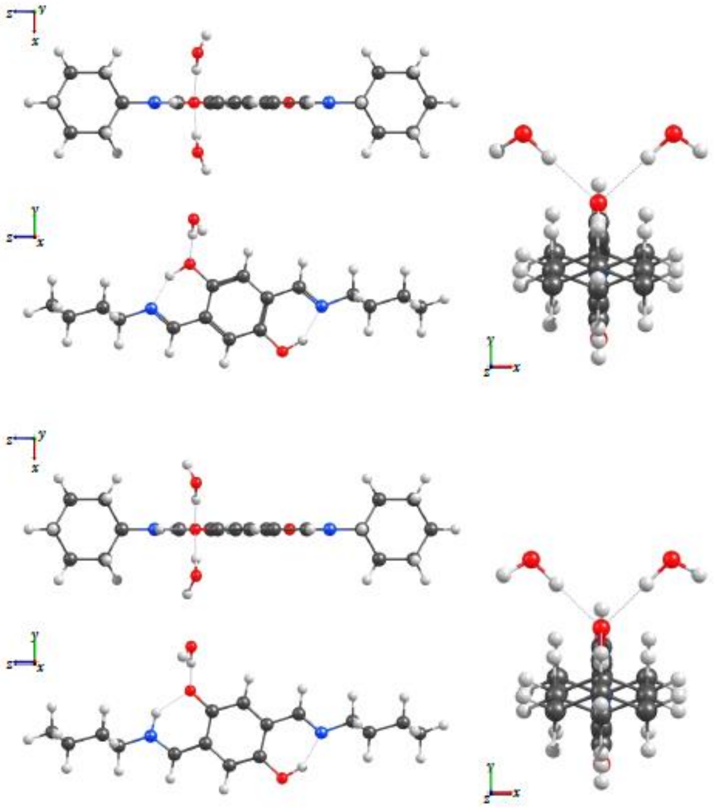

Supplementary Fig. 41. Bifurcated systems in the enolimine (top) and ketoenamine (bottom) form, in a polarizable continuum solvent model. Colours: grey, carbon; white, hydrogen; blue, nitrogen; red, oxygen.

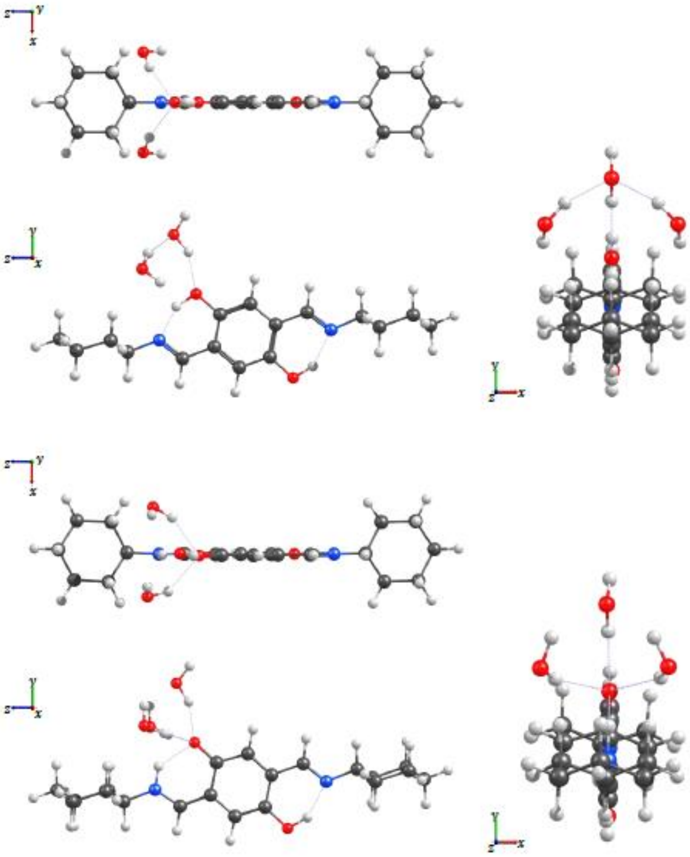

Supplementary Fig. 42. Trifurcated systems in the enolimine (top) and ketoenamine (bottom) form, optimised in the gas phase. Colours: grey, carbon; white, hydrogen; blue, nitrogen; red, oxygen.

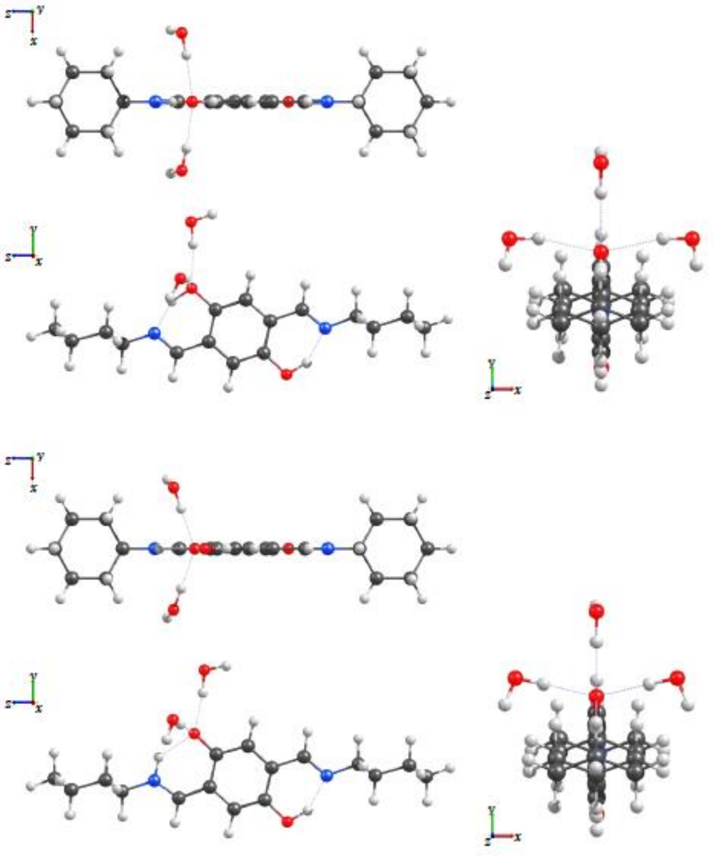

Supplementary Fig. 43. Trifurcated systems in the enolimine (top) and ketoenamine (bottom) form, in a polarizable continuum solvent model. Colours: grey, carbon; white, hydrogen; blue, nitrogen; red, oxygen.

Supplementary Table 7. Values of Δ*E* determined from DFT calculations for *n* H_2O hydrogen bond donors as shown in Supplementary Figs. 40 to 43.

Number of H_2O (*n*) hydrogen bond donors	Δ*E* (kcal mol^{-1})	
	Gas phase	Solvent
0	5.68	1.87
1	3.25	–0.72
2	–1.13	–2.48
3	–2.05	–4.44

Supplementary Text 9. Single Crystal X-ray Diffraction Analysis of Red Crystals at 25 and –173 °C

A single crystal was attached to a glass fibre using clear 5-minute epoxy. Care was taken not to coat the ends of the crystal along the crystallographic *c* axis. The crystal mounted on the diffractometer and hydrated *in situ* using a humidity controller developed in-house (Supplementary Figs. 44 and 45). Intensity data were recorded at 25 °C and 80% relative humidity. According to DVS data, the crystal contained approximately 6.5 water molecules per asymmetric unit under these conditions. The probe-accessible volumes of the channels and the intrinsic cavities were determined to be 1063, 37 and 46 Å^3, respectively. After all of the atoms of the host molecule were modelled and refined to convergence, a residual peak of 0.43 e$^-$ Å^{-3} appeared as the most intense feature of the difference electron density map. Since the highest residual peak appears at a distance of 2.84 Å from the host hydroxyl oxygen atom O9B, it is reasonable to model it as a possible water molecule. The peak was initially assigned as an oxygen atom (Og1) with its thermal parameter constrained to 0.14 Å^2 (i.e., the average U_{eqiv} value of the host hydroxyl oxygen atoms) and an unconstrained site-occupancy factor of 0.5. After 10 cycles of least-squares refinement (SHELXL) the site occupancy of O1W converges to a value of 0.23 and the distance O9B···Og1 increases to 2.97 Å. This indicates that the highest difference electron density peak within the channel is best modelled as ¼ of a water molecule, and that further attempts at locating water molecules at 25 °C are futile. However, from a difference electron density map (Supplementary Fig. 46A) we infer that water molecules experience substantial dynamic disorder but favour locations within hydrogen bonding distances of the exposed host hydroxyl groups.

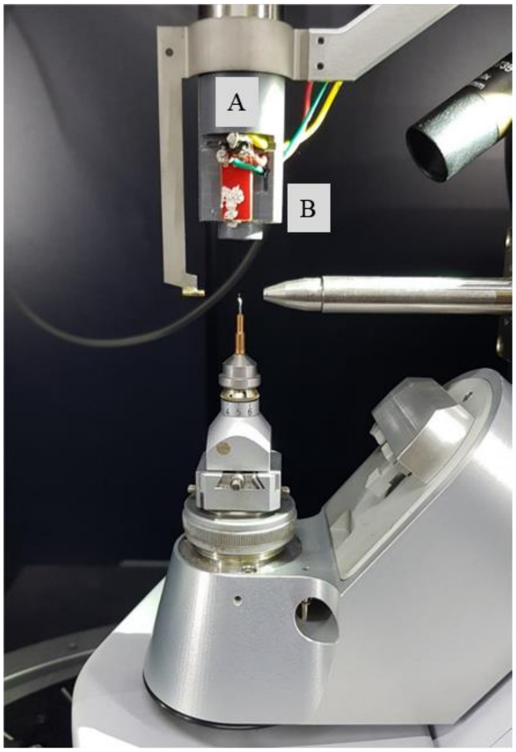

Supplementary Fig. 44. Photograph of the experimental setup for *in situ* single-crystal X-ray diffraction at controlled % RH. The device (A) attaches to the end of the otherwise unused cryostat and bathes the crystal in a stream of humid air. A relative humidity sensor (B, attached in-line) provides continuous RH readings during the experiment.

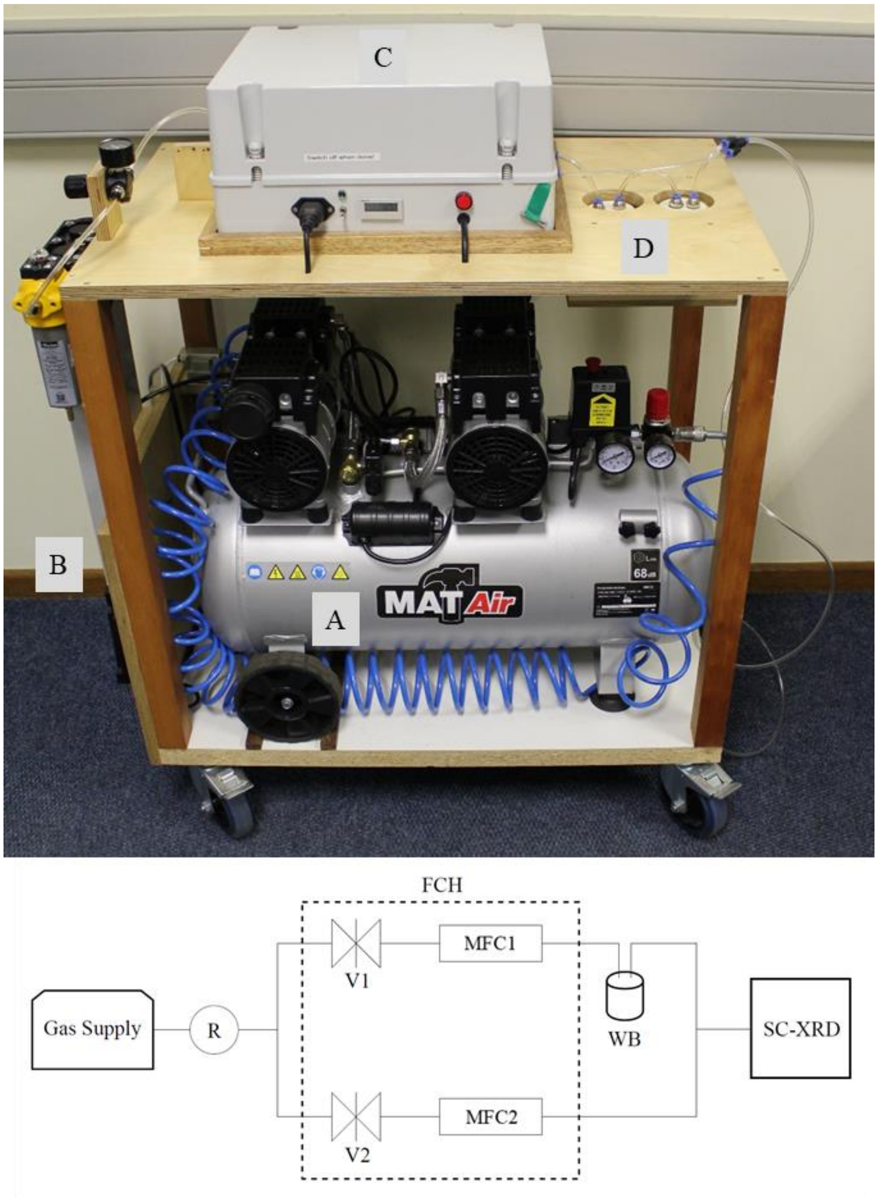

Supplementary Fig. 45. Top: photograph of the relative humidity generator used during this work. High pressure air (4 bar) from a compressor (A) passes through an air dryer (B) and into the flow controller (C). Bottom: schematic diagram of the relative humidity controller. The dried air is regulated to a pressure of 2 bar (R), and split into two separate streams, each passing through a mass-flow controller (MFC1 and MFC2). Air from MFC1 is humidified by bubbling through a water bath (WB, D) while air from MFC2 remains dry. The two streams are merged and the relative humidity is controlled by adjusting the flow rates through the two MFCs, with the total flow rate kept constant at 1 L min^{-1}. The flow-control housing (FCH, C) is illustrated with a dotted line.

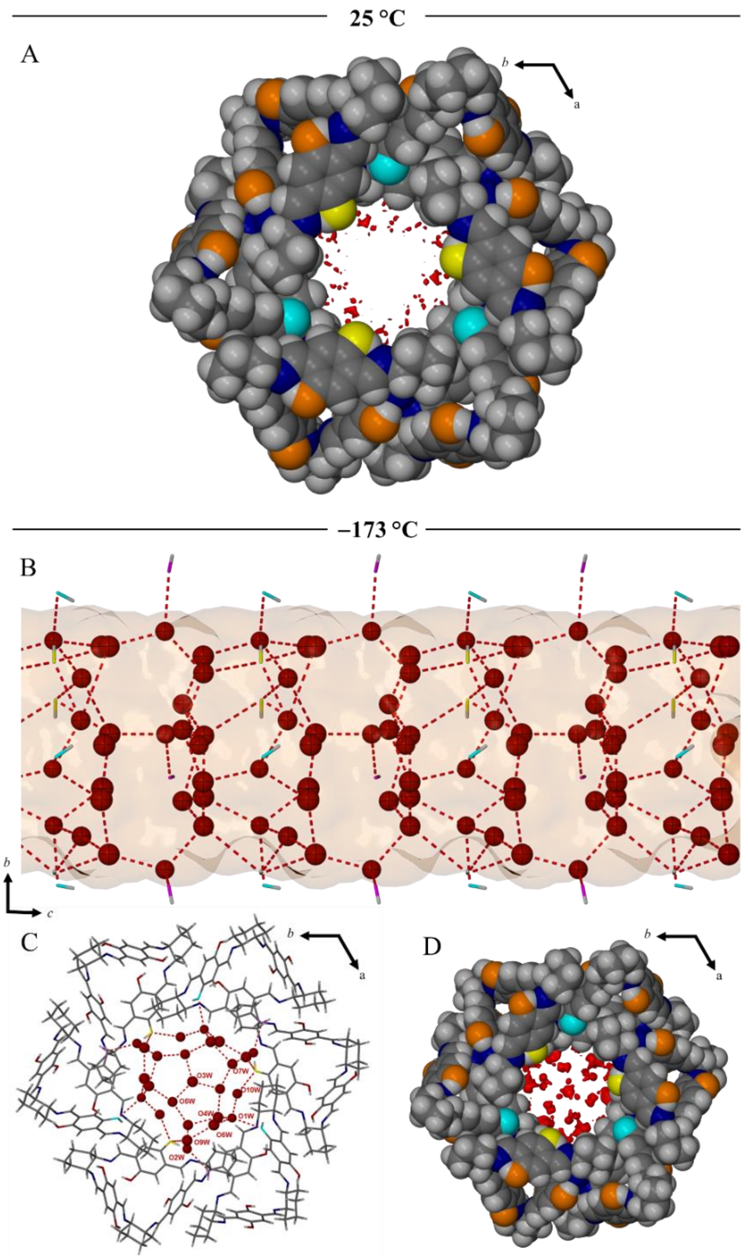

Supplementary Fig. 46. Crystal structures of **T1-R** modelled from single-crystal X-ray diffraction data acquired at 25 °C (A) and −173 °C (B-D). (A) A projection along the channel axis [001] displaying the diffuse difference electron density (0.5 e^{-} $Å^{-3}$) due to included water. (B) A perspective view along [210] with the host molecules omitted, with the exception of the O9D, O9B and O9A hydroxyl groups. Water oxygen atoms O1W to O10W were refined isotopically and are shown as spheres representing their atomic displacements at the 50% probability level. Hydrogen bond distances between oxygen atoms are shown as dashed lines. (C) A projection along the channel axis [001] displaying the water oxygen atoms O1W to O10W shown as spheres representing their atomic displacements at the 50% probability level. Hydrogen bond distances between oxygen atoms are shown as dashed lines. (D) A projection along the channel axis [001] displaying the localised difference electron density (0.5 e^{-} $Å^{-3}$) due to included water. Colours: red, difference electron density maps (in A and D) or water oxygen atoms (in B and C); yellow, O9D; cyan, O9B; magenta, O9A; orange, remaining host oxygen atoms; blue, nitrogen; grey, carbon; white, hydrogen.

SCXRD intensity data were similarly recorded at −173 °C for a hydrous crystal. The cryostat of the diffractometer was allowed to equilibrate to −173 °C; meanwhile, a crystal was exposed to a humidity-controlled stream of nitrogen gas at 25 °C and 80% relative humidity. The crystal was then rapidly transferred to the diffractometer and left to equilibrate for 10 minutes prior to intensity data collection. According to DVS data, the water content of this crystal was approximately 6.5 water molecules per asymmetric unit, and it is reasonable to expect this water content to be retained by rapid transfer to an environment of –173 °C. The probe-accessible volumes of the channels and the intrinsic cavities were determined to be 1001, 28 and 66 Å^3, respectively. After all the atoms of the host molecule were modelled and refined to convergence, ($R1$ = 0.1444 for $F_o > 4\sigma(F_o)$ and GooF = 1.560) an attempt was made to model residual electron density peaks as water molecules. Although many different approaches could be followed, we elected to assign and model the residual peaks sequentially using the protocol outlined below.

O1W: The highest residual peak of 3.21 e$^-$ Å^{-3} was assigned as a water oxygen atom (O1W), with an unconstrained site-occupancy factor of 0.5 and with its anisotropic displacement parameter constrained to 0.1. After refinement ($R1$ = 0.1378 for $F_o > 4\sigma(F_o)$ and GooF = 1.487) the site-occupancy of O1W converged to 1.03. Its site-occupancy factor was then constrained to unity and its U_{iso} value allowed to refine ($R1$ = 0.1377 for $F_o > 4\sigma(F_o)$ and GooF = 1.485), converging to U_{iso} = 0.08 Å^2.

O2W: The next highest residual peak of 3.05 e$^-$ Å^{-3} was assigned as a water oxygen atom (O2W), with an unconstrained site-occupancy factor of 0.5 and with its anisotropic displacement parameter constrained to 0.1. After refinement ($R1$ = 0.1314 for $F_o > 4\sigma(F_o)$ and GooF = 1.425) the site-occupancy of O2W also converged to 1.03. Its site-occupancy factor was then constrained to unity and its U_{iso} value allowed to refine ($R1$ = 0.1312 for $F_o > 4\sigma(F_o)$ and GooF = 1.424), converging to U_{iso} = 0.08 Å^2.

O3W: The next highest residual peak of 2.83 e$^-$ Å^{-3} was located on the crystallographic threefold axis passing through the unit cell origin. It was assigned as a water oxygen atom (O3W), with its site-occupancy factor constrained to 0.33 and with an anisotropic displacement parameter of 0.1. After refinement ($R1$ = 0.1298 for $F_o > 4\sigma(F_o)$ and GooF = 1.401) its U_{iso} value converged to 0.09 Å^2.

O4W: The next highest residual peak of 2.15 e$^-$ Å^{-3} was assigned as a water oxygen atom (O4W), with an unconstrained site-occupancy factor of 0.5 and with its anisotropic displacement parameter constrained to 0.1. After refinement ($R1$ = 0.1249 for $F_o > 4\sigma(F_o)$ and GooF = 1.362) the site-occupancy of O4W converged to 0.75. Its site-occupancy factor was then constrained to unity and its U_{iso} value allowed to refine ($R1$ = 0.1249 for $F_o > 4\sigma(F_o)$ and GooF = 1.364), converging to U_{iso} = 0.12 Å^2.

O5W: The next highest residual peak of 2.08 e$^-$ Å^{-3} was assigned as a water oxygen atom (O5W), with an unconstrained site-occupancy factor of 0.5 and with its anisotropic displacement parameter constrained to 0.15. After refinement ($R1$ = 0.1208 for $F_o > 4\sigma(F_o)$ and GooF = 1.313) the site-occupancy of O5W converged to 1.1. Its site-occupancy factor was then constrained to unity and its U_{iso} value allowed to refine ($R1$ = 0.1201 for $F_o > 4\sigma(F_o)$ and GooF = 1.304), converging to U_{iso} = 0.09 Å^2.

O6W: The next highest residual peak of 2.02 e$^-$ Å^{-3} was assigned as a water oxygen atom (O6W), with an unconstrained site-occupancy factor of 0.5 and with its anisotropic displacement parameter constrained to 0.15. After refinement ($R1$ = 0.1162 for $F_o > 4\sigma(F_o)$ and GooF = 1.275) the site-occupancy of O6W converged to 0.93. Its site-occupancy factor was then constrained to

unity and its U_{iso} value allowed to refine ($R1$ = 0.1162 for $F_o > 4\sigma(F_o)$ and GooF = 1.274), converging to U_{iso} = 0.14 Å^2.

O7W: The next highest residual peak of 1.94 e^- Å^{-3} was assigned as a water oxygen atom (O7W), with an unconstrained site-occupancy factor of 0.5 and with its anisotropic displacement parameter constrained to 0.15. After refinement ($R1$ = 0.1104 for $F_o > 4\sigma(F_o)$ and GooF = 1.221) the site-occupancy of O7W converged to 1.11. Its site-occupancy factor was then constrained to unity and its U_{iso} value allowed to refine ($R1$ = 0.1090 for $F_o > 4\sigma(F_o)$ and GooF = 1.049), converging to U_{iso} = 0.10 Å^2.

O8W: The next highest residual peak of 1.80 e^- Å^{-3} was located on the crystallographic threefold axis passing through the unit cell origin. It was assigned as a water oxygen atom (O8W), with its site-occupancy factor constrained to 0.33 and with an anisotropic displacement parameter of 0.15. After refinement ($R1$ = 0.1087 for $F_o > 4\sigma(F_o)$ and GooF = 1.049) its U_{iso} value converged to 0.17 Å^2.

O9W: The next highest residual peak of 1.43 e^- Å^{-3} was assigned as a water oxygen atom (O9W), with an unconstrained site-occupancy factor of 0.5 and with its anisotropic displacement parameter constrained to 0.15. After refinement ($R1$ = 0.1056 for $F_o > 4\sigma(F_o)$ and GooF = 1.043) the site-occupancy of O9W converged to 0.73. Its site-occupancy factor was then constrained to unity and its U_{iso} value allowed to refine ($R1$ = 0.1056 for $F_o > 4\sigma(F_o)$ and GooF = 1.048), converging to U_{iso} = 0.21 Å^2.

O10W: The next highest residual peak of 1.35 e^- Å^{-3} was assigned as a water oxygen atom (O10W), with an unconstrained site-occupancy factor of 0.5 and with its anisotropic displacement parameter constrained to 0.20. After refinement ($R1$ = 0.1024 for $F_o > 4\sigma(F_o)$ and GooF = 1.055) the site-occupancy of O10W converged to 0.96. Its site-occupancy factor was then constrained to unity and its U_{iso} value allowed to refine ($R1$ = 0.1023 for $F_o > 4\sigma(F_o)$ and GooF = 1.058), converging to U_{iso} = 0.17 Å^2.

O···O distances for possible hydrogen bonds involving O1W to O10W are listed in Supplementary Table 8. The procedure described above clearly improved the overall fit between the crystallographic model and the intensity data, as evidenced by the refinement parameters $R1$ and GooF. Although it is possible to process additional residual peaks as described above, we note that the model that included O1W to O10W (Supplementary Figs. 46B and 46C) already contained more water (8.67 H_2O molecules per asymmetric unit) than is known to be present in the hydrous crystal structure (DVS data indicate only 6.5 H_2O per asymmetric unit). Therefore, it is self-evident that the water sites O1W to O10W cannot be occupied at the same time, and that the water site occupancy factors are not reliable. Moreover, it is important to consider that, in any crystallographic model, there is a link between the site occupancy factor and the atomic displacement parameter of each atom. Although it is possible to constrain the sum of the site-occupancy factors of O1W to O10W to 6.5, such a model assumes that all of the residual electron density peaks have been accounted for (which is not the case). Indeed, given that there appeared to be no abrupt cut-off in the appearance of new difference electron density peaks, it was not obvious which of these peaks should be excluded from the model. It was therefore not possible to construct an unambiguous crystallographic model for the channel-included water molecules. From this, we postulated that the water molecules are either dynamically disordered (i.e., a 'flickering' cluster model), or that they were statically disordered into local structures that were not commensurate with the packing periodicity of the host molecules (we refer to this lack of long-range ordering as 'lattice frustration'). Supplementary Fig. 46D shows the difference electron density distribution within **T1-R** at –173 °C; despite the lack of well-defined models for the water

molecules, such representations allow meaningful side-by-side comparisons of hydrous crystal structures at different temperatures, from which we can draw conclusions about the mobility and aggregation of water within the channels.

Supplementary Table 8. O···O distances (Å) for possible hydrogen bonds involving water molecules modeled for **T1-R** at −173 °C.

	**O4W**	**O5W**	**O6W**	**O7W**	**O9W**	**O10W**	**O9A**	**O9B**	**O9D**
**O1W**	2.855(13)	-	2.812(15)	-	-	2.766(18)	-	2.856(8)	-
**O2W**	-	-	-	2.770(13)$^{\$1}$	2.63(2)	-	2.798(8)	-	-
**O3W**	-	2.703(10)	-	-	-	-	-	-	-
**O4W**		2.784(13)	-	2.813(14)$^{\$2}$	-	-	-	-	-
**O5W**			-	2.752(13)	-	-	-	-	-
**O6W**				-	2.77(2)	-	-	-	-
**O7W**					-	-	-	-	-
**O8W**					-	-	-	-	-
**O9W**						-	-	-	2.94(2)$^{\$2}$
**O10W**							-	-	2.765(17)

Symmetry operators \$1: *-y,x-y,1+z*; \$2: *-y, x-y,z*

Supplementary Text 10. Observation of Low-Temperature Water Loss

The colour of a **T1** crystal provides a convenient visual indication of its hydration state, which would otherwise be difficult to ascertain during handling at low temperature. Low-temperature (Oxford Cryosystems 800Plus cryostat) microscopy was used to identify the temperatures at which **T1-R** loses water. A single crystal of **T1-Y (**272 × 63 × 83 μm^3) was glued to a glass fibre, hydrated and rapidly cooled to –25 °C in the dry nitrogen gas stream. The crystal changed colour from red to yellow within 125 min. The experiments were repeated at incrementally lower temperatures. The lowest temperature at which **T1-R** was observed to change from red to yellow was –60 °C, occurring over a period of 70 h (Supplementary Video 4). At –70 °C (Supplementary Video 5), slight changes in colour could be discerned at the edges of the crystal after 3 days. However, complete progression from red to yellow did not occur within 5 days and the experiment was terminated.

Supplementary Text 11. Variable Temperature Single-Crystal X-ray Diffraction Analysis

Owing to the ease with which **T1-R** lose included water, a strategy was devised to inhibit water loss during variable-temperature single-crystal X-ray diffraction (VT-SCXRD) experiments. We had already established that water loss occurs along the crystallographic *c* axis and is accompanied by a change in colour from red to yellow. However, we found that covering one end of the crystal with fast-setting (5 min) epoxy prevented water release (Supplementary Video 6). We therefore sealed the [001] and [00$\bar{1}$] faces of a hydrous (red) crystal by coating them with five-minute epoxy. This method of protecting the crystal against water loss was then tested by recording time-lapse photomicrographs of the crystal at –25 °C (Supplementary Video 7). The crystal remained red for 12 h, followed by gradual water loss to render the crystal completely yellow after 36 h. The change in colour did not occur along the *c* axis, but at the unprotected side of the crystal nearest to the cryostat. From these observations we ascertained that it was necessary to cover the entire crystal with epoxy in order to prevent water loss during the acquisition of variable temperature SCXRD intensity data.

A protected red crystal was mounted on a single-crystal X-ray diffractometer and equilibrated to an initial temperature of –25 °C in the dry nitrogen stream of the cryostat. A total of 31 full intensity datasets were recorded at intervals of 5 °C during cooling from –25 to –175 °C (Supplementary Table 9). This was followed by similarly recording 30 full datasets upon heating back to –25 °C (Supplementary Table 10). Between datasets the temperature was adjusted to the next value at a rate of approximately 4 °C min^{-1} and equilibrated for 5 min. The duration of each data collection was approximately 4 h and the process (adjustment of temperature and collection of intensity data) was automated using the Bruker Apex-3 software. A similar procedure was followed for an anhydrous (yellow) crystal (31 full datasets upon cooling from –25 to –175 °C (Supplementary Table 11), followed by 30 full datasets upon heating back to –25 °C (Supplementary Table 12). This process yielded a total of 61 VT-SCXRD datasets each for the hydrous and anhydrous crystals. In both the hydrous and anhydrous forms, the host crystal structure does not appear to undergo any thermally-induced structural rearrangements that can be construed as polymorphic phase changes. Moreover, the host packing arrangements for the hydrous and anhydrous forms are, in principle, the same. Therefore, it is reasonable to assume that any subtle structural differences between the red and yellow forms at the same temperature are solely due to the presence of water (i.e., arising from water-water and water-host interactions). From the 122 crystal structures for cooling and heating in the range –25 to –175 °C, it is possible

to assess (i) the effects of temperature on the crystallographic parameters of both crystal forms, (ii) the evolution of difference electron density clouds due to water in the hydrous form as a function of temperature, and (iii) the temperature-dependent influence of included water on structural features of the host. These are discussed in turn below.

Supplementary Table 9. Unit cell parameters for **T1-R** during cooling from –25 to –175 °C.

Temperature (°C)	*a*, *b* (Å)	*c* (Å)	Volume (Å^3)
–25	50.6380(11)	9.5809(3)	21276.0(11)
–30	50.6499(9)	9.5709(3)	21263.8(10)
–35	50.6493(9)	9.5618(3)	21243.1(10)
–40	50.6490(10)	9.5545(3)	21226.6(11)
–45	50.6590(10)	9.5492(3)	21223.2(11)
–50	50.6654(11)	9.5335(3)	21193.7(11)
–55	50.6719(10)	9.5208(3)	21170.8(11)
–60	50.6872(11)	9.5134(3)	21167.2(11)
–65	50.7402(11)	9.4927(3)	21165.3(11)
–70	50.7988(10)	9.4574(3)	21135.3(11)
–75	50.8246(10)	9.4480(3)	21135.8(11)
–80	50.8302(9)	9.4403(3)	21123.2(10)
–85	50.8329(9)	9.4345(3)	21112.5(10)
–90	50.8304(9)	9.4292(3)	21098.5(10)
–95	50.8291(9)	9.4248(3)	21087.6(10)
–100	50.8251(9)	9.4211(3)	21076.0(10)
–105	50.8203(10)	9.4165(3)	21061.7(11)
–110	50.8159(9)	9.4144(3)	21053.4(9)
–115	50.8062(9)	9.4096(3)	21034.6(9)
–120	50.8009(9)	9.4065(3)	21023.3(10)
–125	50.7962(9)	9.4033(3)	21012.3(10)
–130	50.7907(9)	9.4010(3)	21002.6(10)
–135	50.7840(9)	9.3979(3)	20990.1(9)
–140	50.7745(9)	9.3957(3)	20977.4(9)
–145	50.7712(9)	9.3931(3)	20968.8(9)
–150	50.7611(8)	9.3890(3)	20951.3(9)
–155	50.7587(9)	9.3868(3)	20944.5(9)
–160	50.7513(9)	9.3832(3)	20930.3(9)
–165	50.7410(9)	9.3785(3)	20911.3(10)
–170	50.7350(9)	9.3757(3)	20900.2(10)
–175	50.7349(9)	9.3740(3)	20896.3(10)

Supplementary Table 10. Unit cell parameters for **T1-R** during heating from −175 to −25 °C.

Temperature (°C)	*a*, *b* (Å)	*c* (Å)	Volume (Å^3)
–170	50.7395(9)	9.3757(3)	21235.5(10)
–165	50.7428(9)	9.3780(3)	21216.3(10)
–160	50.7492(9)	9.3808(3)	21194.1(11)
–155	50.7559(9)	9.3844(3)	21179.4(10)
–150	50.7641(9)	9.3875(3)	21164.1(11)
–145	50.7694(9)	9.3904(3)	21154.7(11)
–140	50.7757(9)	9.3941(3)	21151.0(11)
–135	50.7818(9)	9.3972(3)	21153.3(11)
–130	50.7893(9)	9.4000(3)	21148.7(11)
–125	50.7954(9)	9.4018(3)	21142.6(11)
–120	50.8042(9)	9.4050(3)	21133.8(10)
–115	50.8077(9)	9.4079(3)	21120.5(11)
–110	50.8156(9)	9.4112(3)	21111.7(11)
–105	50.8222(9)	9.4135(3)	21098.1(10)
–100	50.8317(9)	9.4169(3)	21086.3(10)
–95	50.8364(9)	9.4215(3)	21072.1(10)
–90	50.8385(9)	9.4260(3)	21056.6(10)
–85	50.8425(10)	9.4306(3)	21046.0(10)
–80	50.8402(10)	9.4354(3)	21032.1(10)
–75	50.8394(9)	9.4416(3)	21022.7(10)
–70	50.8309(10)	9.4487(3)	21008.3(10)
–65	50.8154(10)	9.4572(3)	20999.2(10)
–60	50.7927(10)	9.4677(3)	20986.8(10)
–55	50.7557(10)	9.4805(3)	20974.8(10)
–50	50.7235(10)	9.4942(3)	20961.3(10)
–45	50.7056(10)	9.5051(3)	20950.5(10)
–40	50.6976(9)	9.5150(3)	20936.8(10)
–35	50.6965(10)	9.5220(3)	20923.2(10)
–30	50.6991(9)	9.5310(3)	20911.7(10)
–25	50.6859(9)	9.5446(3)	20903.9(10)

Supplementary Table 11. Unit cell parameters for **T1-Y** during cooling from −25 to −175 °C.

Temperature (°C)	*a*, *b* (Å)	*c* (Å)	Volume ($Å^3$)
–25	50.7299(19)	9.5758(6)	21342(2)
–30	50.7348(20)	9.5694(6)	21332(2)
–35	50.7452(20)	9.5641(6)	21329(2)
–40	50.7500(21)	9.5560(6)	21315(2)
–45	50.7629(22)	9.5492(6)	21310(2)
–50	50.7720(23)	9.5424(7)	21303(2)
–55	50.7764(24)	9.5333(7)	21286(3)
–60	50.7859(25)	9.5262(7)	21278(3)
–65	50.7890(26)	9.5193(8)	21265(3)
–70	50.7843(28)	9.5085(8)	21237(3)
–75	50.8029(29)	9.5059(8)	21247(3)
–80	50.8041(30)	9.4965(9)	21227(3)
–85	50.8159(30)	9.4930(9)	21229(3)
–90	50.8167(32)	9.4872(9)	21217(3)
–95	50.8180(32)	9.4807(9)	21203(3)
–100	50.8082(34)	9.4716(10)	21175(4)
–105	50.8140(34)	9.4647(10)	21164(4)
–110	50.8022(35)	9.4559(10)	21135(4)
–115	50.8061(38)	9.4538(11)	21133(4)
–120	50.8159(37)	9.4508(11)	21135(4)
–125	50.8052(38)	9.4412(11)	21104(4)
–130	50.7979(39)	9.4362(11)	21087(4)
–135	50.7916(40)	9.4301(11)	21068(4)
–140	50.7886(40)	9.4252(12)	21055(4)
–145	50.7961(42)	9.4250(12)	21061(4)
–150	50.7884(42)	9.4231(12)	21050(4)
–155	50.7689(45)	9.4130(13)	21011(5)
–160	50.7558(45)	9.4051(13)	20983(5)
–165	50.7406(46)	9.4032(13)	20966(5)
–170	50.7241(45)	9.3976(13)	20940(5)
–175	50.7172(47)	9.3991(13)	20938(5)

Supplementary Table 12. Unit cell parameters for **T1-Y** during heating from −175 to −25 °C.

Temperature (°C)	*a*, *b* (Å)	*c* (Å)	Volume ($Å^3$)
–170	50.7212(45)	9.3992(13)	20941(5)
–165	50.7532(45)	9.4069(13)	20985(5)
–160	50.7552(45)	9.4059(13)	20984(5)
–155	50.7748(45)	9.4113(13)	21012(5)
–150	50.7976(45)	9.4223(13)	21056(5)
–145	50.7745(45)	9.4153(13)	21021(5)
–140	50.7960(43)	9.4293(12)	21070(4)
–135	50.7894(42)	9.4309(12)	21068(4)
–130	50.7999(42)	9.4400(12)	21097(4)
–125	50.7673(23)	9.4479(7)	21088(2)
–120	50.7818(21)	9.4543(6)	21114(2)
–115	50.7865(21)	9.4584(6)	21127(2)
–110	50.7899(21)	9.4627(6)	21140(2)
–105	50.7991(21)	9.4679(6)	21159(2)
–100	50.7985(20)	9.4725(6)	21169(2)
–95	50.8064(19)	9.4790(5)	21189.9(19)
–90	50.8052(19)	9.4833(5)	21198.6(19)
–85	50.8099(18)	9.4884(5)	21213.9(19)
–80	50.8112(18)	9.4935(5)	21226.4(19)
–75	50.8214(18)	9.4996(5)	21248.5(19)
–70	50.8203(16)	9.5056(5)	21261.0(17)
–65	50.8270(16)	9.5104(5)	21277.4(17)
–60	50.8275(16)	9.5166(4)	21291.7(16)
–55	50.8244(16)	9.5223(4)	21301.8(16)
–50	50.7976(14)	9.5280(4)	21292.1(15)
–45	50.8038(14)	9.5374(5)	21318.3(15)
–40	50.7871(14)	9.5446(4)	21320.4(15)
–35	50.7653(13)	9.5516(4)	21317.7(14)
–30	50.7374(17)	9.5593(5)	21311.4(18)
–25	50.7334(13)	9.5675(4)	21326.4(14)

The presence of water results in sharp elongation of the crystallographic *a* and *b* axes and concomitant contraction of the *c* axis during cooling between –50 and –75 °C (Fig. 3d). Correspondingly, the width of the channel unit increases and its length decreases in this temperature range. Further cooling reverses the trends in both the axis lengths and the channel geometry. The effect of this can be seen by studying the thermal expansion. Upon cooling from –25 to –175 °C, the anhydrous crystal experiences zero linear thermal expansion along *a* and *b* (Supplementary Table 13, Supplementary Fig. 47A) and uniform positive linear thermal expansion (PTE) along *c* (Supplementary Fig. 47B) is similarly featureless and there is minimal hysteresis upon heating from –25 back to –175 °C. In sharp contrast, the hydrous crystal initially experiences modest negative thermal expansion (NTE) along *a* and *b* (Supplementary Fig. 47D) upon cooling from –25 back to –55 °C, followed by an abrupt eightfold increase in the magnitude of NTE between –55 and –75 °C, thereafter switching to modest PTE from –90 to –175 °C. In the same ranges, the *c* axis (Supplementary Fig. 47E) experiences changes in its magnitudes of PTE, thereby compensating for the changes in *a* and *b* such that a uniform trend is observed for the volumetric (Supplementary Fig. 47F) thermal expansion over the entire temperature range investigated. Upon heating, the changes in unit cell parameters are reversed in the range –175 to –80 °C, with a moderate amount of hysteresis present in the range –75 to –25 °C. This hysteresis is also observed for volumetric thermal expansion.

Supplementary Table 13. Linear and volumetric thermal expansion coefficients (in MK^{-1}) of **T1-R** and **T1-Y** upon cooling and heating. Owing to changes in the thermal profiles of **T1-R**, three different temperature ranges are specified.

Crystal	Temperature Range (°C)	*X*1 = *X*2	*X*3	α_V
**T1-R** Cooling	–25 → –55	–20 ± 2	200 ± 10	165 ± 9
	–55 → –75	–164 ± 12	425 ± 35	117 ± 23
	–90 → –175	24 ± 2	68 ± 1	117 ± 13
**T1-R** Heating	–55 → –25	–38 ± 9	212 ± 7	143 ± 11
	–75 → –55	–81 ± 11	205 ± 12	52 ± 8
	–170 → –90	25.7 ± 0.4	65 ± 1	117 ± 1
**T1-Y** Cooling	–25 → –175	0 ± 3	131 ± 3	132 ± 4
**T1-Y** Heating	–170 → –25	3 ± 3	122 ± 2	130 ± 4

Thermal expansion coefficients were calculated using PASCal[164].

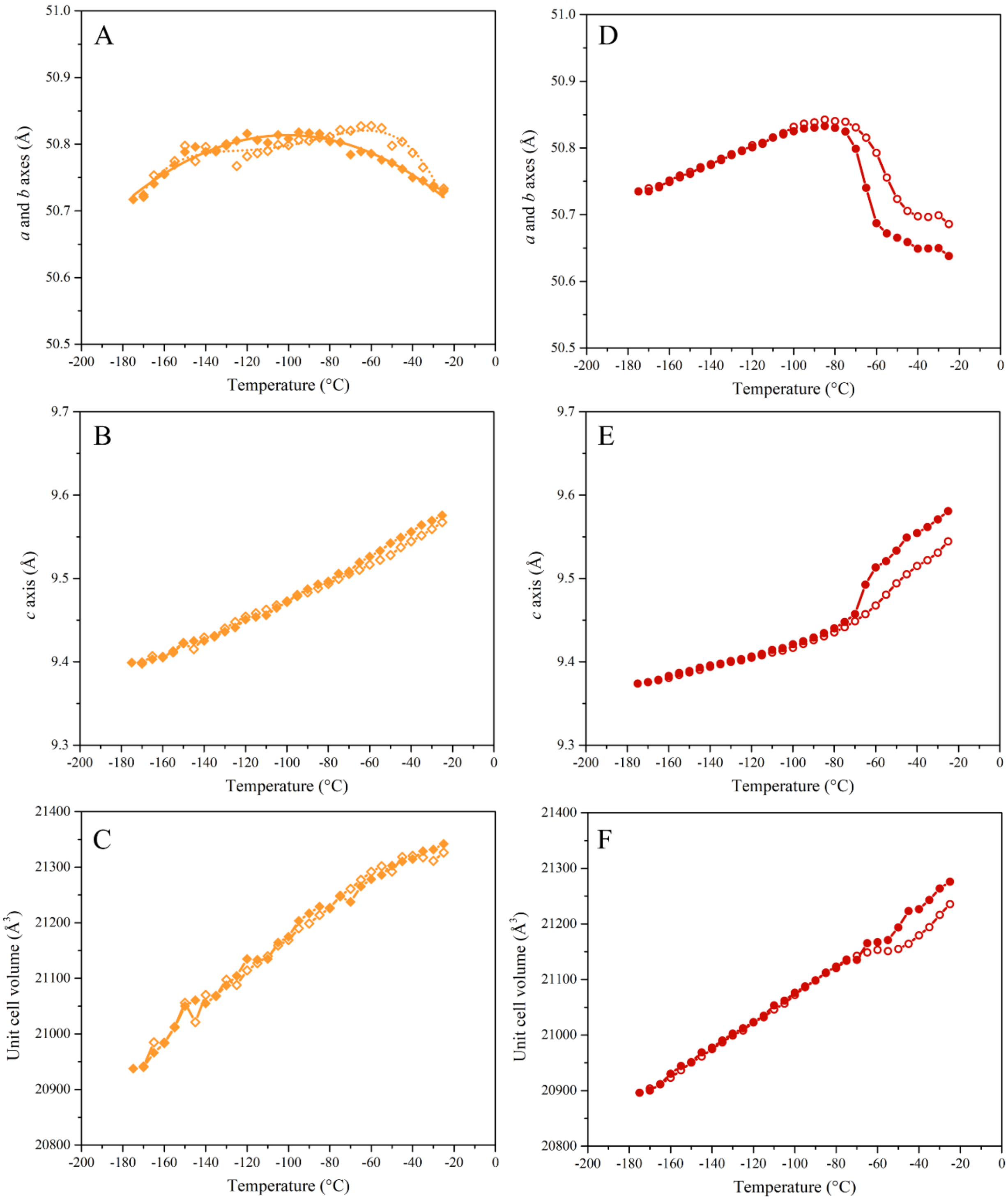

Supplementary Fig. 47. Unit cell parameters for **T1** during cooling (closed symbols) and heating (open symbols) between −25 and −175 °C: (A) *a* and *b* axes, (B) *c* axis and (C) unit cell volume of **T1-Y** and (D) *a* and *b* axes, (E) *c* axis and (F) unit cell volume of **T1-R**.

The difference electron density analysis of water in the channels of **T1-R** was carried out using an electron density threshold of 1 e^{-} Å^{-3} for both cooling (Supplementary Videos 8 and 9) and heating (Supplementary Videos 10 and 11). No appreciable electron density peaks (>1 e^{-} Å^{-3}) were observed at temperatures between –25 and –45 °C; this is consistent with the low intensities of the 10 highest difference electron density peaks in the channel during the cooling (Supplementary Table 14). Upon further cooling (–35 to –60 °C), dynamic electron density clouds emerged near the exposed host hydroxyl groups. Electron density at the centre of the channel first emerged at –65 °C, followed by a sharp increase in the overall distribution of electron density in the channel at –70 °C. While the positions of the electron clouds varied at higher temperatures, their positions were more localised at < –70 °C. Further cooling resulted in a gradual increase in the concentration of electron density at these sites, with the largest peaks observed at –175 °C. Upon heating, the electron density distribution is maintained, with decreasing concentration, until –60 °C. Thereafter, appreciable electron density was only observed near the hydroxyl groups. Indeed, the trend in the intensities of the 10 highest difference electron density peaks over the range –175 to –25 °C was reversed relative to those for cooling (Supplementary Table 15).

It is evident that water forms hydrogen bonding interactions with the exposed hydroxyl groups in the channels of the red crystals, albeit without long-range order. If included water imposes a temperature-dependent structural change on **T1-R**, then it is likely that the positions of the hydroxyl groups will be influenced relative to those of the anhydrous crystals. Hence, it is useful to monitor the displacement of O9D, O9B and O9A with temperature to determine the influence of water on **T1-R**. A centroid Θ_x (where x = D, B or A), of the symmetry-equivalent oxygen atoms of the exposed hydroxyl groups (Supplementary Fig. 48), served as a convenient reference point between the structures (the centroid is located on the vector along the centre of the channel). The distances between the hydroxyl oxygen atoms and Θ_x, O9x··· Θ_x, for the cooling and heating cycles of **T1-Y** and **T1-R** were compared for O9D (Supplementary Fig. 49), O9B (Supplementary Fig. 50) and O9A (Supplementary Fig. 51). The lengths O9D···Θ_D, O9B···Θ_B and O9A···Θ_A of the anhydrous crystal remain constant during both cooling and heating between –25 and –175 °C. In contrast, the presence of water in the channel resulted in displacements of the hydroxyl groups in the same temperature range (*cf* shifts observed in the unit cell parameters, as shown in Supplementary Fig. 47). The distance O9D···Θ_D remained constant upon cooling to –55 °C and, upon further cooling, experienced a sharp increase between –60 and –70 °C, after which it increased gradually until –175 °C. Heating resulted in reversal of the features observed for O9D···Θ_D during cooling, with minimal hysteresis. The distance O9B···Θ_B decreased uniformly upon cooling over the entire temperature range, with no apparent hysteresis upon heating. The cooling profile of O9A···Θ_A was similar to that of O9D···Θ_D. The displacements due solely to hydration were isolated by considering the differences between the O9x··· Θ_x distances for the hydrous and anhydrous crystals during cooling and heating, as shown in Supplementary Figs. 52 and 53, respectively.

Supplementary Table 14. The 10 highest residual electron density peaks (e^{-} $Å^{-3}$) remaining after final crystallographic refinement of the host atoms in **T1-R** during cooling from −25 to −175 °C.

Temperature (°C)	Electron density peak height (e^{-} $Å^{-3}$) in descending order
–25	0.71, 0.70, 0.69, 0.68, 0.66, 0.65, 0.51, 0.41, 0.39, 0.39
–30	0.79, 0.75, 0.73, 0.68, 0.65, 0.62, 0.49, 0.46, 0.42, 0.40
–35	0.84, 0.77, 0.72, 0.70, 0.68, 0.64, 0.46, 0.46, 0.45, 0.39
–40	0.87, 0.78, 0.76, 0.71, 0.68, 0.66, 0.48, 0.48, 0.40, 0.40
–45	0.92, 0.85, 0.70, 0.69, 0.68, 0.54, 0.51, 0.49, 0.40, 0.35
–50	**1.00**, 0.88, 0.70, 0.69, 0.68, 0.57, 0.56, 0.44, 0.42, 0.38
–55	**1.00**, 0.94, 0.72, 0.71, 0.70, 0.63, 0.58, 0.48, 0.41, 0.38
–60	**1.09, 1.00**, 0.78, 0.74, 0.69, 0.69, 0.68, 0.56, 0.56, 0.56
–65	**1.27, 1.21**, 0.89, 0.81, 0.80, 0.77, 0.74, 0.71, 0.64, 0.60
–70	**1.55, 1.37, 1.16, 1.12, 1.00**, 0.91, 0.90, 0.80, 0.79, 0.54
–75	**1.62, 1.49, 1.46, 1.28, 1.16, 1.08**, 0.97, 0.83, 0.83, 0.77
–80	**1.76, 1.66, 1.64, 1.38, 1.22, 1.20**, 0.98, 0.87, 0.86, 0.82
–85	**1.89, 1.81, 1.72, 1.47, 1.36, 1.27**, 0.96, 0.96, 0.95, 0.88
–90	**2.11, 1.87, 1.79, 1.58, 1.46, 1.41, 1.11, 1.06**, 0.98, 0.93
–95	**2.26, 1.99, 1.85, 1.63, 1.54, 1.49, 1.19, 1.10, 1.03**, 0.99
–100	**2.46, 1.99, 1.95, 1.68, 1.64, 1.60, 1.34, 1.17, 1.09, 1.03**
–105	**2.57, 2.08, 1.97, 1.74, 1.69, 1.65, 1.42, 1.12, 1.04, 1.04**
–110	**2.66, 2.18, 2.07, 1.87, 1.80, 1.69, 1.58, 1.20, 1.11, 1.10**
–115	**2.89, 2.20, 2.14, 1.96, 1.86, 1.77, 1.76, 1.30, 1.27, 1.14**
–120	**3.03, 2.26, 2.18, 1.99, 1.92, 1.86, 1.77, 1.35, 1.33, 1.32**
–125	**3.19, 2.33, 2.22, 2.02, 2.01, 1.96, 1.82, 1.39, 1.38, 1.33**
–130	**3.27, 2.37, 2.31, 2.10, 2.06, 2.04, 1.93, 1.50, 1.43, 1.36**
–135	**3.42, 2.42, 2.29, 2.14, 2.10, 2.08, 2.01, 1.48, 1.41, 1.40**
–140	**3.46, 2.51, 2.40, 2.23, 2.13, 2.10, 2.09, 1.62, 1.46, 1.28**
–145	**3.58, 2.54, 2.41, 2.21, 2.20, 2.14, 2.06, 1.62, 1.51, 1.42**
–150	**3.79, 2.55, 2.41, 2.32, 2.29, 2.12, 2.10, 1.64, 1.58, 1.47**
–155	**3.82, 2.66, 2.45, 2.34, 2.28, 2.18, 2.18, 1.76, 1.59, 1.49**
–160	**3.89, 2.65, 2.46, 2.37, 2.25, 2.19, 2.18, 1.78, 1.58, 1.47**
–165	**3.79, 2.79, 2.56, 2.39, 2.30, 2.24, 2.20, 1.89, 1.66, 1.50**
–170	**4.11, 2.78, 2.55, 2.48, 2.27, 2.26, 2.21, 1.84, 1.73, 1.49**
–175	**3.99, 2.85, 2.64, 2.52, 2.36, 2.30, 2.21, 1.86, 1.74, 1.62**

Note: peak values ≥1 e^{-} $Å^{-3}$ are shown in boldface.

Supplementary Table 15. The 10 highest residual electron density peaks (e⁻ $Å^{-3}$) remaining after final crystallographic refinement of the host atoms in **T1-R** during heating from −175 to −25 °C.

Temperature (°C)	Electron density peak height (e⁻ $Å^{-3}$) in descending order
–170	**2.87, 2.09, 1.90, 1.88, 1.81, 1.72, 1.64, 1.33, 1.21, 1.19**
–165	**3.86, 2.71, 2.54, 2.40, 2.31, 2.23, 2.21, 1.79, 1.68, 1.49**
–160	**3.86, 2.69, 2.47, 2.37, 2.28, 2.16, 2.14, 1.73, 1.62, 1.53**
–155	**3.70, 2.61, 2.46, 2.29, 2.23, 2.14, 2.07, 1.72, 1.53, 1.44**
–150	**3.64, 2.57, 2.39, 2.25, 2.21, 2.08, 2.06, 1.65, 1.53, 1.42**
–145	**3.51, 2.53, 2.36, 2.21, 2.12, 2.12, 2.07, 1.59, 1.51, 1.40**
–140	**3.58, 2.49, 2.35, 2.15, 2.10, 2.10, 2.02, 1.57, 1.47, 1.41**
–135	**3.39, 2.46, 2.34, 2.10, 2.06, 2.03, 2.03, 1.54, 1.41, 1.32**
–130	**3.28, 2.38, 2.26, 2.06, 2.02, 2.00, 1.97, 1.52, 1.41, 1.35**
–125	**3.25, 2.36, 2.21, 2.02, 1.99, 1.94, 1.86, 1.40, 1.35, 1.33**
–120	**3.02, 2.28, 2.14, 1.99, 1.98, 1.83, 1.74, 1.37, 1.31, 1.14**
–115	**2.87, 2.23, 2.10, 1.94, 1.85, 1.77, 1.72, 1.32, 1.28, 1.12**
–110	**2.67, 2.15, 2.08, 1.91, 1.79, 1.72, 1.50, 1.21, 1.08, 1.04**
–105	**2.56, 2.10, 1.99, 1.80, 1.67, 1.65, 1.41, 1.15, 1.06, 1.05**
–100	**2.35, 2.04, 1.96, 1.68, 1.62, 1.58, 1.28, 1.14, 1.05, 1.02**
–95	**2.17, 1.97, 1.88, 1.69, 1.56, 1.45, 1.14, 1.05, 1.04, 1.01**
–90	**2.06, 1.91, 1.77, 1.50, 1.47, 1.37, 1.07, 1.03, 1.02**, 0.93
–85	**1.85, 1.77, 1.67, 1.48, 1.37, 1.29**, 0.99, 0.99, 0.98, 0.89
–80	**1.79, 1.59, 1.58, 1.37, 1.29, 1.14**, 0.98, 0.92, 0.90, 0.87
–75	**1.69, 1.52, 1.47, 1.26, 1.18, 1.05**, 0.97, 0.87, 0.83, 0.82
–70	**1.54, 1.42, 1.22, 1.17, 1.06,** 0.94, 0.93, 0.78, 0.78, 0.74
–65	**1.44, 1.30**, 0.98, 0.97, 0.91, 0.90, 0.79, 0.75, 0.74, 0.74
–60	**1.29, 1.16**, 0.89, 0.84, 0.79, 0.75, 0.71, 0.70, 0.67, 0.55
–55	**1.14**, 0.97, 0.76, 0.74, 0.73, 0.69, 0.66, 0.56, 0.48, 0.47
–50	**1.00**, 0.94, 0.78, 0.71, 0.70, 0.70, 0.58, 0.58, 0.39, 0.38
–45	0.95, 0.87, 0.71, 0.69, 0.67, 0.56, 0.52, 0.43, 0.37, 0.35
–40	0.89, 0.83, 0.73, 0.69, 0.68, 0.51, 0.50, 0.39, 0.33, 0.32
–35	0.83, 0.78, 0.72, 0.71, 0.70, 0.65, 0.49, 0.44, 0.43, 0.42
–30	0.77, 0.77, 0.66, 0.65, 0.63, 0.47, 0.45, 0.39, 0.36, 0.28
–25	0.75, 0.74, 0.74, 0.67, 0.65, 0.63, 0.45, 0.44, 0.34, 0.32

Note: peak values ≥1 e⁻ $Å^{-3}$ are shown in boldface.

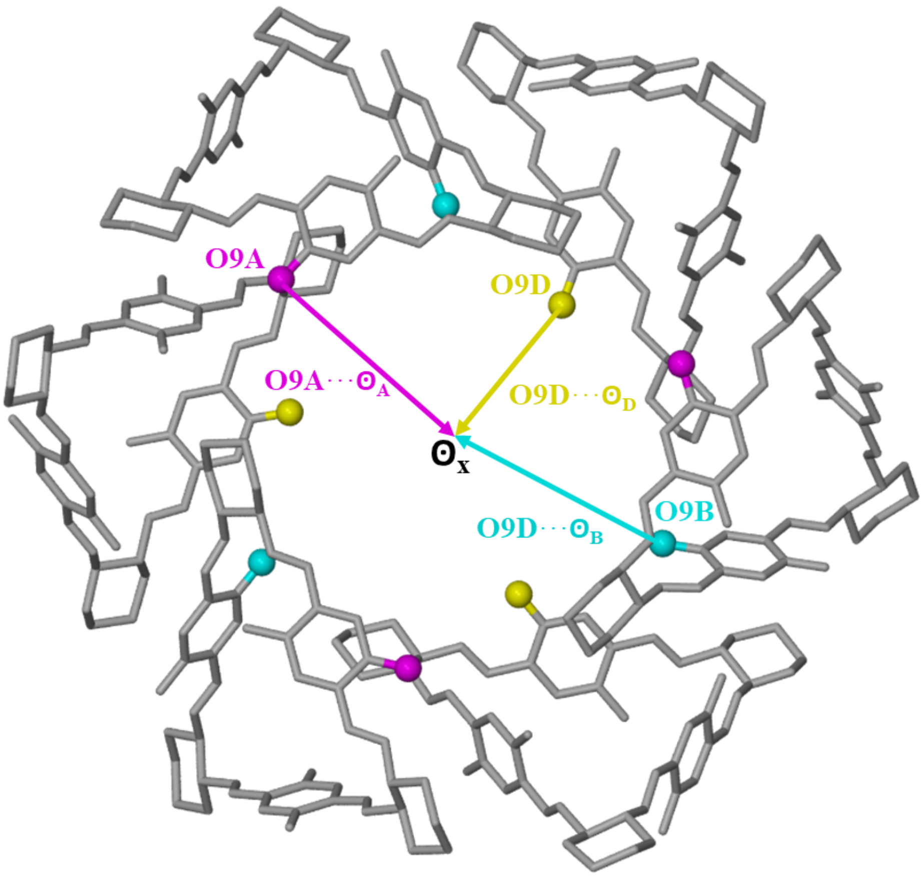

Supplementary Fig. 48. A projection along the channel axis [00$\bar{1}$] of **T1** showing the centroids ʘ (all superimposed in this projection) of each symmetry-related set of the three symmetry-independent hydroxyl groups. The arrows indicate the displacement distances O9D···ʘ$_x$ between the oxygen atom and the centroid. Hydroxyl oxygen atoms O9D, O9B and O9A are shown as spheres and hydrogen atoms have been omitted for clarity Colours: yellow, O9D; cyan, O9B; magenta, O9A; grey, remaining atoms.

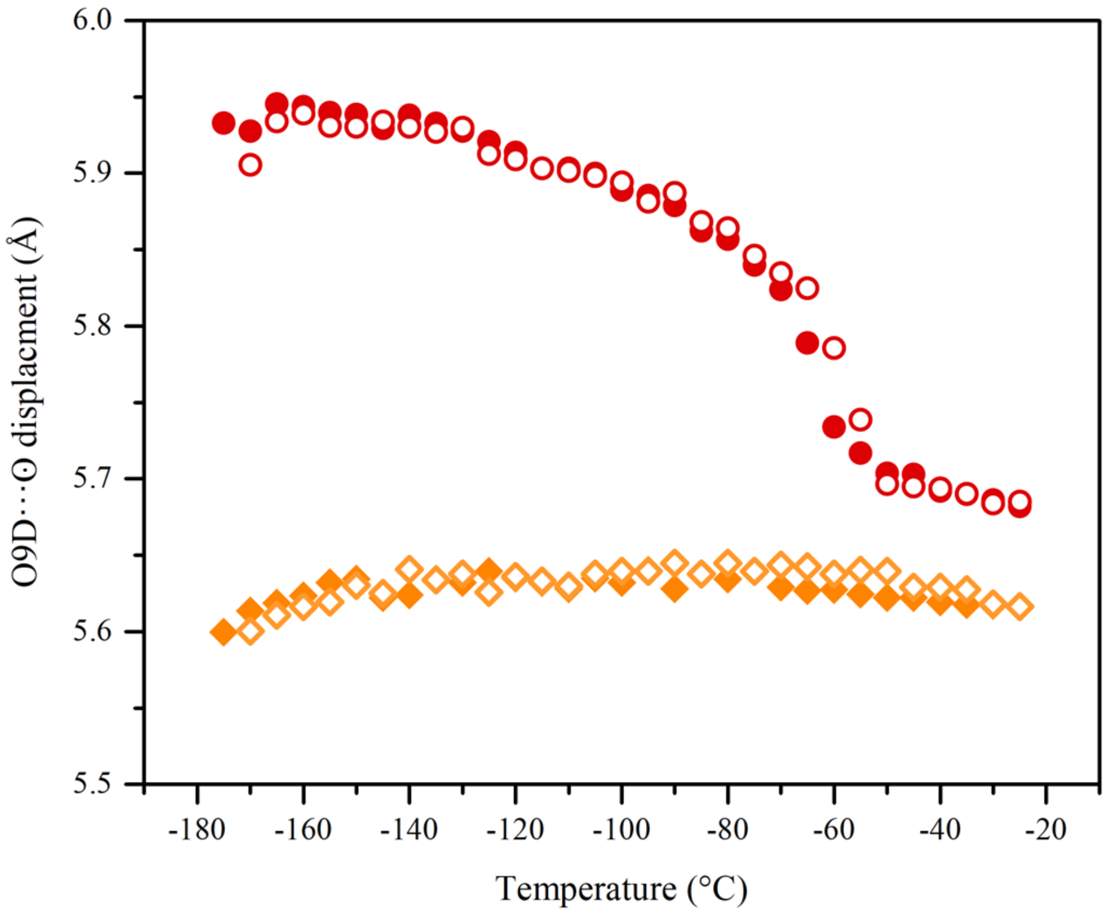

Supplementary Fig. 49. O9D···O$_D$ displacement plots determined for **T1-R** (red circles) and **T1-Y** (orange diamonds) during cooling (closed symbols) and heating (open symbols) between −25 and −175 °C.

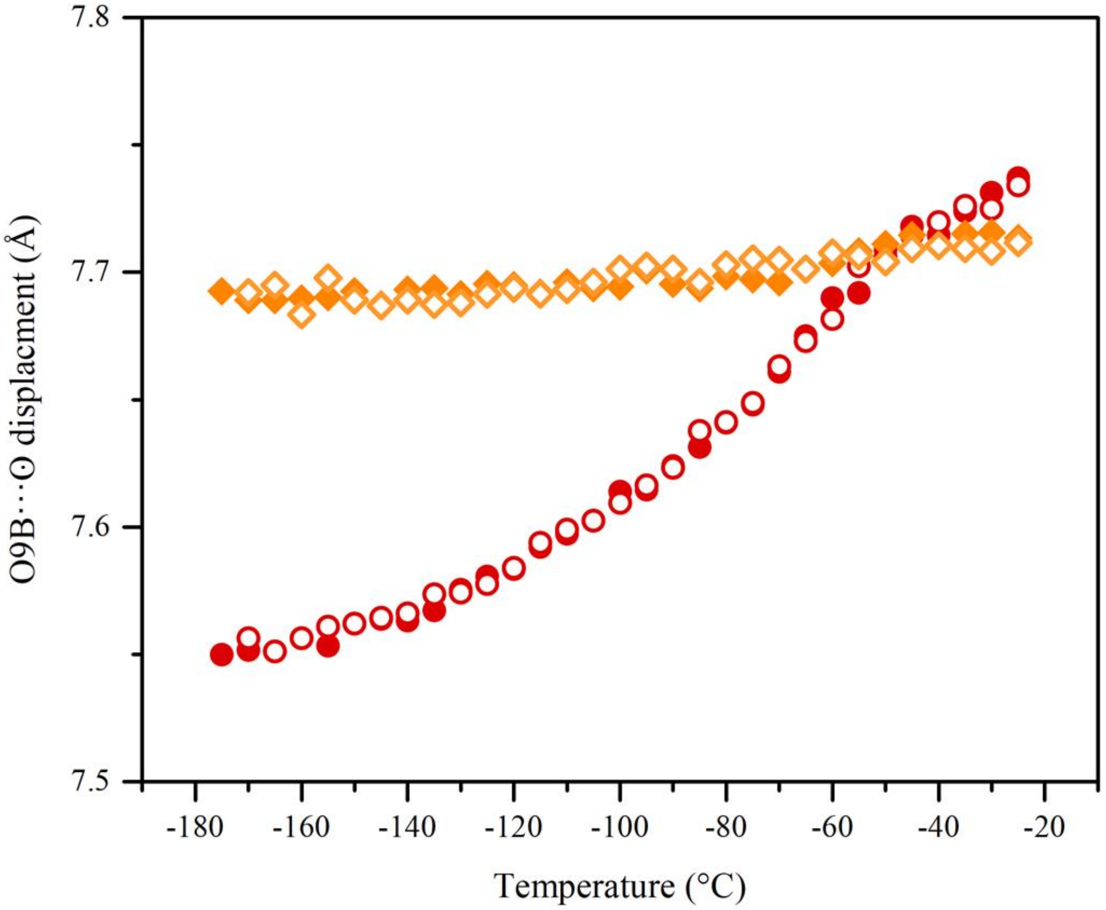

Supplementary Fig. 50. O9B···Θ$_B$ displacement plots determined for **T1-R** (red circles) and **T1-Y** (orange diamonds) during cooling (closed symbols) and heating (open symbols) between –25 and –175 °C.

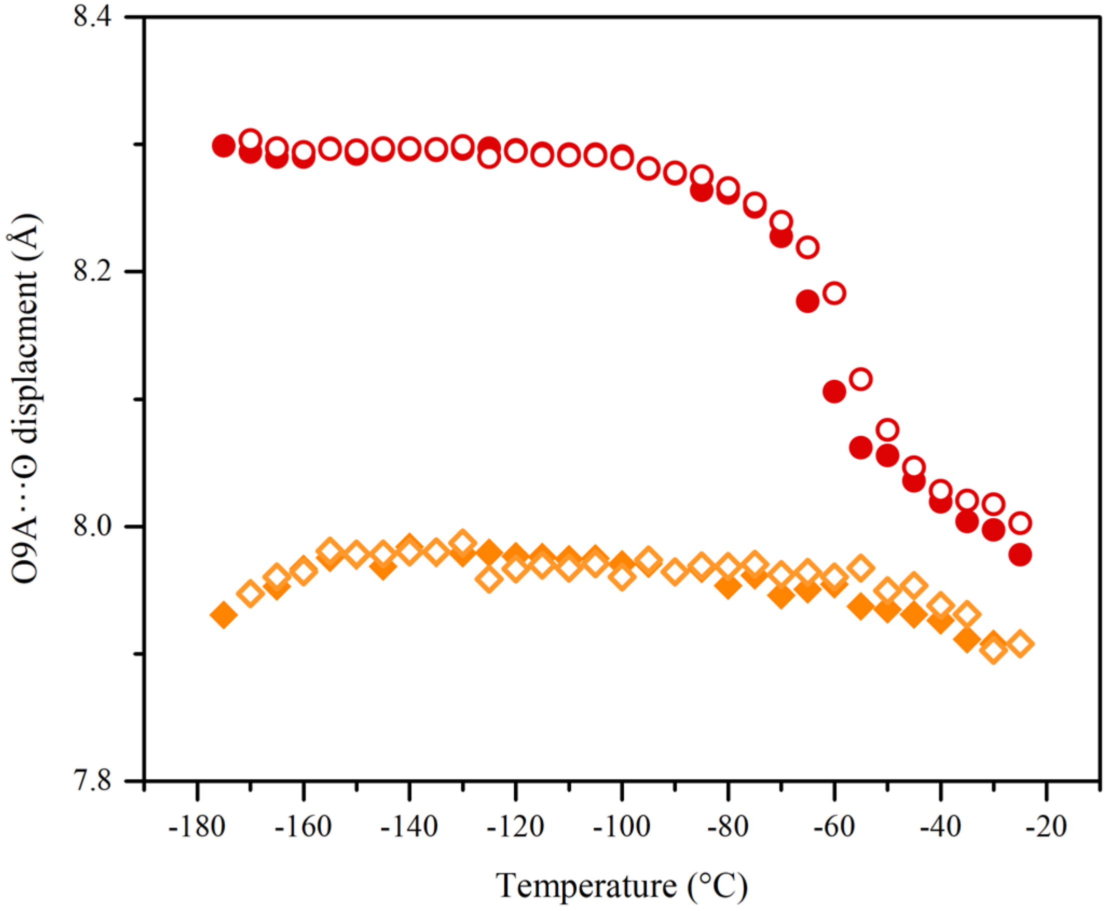

Supplementary Fig. 51. O9A···O$_A$ displacement plots determined for **T1-R** (red circles) and **T1-Y** (orange diamonds) during cooling (closed symbols) and heating (open symbols) between −25 and −175 °C.

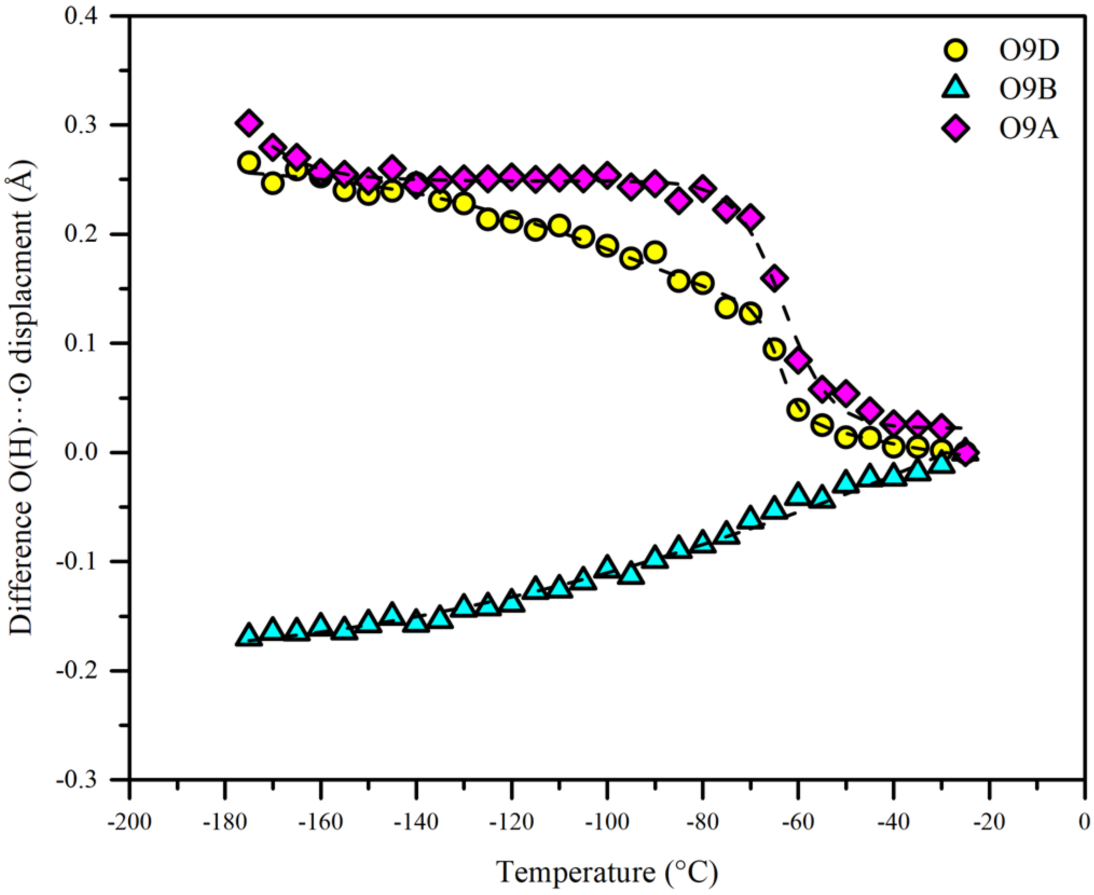

Supplementary Fig. 52. Difference O(H)···O displacement plots for atoms O9D (yellow circles), O9B (cyan) and O9A (magenta diamonds) during cooling of **T1** during from −25 to −175 °C (i.e., relative to their initial values at the starting point of −25 °C).

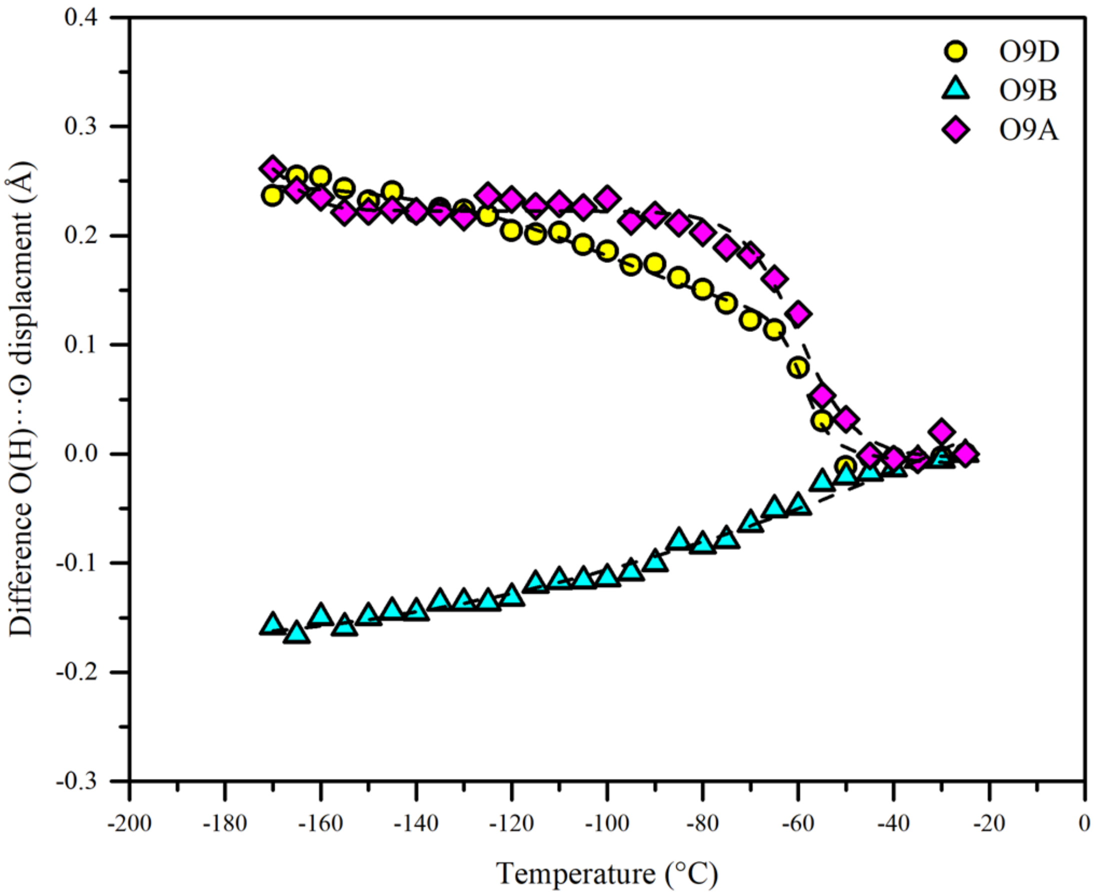

Supplementary Fig. 53. Difference O(H)···O displacement plots for atoms O9D (yellow circles), O9B (cyan) and O9A (magenta diamonds) during heating of **T1** during from –175 to –25 °C (i.e., relative to their final values at –25 °C).

Supplementary Text 12. Differential Scanning Calorimetry

The dynamic nature of water in a glass-like transition state has been investigated extensively using calorimetry in combination with X-ray diffraction analyses[85,95]. To increase the sensitivity of the measurements, a sample consisting of 4.05 mg of single crystals was placed directly onto the DSC sensor stage (Supplementary Fig. 54). The sequence for measuring heat flow as a function of temperature for **T1-R** and **T1-Y** was as follows: crystals of **T1-R** were cooled from –2 to –160 °C at a rate of 15 °C min^{-1}, equilibrated for 5 min, heated to –2 °C, followed by equilibration for 5 min. This sequence was repeated over seven cycles. The sample was then heated to 50 °C and allowed to equilibrate for 20 min to release the included water molecules. The anhydrous crystals were then subjected to the same cooling and heating cycle as used for the hydrous crystals. The thermograms for cooling (Supplementary Fig. 55) and heating (Supplementary Fig. 56) were compared between the hydrous and anhydrous samples. Relative to the anhydrous crystals, the slope for the hydrous crystals changes at approximately –50 °C during cooling, indicating that the heat capacity of **T1-R** is likely affected by a structural event due to the presence of water in the channels. The extent of the inflection decreases over subsequent cycles, which is consistent with the gradual release of water over time, even at temperatures below 0 °C.

It is important to note that all of the abovementioned DSC experiments were carried out using the same sample of crystals to investigate both the hydrous and anhydrous forms. Moreover, the crystals were not disturbed between experiments. Since the host crystal structure is, in principle, the same for the hydrous and anhydrous forms, the anhydrous crystals serve as a convenient reference (i.e. blank) for the hydrous crystals. Therefore, subtracting the thermogram of the anhydrous crystals from that for the hydrous crystals (we refer to this as a 'difference thermogram') accentuates any thermal events due to the presence of water (Supplementary Figs. 57 and 58). The difference DSC thermogram of **T1-R**, (i.e., also using **T1-Y** as a reference), enhances the subtle but noticeable inflection that is consistent with the initiation and propagation of local ordering of nanoconfined water[165,166].

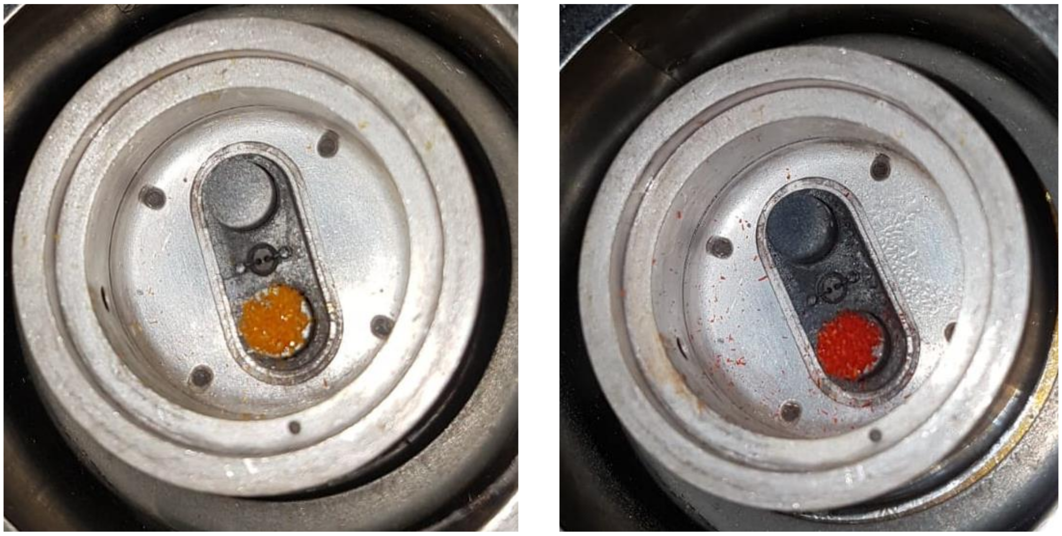

Supplementary Fig. 54. Photographs of single crystals of **T1** on the DSC sensor before (left) and after (right) hydration.

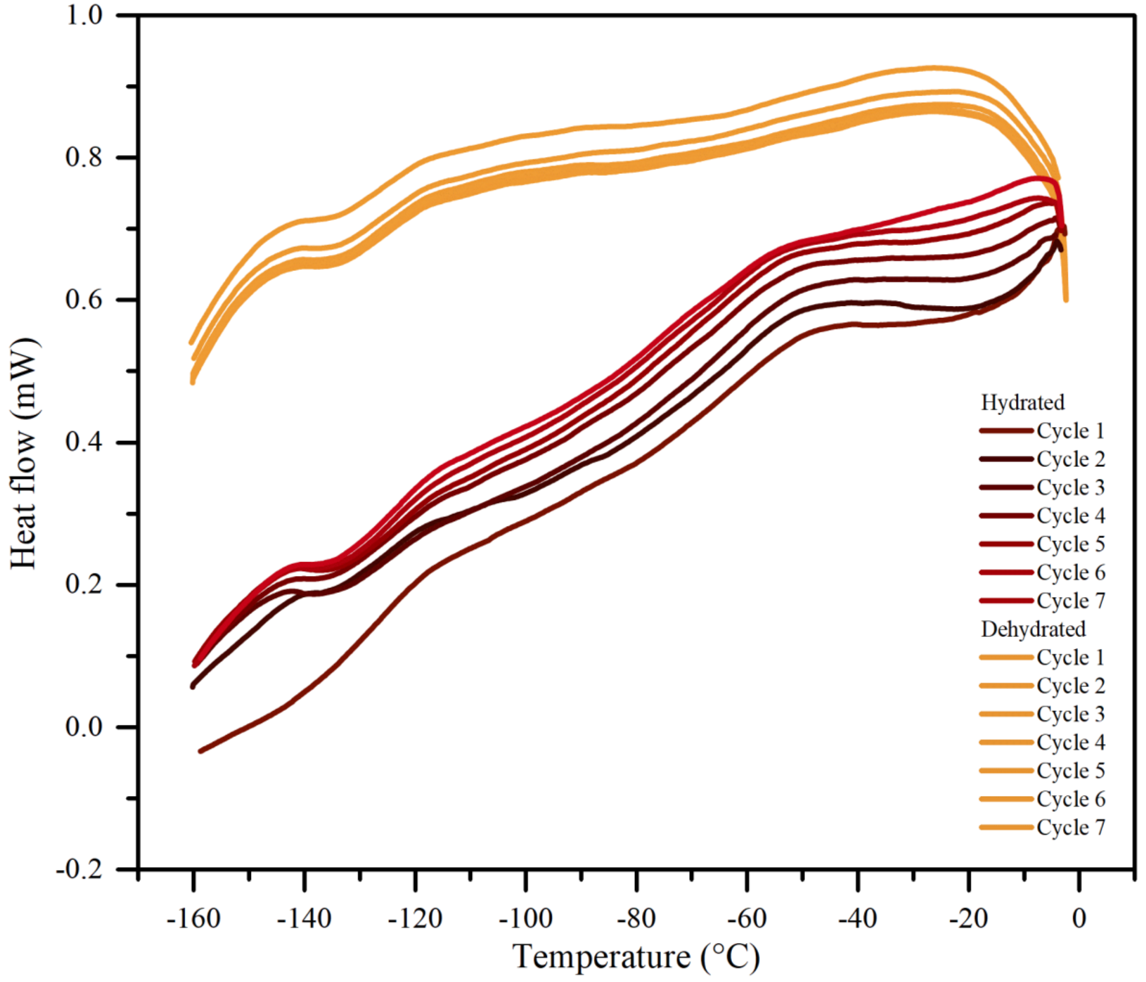

Supplementary Fig. 55. DSC thermograms of the cooling segments for crystals of **T1-Y** (yellow) and **T1-R** (red) over 7 cycles (cooling from –2 to –160 °C at 15 °C min^{-1}). Loss in the definition of the peak centred at –50 °C is likely due to the gradual loss of water during repeated cycling.

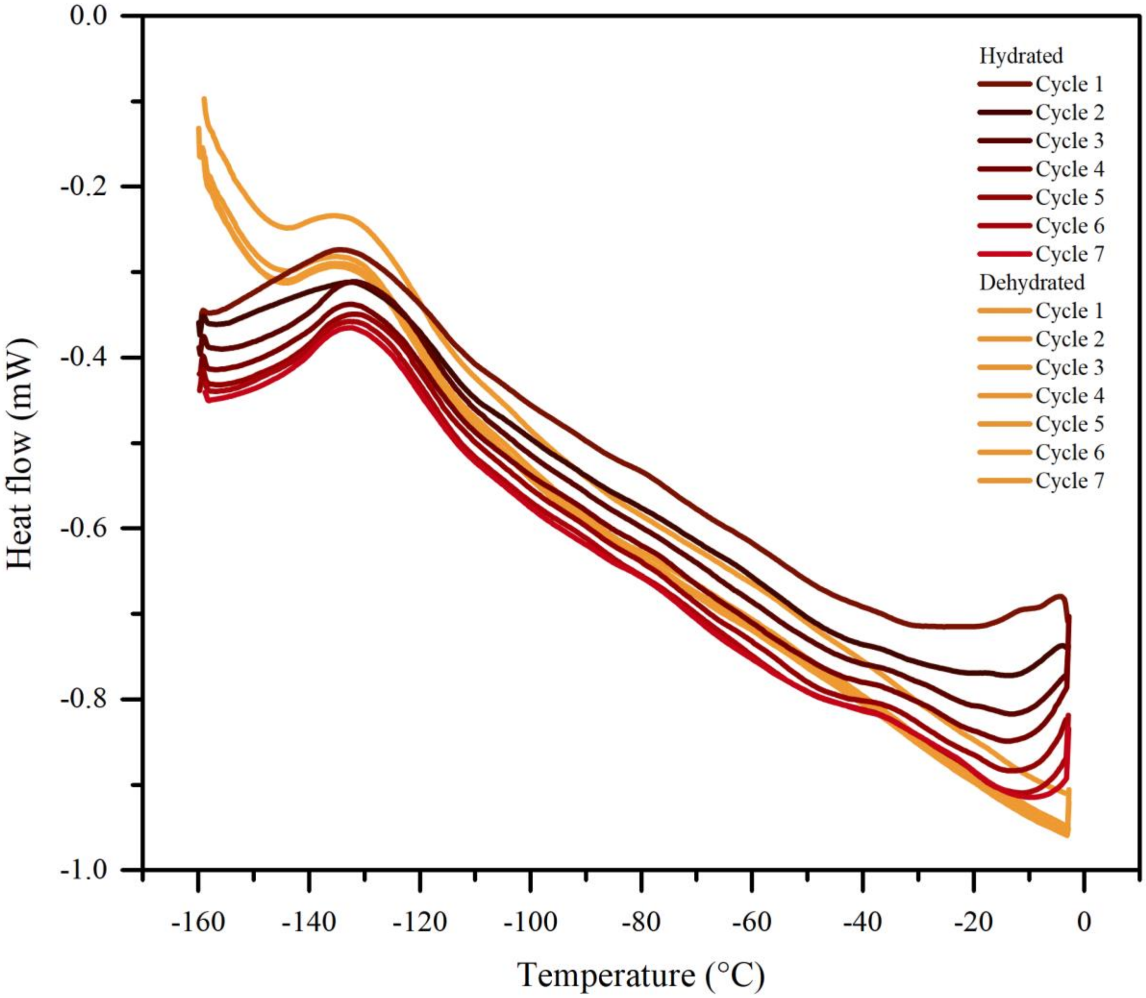

Supplementary Fig. 56. DSC thermograms of the heating segments for crystals of **T1-Y** (yellow) and **T1-R** (red) over 7 cycles (heating from –160 to –2 °C at 15 °C min^{-1}).

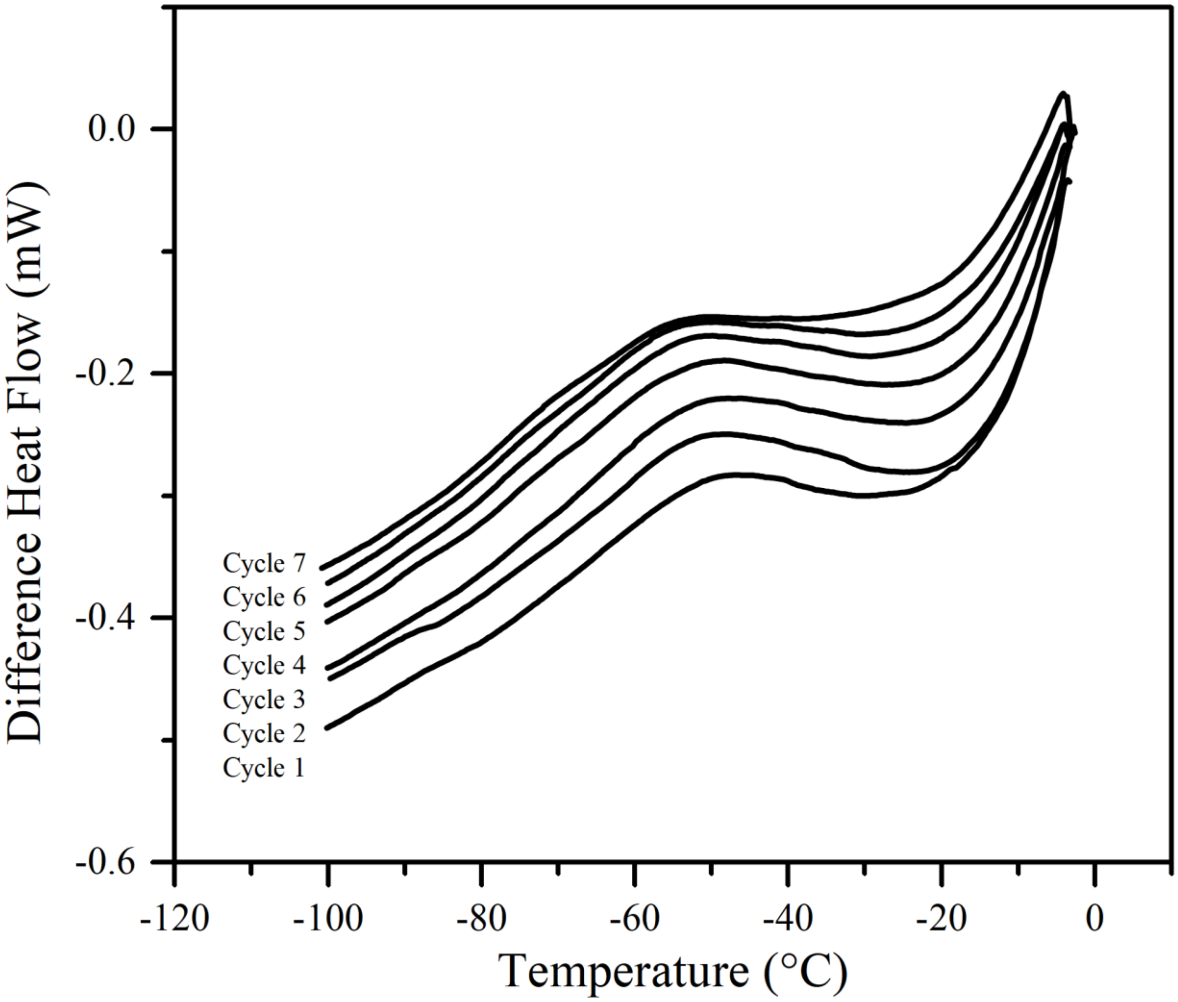

Supplementary Fig. 57. Difference DSC thermograms derived by subtracting the heat flow values for the cooling segments of measured for crystals of **T1-Y** from those of **T1-R** over 7 cycles (cooling from –2 to –160 °C at 15 °C min^{-1}).

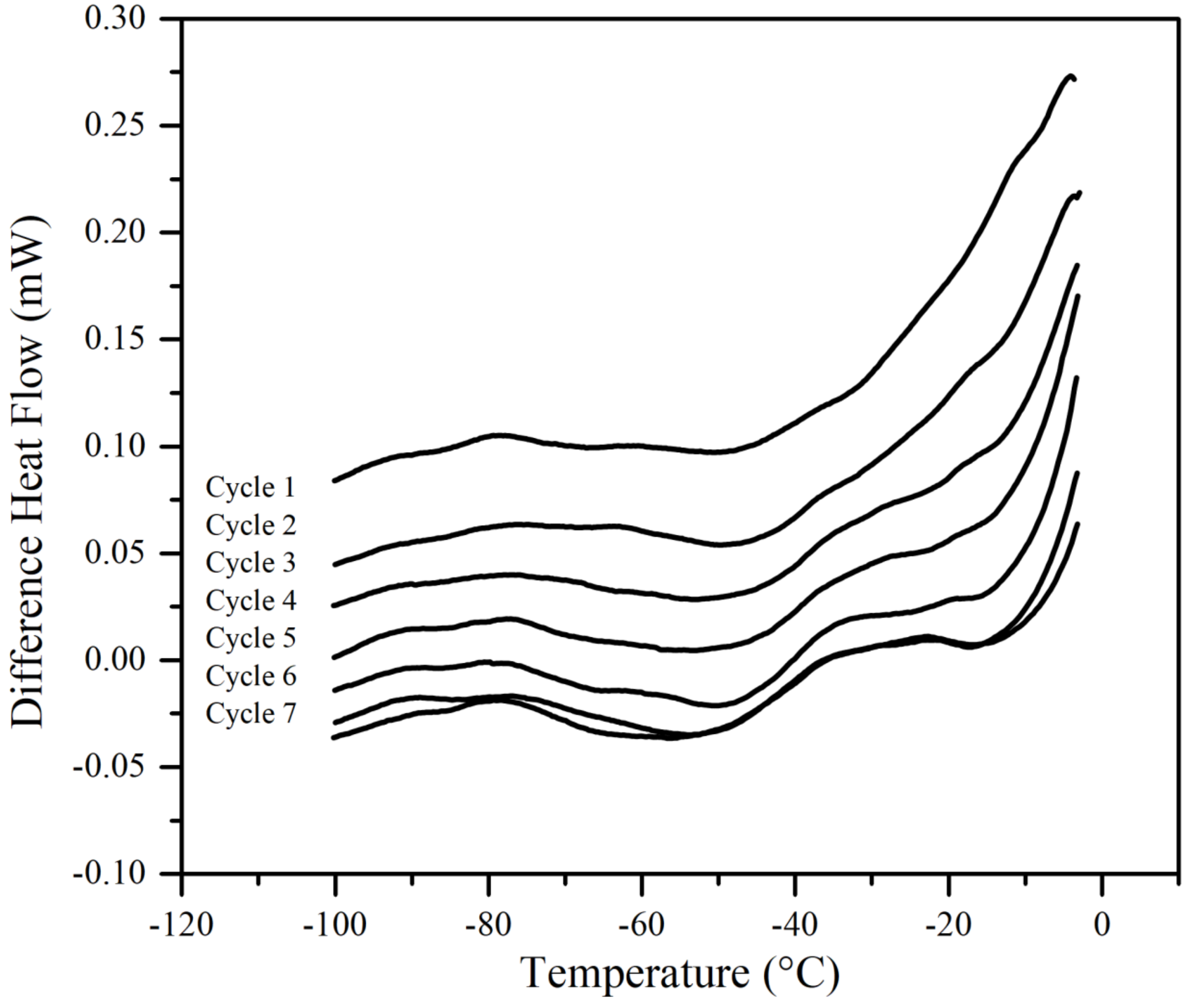

Supplementary Fig. 58. Difference DSC thermograms derived by subtracting the heat flow values for the heating segments of measured for crystals of **T1-Y** from those of **T1-R** over 7 cycles (heating from –160 to –2 °C at 15 °C min^{-1}).

Supplementary Text 13. Kinetic Analysis of Low-Temperature Water Release

Since it was not feasible to identify the minimum temperature of water loss using a trial-and-error approach, we carried out a kinetic analysis of the temperature-dependent rates of water loss (Supplementary Text 13.1) and rates of diffusion in **T1** (Supplementary Text 13.2).

Supplementary Text 13.1. Microscopic Analysis of the Temperature-Dependence of the Rate of Dehydration

The temperature-dependent rate of channel emptying by **T1** was studied by measuring the rate of evolution of the yellow boundaries during dehydration (Supplementary Fig. 59). A single crystal of **T1-Y** (272 × 63 × 83 µm^3) was glued to a glass fibre, hydrated and placed in the dry nitrogen stream at the experimental temperature. Images of the crystal were recorded at regular intervals and the values of d were noted as a function of time t over a wide temperature range (Supplementary Figs. 60 to 66). These experiments revealed that the displacement occurs linearly with time and the rate of water release R_{wr} could be determined by least squares regression of the slopes of the graphs. The average values of R_{wr} were plotted (Supplementary Fig. 67) as a function of temperature, revealing an exponential relationship.

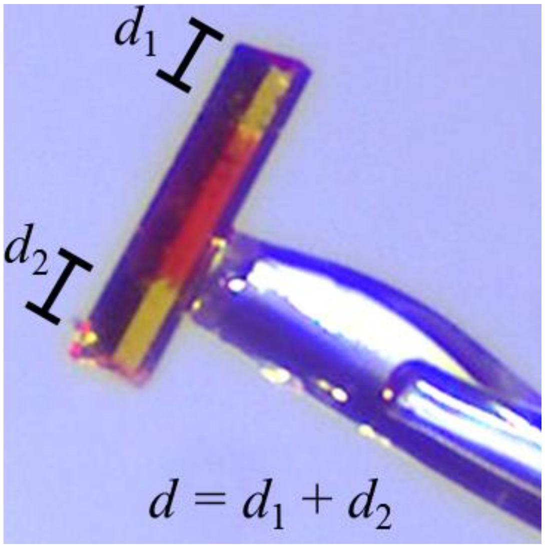

Supplementary Fig. 59. Photomicrograph of an initially red (hydrous) crystal of **T1** (glued to a glass fiber) undergoing dehydration in a stream of dry nitrogen gas at –20 °C. The extent of water release was determined by measuring the evolution of the yellow boundary with respect to time: the distances between the crystal faces and the yellow boundaries, d_1 and d_2, were measured and summed to yield a total displacement d.

It is apparent from the linearity of the *d vs t* curves that the rate of channel emptying is constant, thus implying a zero-order reaction mechanism[167]:

$$R = k_{\mathrm{wr}}[\mathrm{H_2O}]^0 = k_{\mathrm{wr}}$$

where k_{wr} is the rate constant of water release with units M s^{-1} and [H_2O] is the concentration of water in the red (hydrous) region of the crystal. The intensive rate of water release R_I can be expressed as[168]

$$R_{\mathrm{I}} = \frac{1}{V_{\mathrm{c}}}\left(-\frac{1}{\nu}\frac{dn_{\mathrm{H_2O}}}{dt}\right)$$

where V_c is the volume that water occupies in the crystal, ν is the stoichiometric coefficient and $n_{\mathrm{H_2O}}$ is number of moles of water in the crystal at any given time. Three water molecules per host formula unit are lost during pore emptying, therefore $\nu = 3$. For a crystal of **T1** with length l_c along the channel axis and cross-sectional area A_c

$$R_I = \frac{1}{l_{\mathrm{c}}A_{\mathrm{c}}}\left(-\frac{1}{3}\frac{dn_{\mathrm{H_2O}}}{dt}\right)$$

The molar quantity of water molecules that occupy the crystal during pore emptying at any given time is

$$n_{\mathrm{H_2O}} = [\mathrm{H_2O}]V_{\mathrm{c}} = [\mathrm{H_2O}]A_{\mathrm{c}}(l_{\mathrm{c}} - d)$$

which, after substitution gives

$$R_I = \frac{1}{l_{\mathrm{c}}A_{\mathrm{c}}}\left(-\frac{1}{3}\frac{d[\mathrm{H_2O}]A_{\mathrm{c}}(l_{\mathrm{c}} - d)}{dt}\right)$$

Since $[\mathrm{H_2O}]$, l_c and A_c are constant

$$R_I = \frac{[\mathrm{H_2O}]}{3l_{\mathrm{c}}}\left(\frac{d(d)}{dt}\right) = \frac{[\mathrm{H_2O}]}{3l_{\mathrm{c}}}R_{\mathrm{wr}} = \beta R_{\mathrm{wr}} = k_{\mathrm{wr}}$$

where the constant β = 198(10) nM m^{-1} for the crystal shown in Supplementary Fig. 59. If channel emptying follows Arrhenius behaviour then

$$k_{\mathrm{wr}} = Ae^{-\frac{E_{\mathrm{a,wr}}}{RT}}$$

where A is the pre-exponential factor, $E_{a,wr}$ the activation energy of water release by **T1**, and R the gas constant (8.314 J K^{-1} mol^{-1}). Taking the logarithmic form:

$$\ln k_{\mathrm{wr}} = \ln A - \frac{E_{\mathrm{a,wr}}}{R}\left(\frac{1}{T}\right)$$

and substituting for k_{wr}

$$\ln R_{\mathrm{wr}} + \ln \beta = \ln A - \frac{E_{\mathrm{a}}}{R}\left(\frac{1}{T}\right)$$

following rearrangement, R_{wr} is related to the temperature by

$$\ln R_{\mathrm{wr}} = \ln\left(\frac{A}{\beta}\right) - \frac{E_{\mathrm{a}}}{R}\left(\frac{1}{T}\right)$$

The activation energy of water release is a useful parameter to compare the ease with which channel hydrates release nanoconfined water: a lower activation energy would correspond to a higher rate of channel emptying. The activation energy of channel emptying in **T1** was determined to be $E_{a,wr}$ = 41(2) kJ mol^{-1} and the pre-exponential term $\frac{A}{\beta}$ = 13(3) m s^{-1} (Supplementary Fig. 68). It should be noted that the activation energy of pore emptying is only valid for the temperature range at which nanoconfined water can flow freely in the channels of **T1**. Once included water solidifies, the mechanism of water release would likely change, resulting in an increase in the activation energy for dehydration.

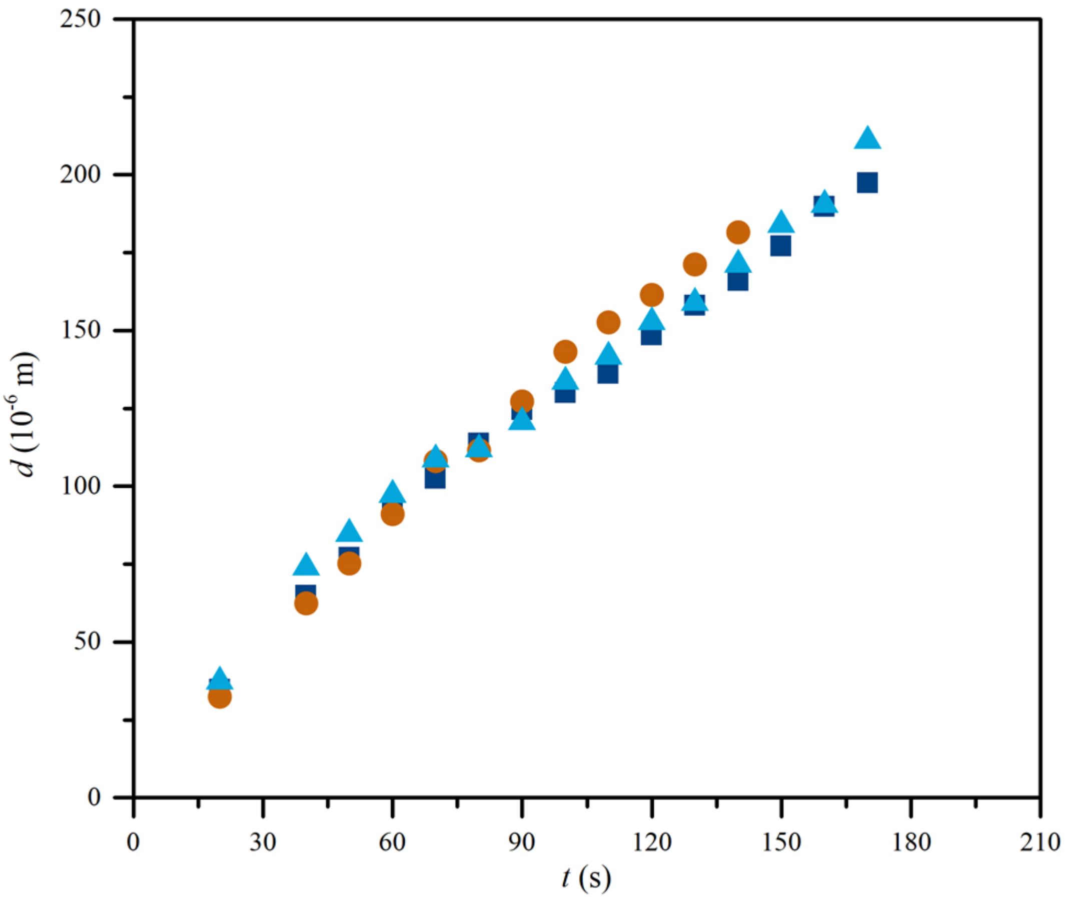

Supplementary Fig. 60. Displacement *vs* time plots determined by measuring the evolution of the yellow boundary during the release of water from the red crystal at 25 °C (in triplicate).

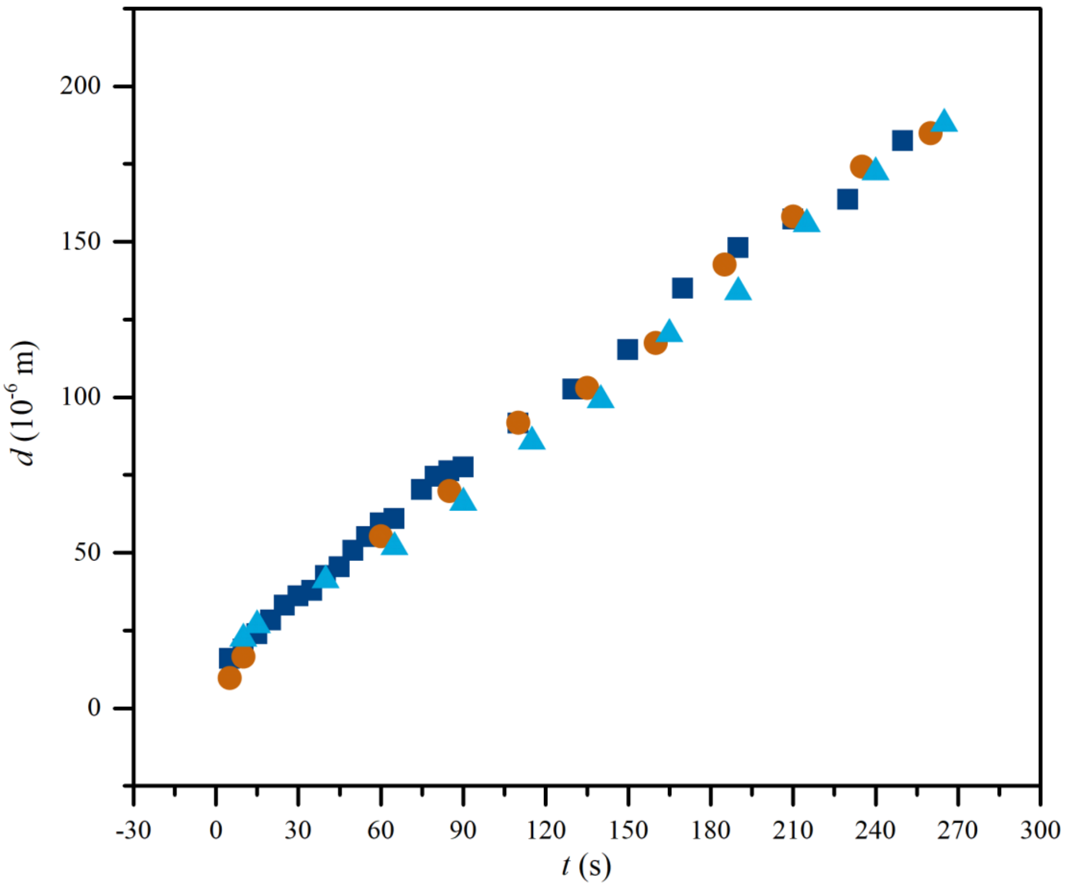

Supplementary Fig. 61. Displacement *vs* time plots determined by measuring the evolution of the yellow boundary during the release of water from the red crystal at 15 °C (in triplicate).

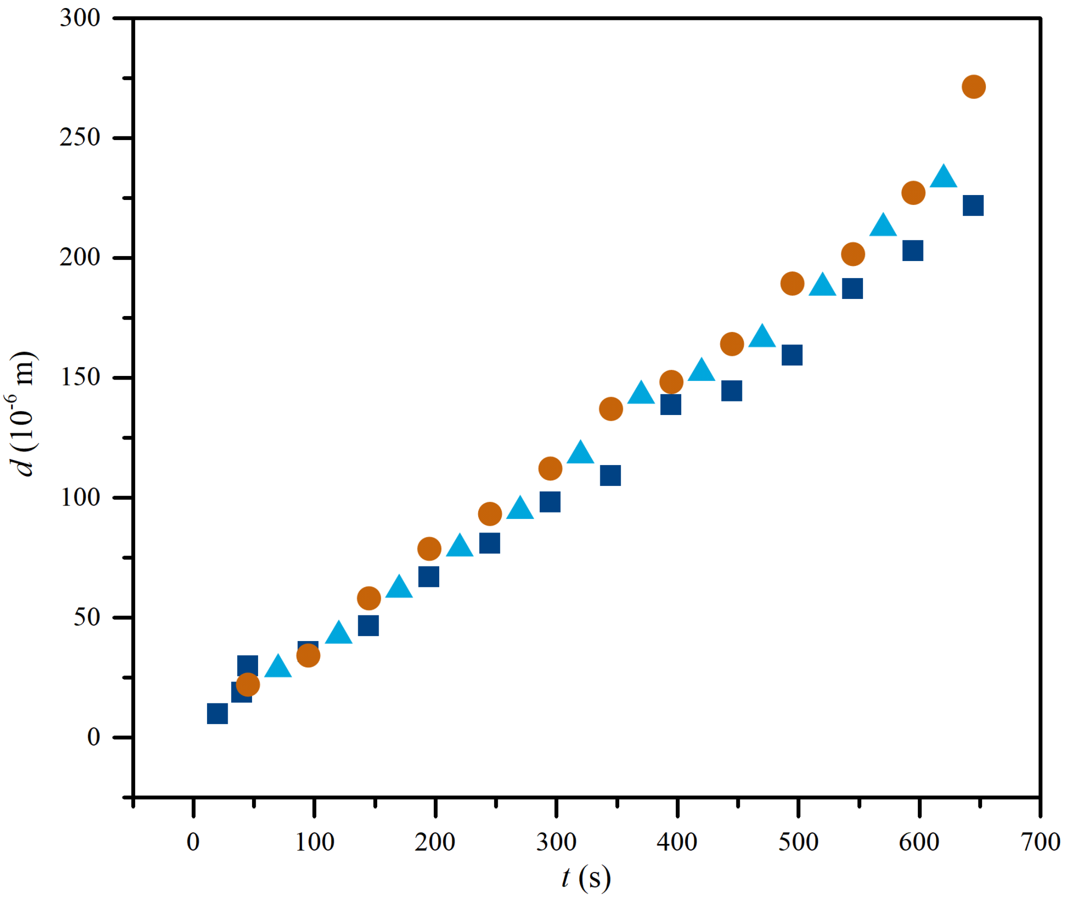

Supplementary Fig. 62. Displacement *vs* time plots determined by measuring the evolution of the yellow boundary during the release of water from the red crystal at 5 °C (in triplicate).

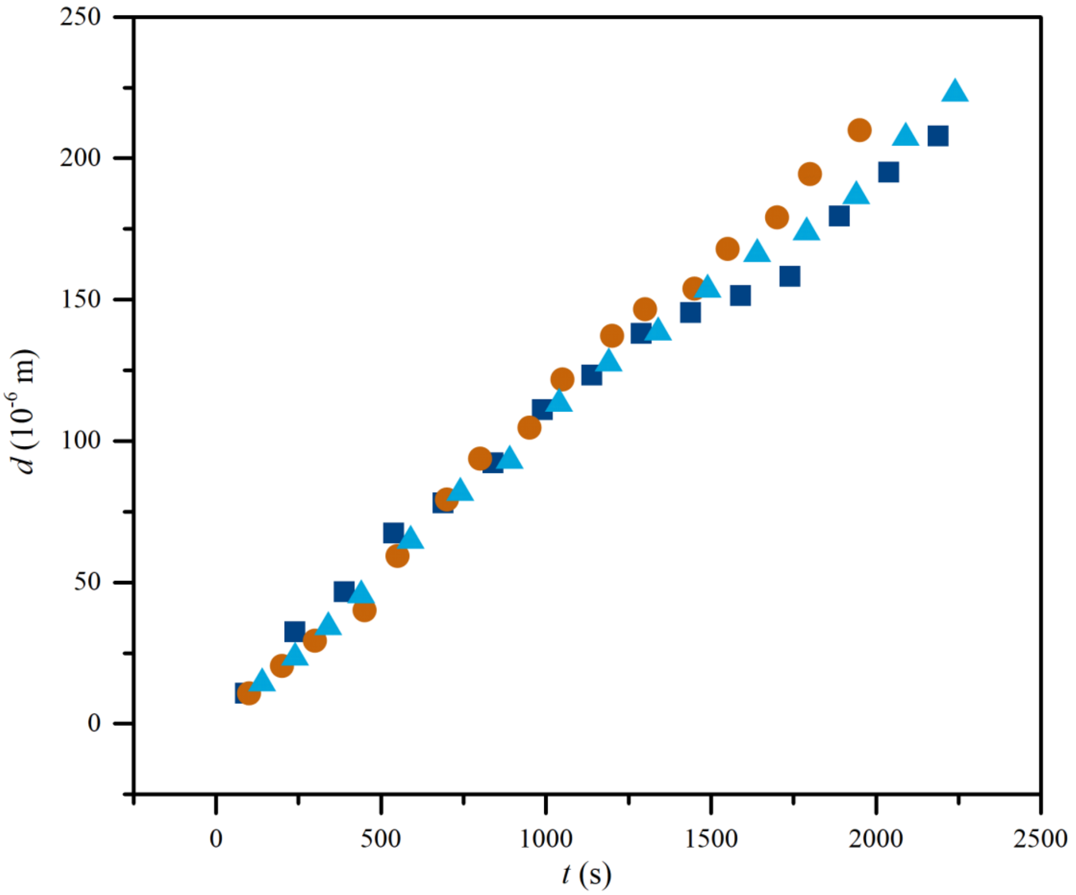

Supplementary Fig. 63. Displacement *vs* time plots determined by measuring the evolution of the yellow boundary during the release of water from the red crystal at –5 °C (in triplicate).

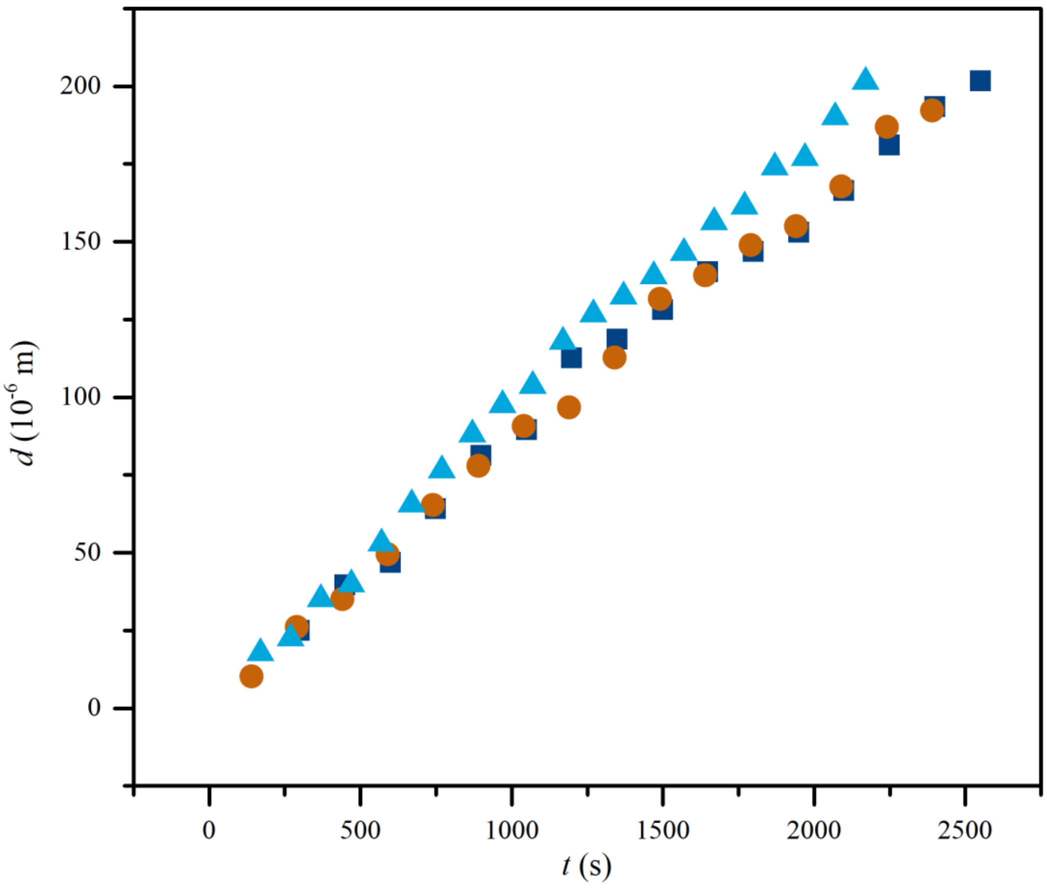

Supplementary Fig. 64. Displacement *vs* time plots determined by measuring the evolution of the yellow boundary during the release of water from the red crystal at –15 °C (in triplicate).

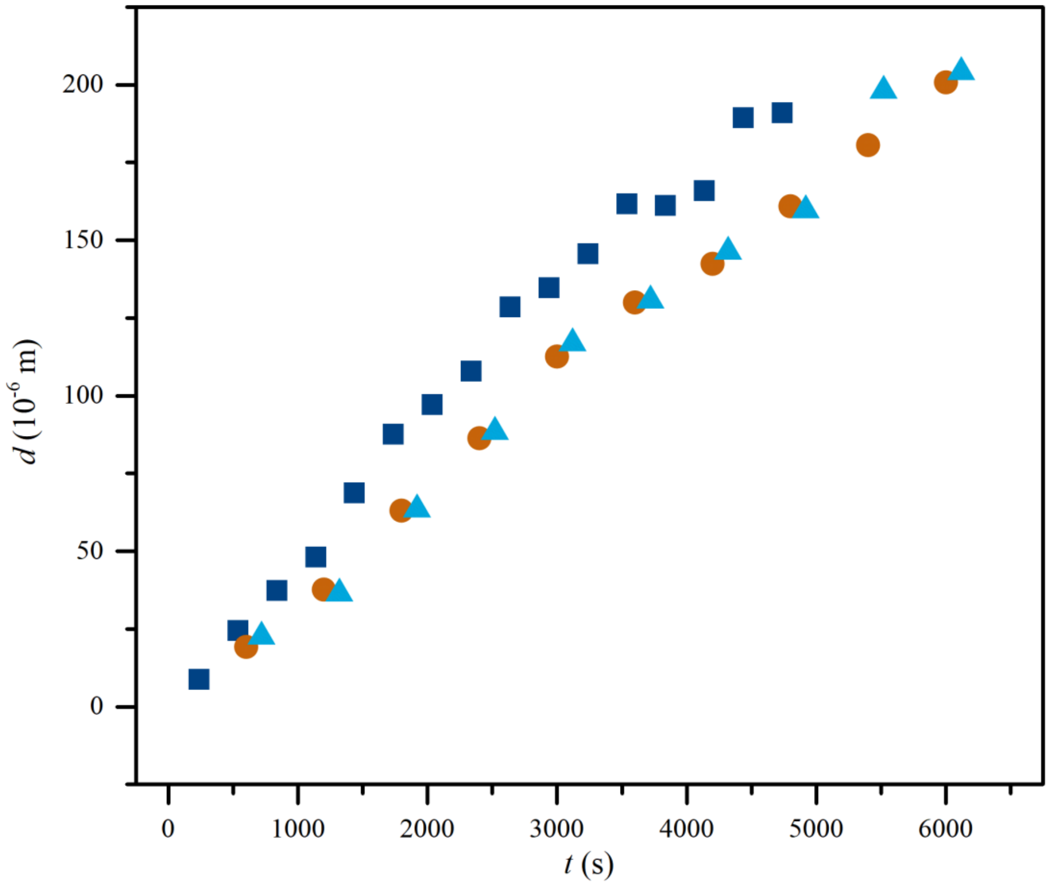

Supplementary Fig. 65. Displacement *vs* time plots determined by measuring the evolution of the yellow boundary during the release of water from the red crystal at –25 °C (in triplicate).

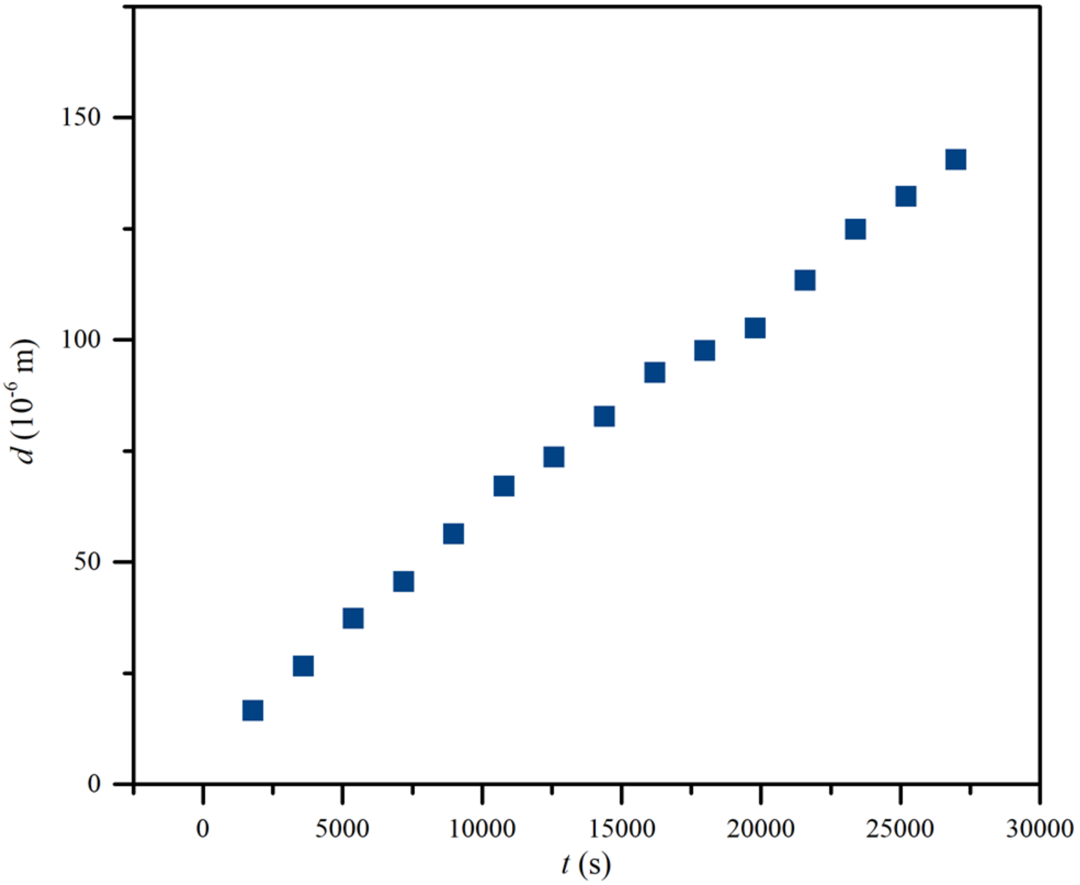

Supplementary Fig. 66. Displacement *vs* time plot determined by measuring the evolution of the yellow boundary during the release of water from the red crystal at –50 °C.

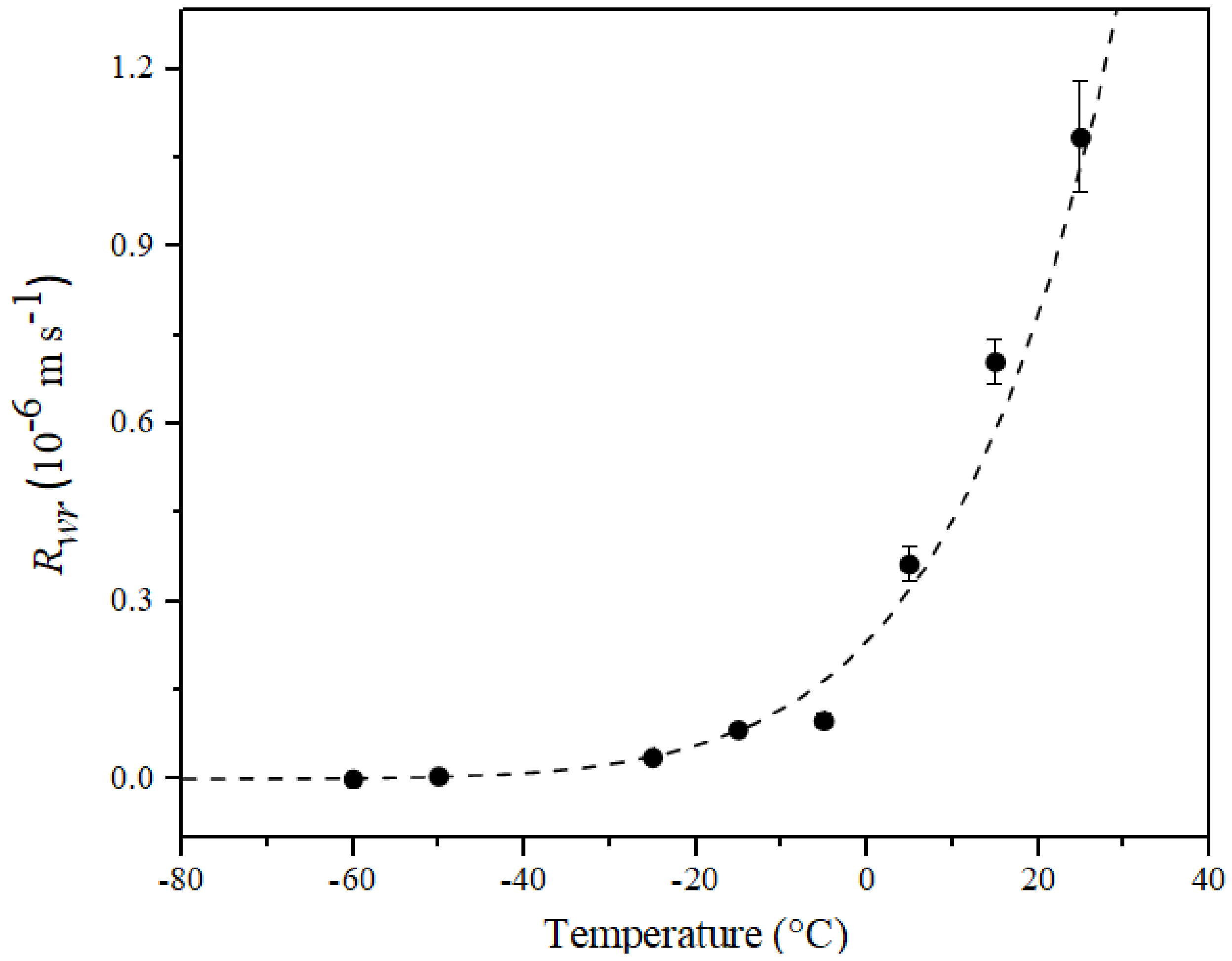

Supplementary Fig. 67 R_{wr} plotted as a function of temperature.

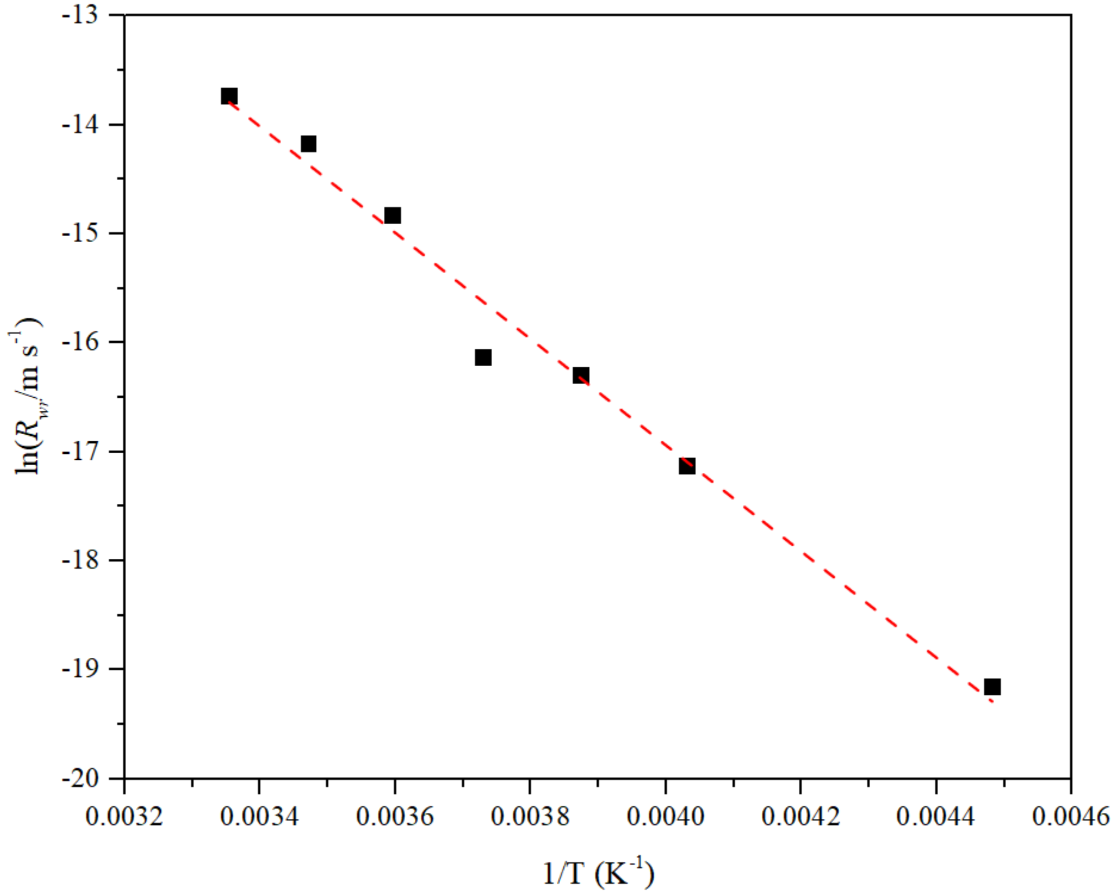

Supplementary Fig. 68. Arrhenius plot for the zero-order reaction model of water release by **T1**. The least-squares regression line of best fit is shown in red.

Supplementary Table 16. Reported dehydration temperatures and $E_{a,wr}$ for water release by hydrated materials at 1 atm and under vacuum.

Material	Dehydration Temperature (°C)	$E_{a,wr}$ (kJ mol^{-1})	Reference
1 atmosphere			
Thymine hydrate (TH) (5.0 °C/min)	54.4(7)	115−122	169
TH−AUr10	85(2)	85(2)	29
β-cyclodextrin	30–150	60	82
MIL-101Cr	55	91−111	170
Vacuum			
$\{[Mn^{II}(imH)]_2[Mo^{IV}(CN)_8]\}_n$	20–50	101(7)	171
	200	65(3)	
Silica gel (A-type) (4.0 °C/min)	72.0	35.54	172
Silica gel (B-type) (4.0 °C/min)	68.7	31.41	
Silica gel (C-type) (4.0 °C/min)	64.65	26.16	

Supplementary Text 13.2 Computational Analysis of Diffusion

The rate of diffusion within the channel of **T1** was determined using mean-square displacement analysis. Fick's first law

$$J = -D\frac{d\varphi}{dx}$$

states that molecules will flow from regions of high to low concentration. The flux J is the amount of substance that flows per unit area in a given time interval and is described by concentration ϕ and position x. Diffusion D is proportional to the squared velocity of the diffusing particles, which depends on the temperature, the viscosity of the fluid, and the size of the particles according to the Stokes–Einstein relation.

The displacement $\boldsymbol{r}(t)$ of particles i over time t is routinely analysed using the mean square displacement (MSD)[173] defined by

$$\mathrm{MSD}(t) = \langle \boldsymbol{r}^2(t)\rangle = \langle \boldsymbol{r_i}^2(t) - \boldsymbol{r_i}^2(0)\rangle$$

For a diffusing particle, the MSD becomes linear with time and the slope defines the self-diffusion coefficient D

$$D = \frac{1}{6}\lim_{t\to\infty}\langle\frac{\mathrm{MSD}(t)}{d\Delta t}\rangle$$

Least-squares fitting can be applied to estimate the slope of MSD versus t, where D is one-sixth of the slope. The results for the MSD (Supplementary Fig. 69) of water in **T1** compare well with literature values obtained for the diffusion of water through nanoporous materials (Supplementary Table 17). Overall, **T1** experiences far higher rates of diffusion, by orders of magnitude in some cases, than other solids reported.

The value of D decreases exponentially with decreasing temperature (Supplementary Fig. 70) and the slope of the curve converges to 0 m^2 s^{-1} at <–100 °C, suggesting that the diffusion of water occurs within **T1** above these temperatures.

The relationship between the diffusion coefficient and temperature T is expressed as:

$$D = D_0 e^{-\frac{E_a}{RT}}$$

where D_0 is the pre-exponential factor, E_a the activation energy of diffusion of water within **T1**, and R the gas constant[174]. These parameters can be obtained by considering the logarithmic form:

$$\ln D = \ln D_0 - \frac{E_a}{R}\left(\frac{1}{T}\right)$$

Supplementary Fig. 71 shows the Arrhenius plot of the MSD data in this work, with an activation energy of diffusion, E_a = 15(1) kJ mol^{-1} and D_0 = 5.7(1)×10^{-6} m^2 s^{-1}.

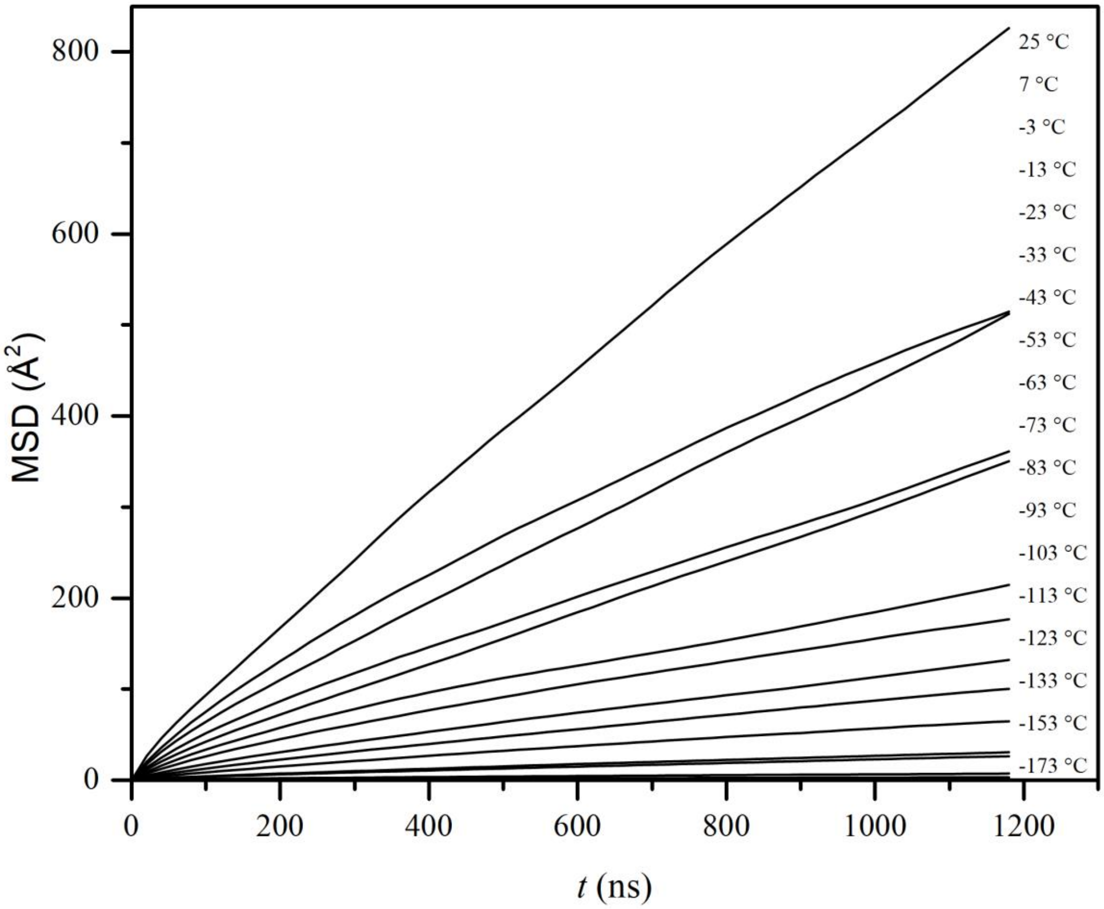

Supplementary Fig. 69. Plots of MSD *vs* time**,** determined from MD simulations of water in a single channel of **T1** between –173 and 25 °C.

Supplementary Table 17. Reported diffusion coefficients for water through nanoporous materials.

Diffusion medium	Pore diameter (nm)	Temperature (°C)	D (10^{-9} m^2 s^{-1})	Reference
Bulk water		27	2.6	34
Single walled nanotubes	1.1 – 1.4	–33	1–3 × 10^{-5}	175
		27	1–3	
Ice		–21	1.00(6)	35
		–31	0.77(4)	
		–41	0.55(3)	
		–52.5	0.35(2)	
Graphene oxide	0.8 – 1.1	27	0.1–0.3	176
Silica nanopores	2	27	1.55	177
	3	27	1.84	
	4	27	2.16	

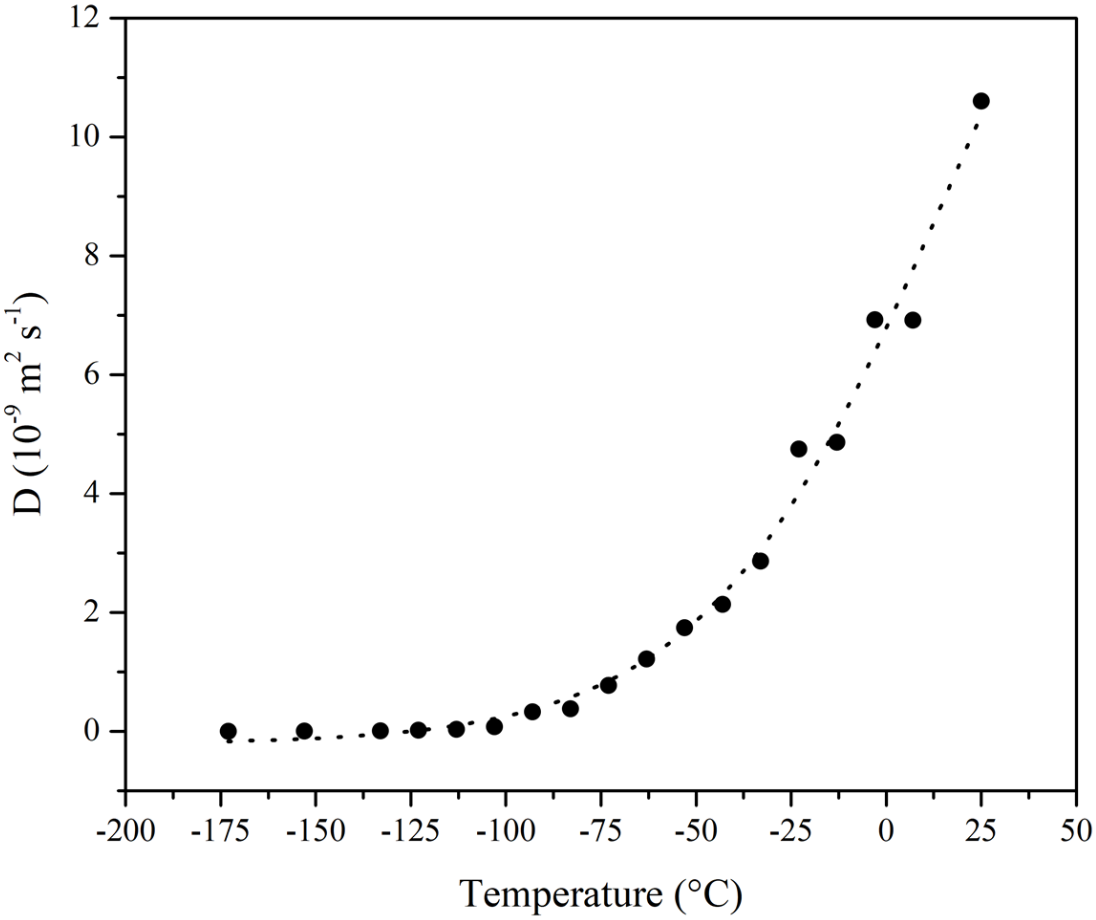

Supplementary Fig. 70. Plot of *D vs* temperature – determined from MSD analysis of water molecules in a single channel of **T1** obtained from molecular dynamics simulations carried out between –173 and 25 °C.

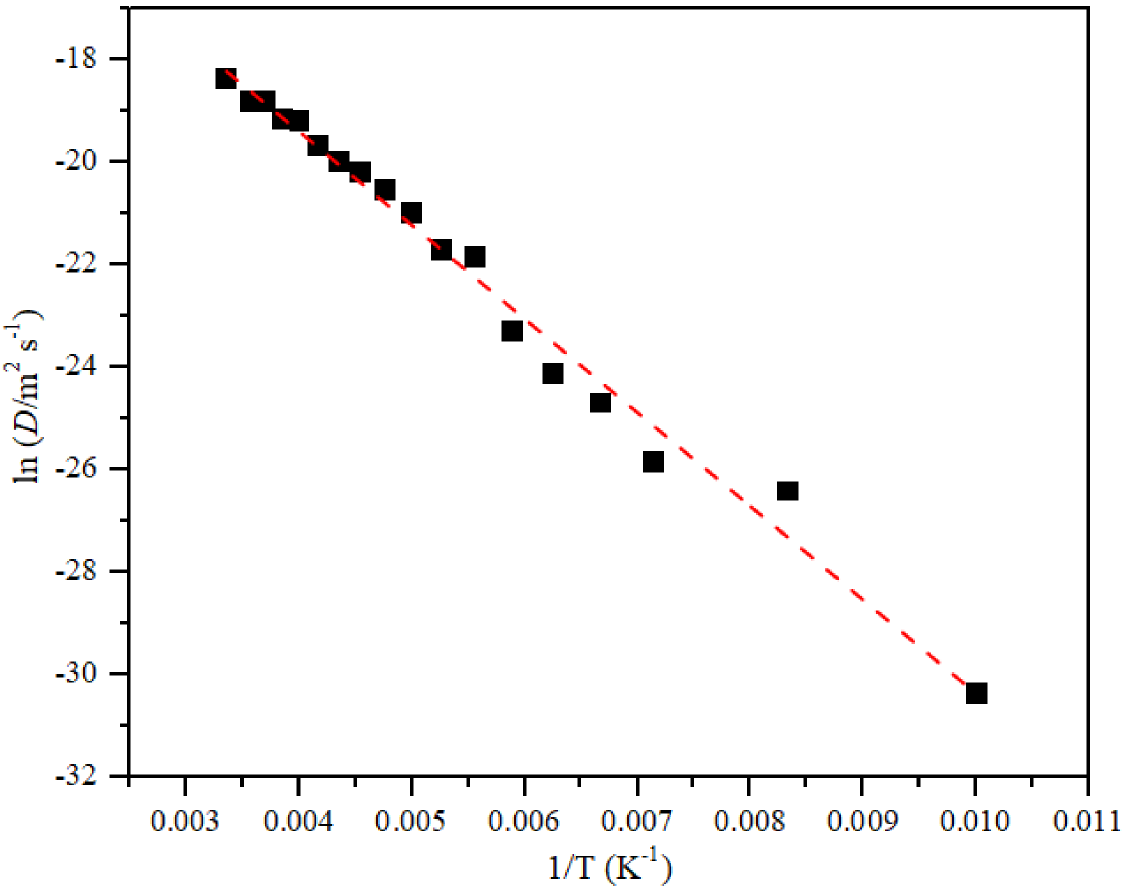

Supplementary Fig. 71. Plot of ln *D vs* 1/*T* derived from MSD data. The least-squares regression line of best fit is shown in red.

Supplementary Text 14. Commentary on Materials That May Possess a Low-Temperature T_{on}.

A cursory survey of the recent literature on the dehydration of different classes of crystal hydrate reveals additional examples of systems with sub-ambient dehydration kinetics, possibly also with sub-zero °C values of T_{on}. However, these parameters were not reported. An example from each class is described in brief below.

A Molecular Crystal Comprising a Pt Complex[27]

Bryant *et al.* reported an organoplatinum crystal that exhibits a rapid (sub-second) and reversible vapochromic response from yellow to red upon hydration at room temperature. Their study focused on the speed and the mechanism of the colour change, which they rationalised using the crystal structures of the hydrous (red) and anhydrous (yellow) crystals. They were able to model the included water molecules in what they described as channels. However, our own analysis indicates that the water molecules are situated in isolated pockets, with no water-water interactions. Although the crystal structures of the hydrous and anhydrous forms are similar, the anhydrous form is non-porous. It may thus be argued that the anhydrous form is not an isomorphic dehydrate of the hydrous form. Since the focus of the article was to elucidate the origin of the vapochromic colour change, the authors did not determine kinetic parameters or the value of T_{on} for dehydration. Moreover, DVS studies were not carried out; the %RH at which uptake occurs is thus not known, and it is also not known if the observed water sorption experiments involved quantitative uptake of water.

A Molecular Solid Comprising a Hydrous API[2]

Kennedy *et al*. reported the crystal structure of the non-stoichiometric channel hydrate of Cephalexin, which Stephenson *et al*.[28] had shown loses water at RT. This is of interest as Kennedy *et al.* described their attempts to maintain the water within the channel by cooling the crystals to 277 K, which on the basis of our study is likely not a low enough temperature. This would explain their anomalous results.

A Metal-Organic Framework with T_{on} < RT[37]

Hanikel *et al*. evaluated the uptake mechanism of water into a permanently porous metal-organic framework (MOF-303). This was done by modelling the positions of water determined from 20 crystal structure "snapshots" measured during "dehydration". More specifically, a single crystal was hydrated, rapidly cooled to −173 °C under a dry nitrogen stream, followed by rapid heating to 27 °C at a rate of 360 °C min^{-1}. This "gentle", heating-induced desorption procedure had to be carried out since the crystals cracked during rapid adsorption and desorption that occurred during attempts to seal the crystal in a capillary under a high RH environment. They assumed that mechanisms for water uptake and release in MOF-303 are the same since there is minimal hysteresis in the desorption isotherm. Interestingly, the authors noted that rapid desorption occurred between −23 and −13 °C, indicating T_{on} < 0 °C. Although part of the focus of the work was to improve dehydration kinetics, the kinetic parameters were not determined and the authors did not comment on the sub-zero temperature water release apart from noticing that the crystal quality was substantially diminished over this temperature range. They also found that the hydrous crystal structure underwent substantial unit cell changes between −173 °C and 27 °C whereas the anhydrous crystal structure was remained relatively unchanged over the same temperature range. They concluded that the structural change was due to the presence of included water.

By varying the ligand ratio using a multivariate approach, the authors were able to tune the position of the step in the sorption isotherm. This resulted in a corresponding shift in the dehydration temperature as evidenced by TGA. They concluded that their multivariate approach could be used to design water sorbents with "ultralow" desorption temperatures, as demonstrated with isobaric desorption curves. Moreover, they concluded that lower temperature desorption allowed for more water harvesting cycles per day, because less time would be taken for heating to higher temperatures followed by necessary subsequent cooling.

A Covalent Organic Framework with T_{on} < RT[178]

Nguyen *et al*. reported a permanently porous covalent organic framework that undergoes a humidity-triggered water exchange at RT. The uptake and release of water was reported at 10, 25 and 40 °C demonstrating that T_{on} is lower than 10 °C. They performed 300 adsorption-desorption cycles between 30 and 35 °C, at 40% and 30% RH, respectively. Although, the water release temperatures were thought to be "ultralow", T_{on} and kinetic parameters were not reported.

Freezing of Nanoconfined Water in a Channel Hydrate

Tadokoro *et al*.[112] reported a permanently porous, hydrogen bonded metal complex ($\{[Co^{III}(H_2bim)_3](TMA)\}_n$) with a 1 nm channel. The hydrate lost water easily at RT as evidenced by TGA. The vitrification of nanoconfined water during cooling was identified by an exothermic peak in the DSC thermogram with an onset temperature of −38.8 °C. The authors aimed to establish the structure of water at temperatures both above and below the solidification event. The positions of water molecules in the hydrate could not be modelled from SC-XRD structures measured at room temperature as the difference electron density was too diffuse. However, the largest electron density peaks were located near the functional groups along the channel walls. Repeating the experiment at −75 °C afforded a model that contained higher difference electron density peaks throughout the channel, in addition to that found along the channel walls. The structure modelled at this temperature was incommensurate with that of the host.

In a follow-up study, Tadokoro *et al*.[95] compared the temperature-dependent properties of the Cobalt containing channel hydrate with a Ruthenium analogue ($\{[Ru^{III}(H_2bim)_3](TMA)\}_n$). Once again, they observed a freezing event at low temperature and a similar lack of long-range order of the nanoconfined water. The density of the included water was lower than that for hexagonal ice and the authors concluded that water existed in a "supercooled state". Nevertheless, the authors did not link their observations to low-temperature dehydration, and therefore did not report T_{on} or kinetic parameters.

Based on the results described above, we believe that further investigation of the water uptake/release properties of these and other compounds at sub-glacial temperatures is warranted.

Captions for Supplementary Videos

Supplementary Video 1

A single crystal of **T1-Y (**297 × 37 × 37 µm^3) transitioning to red (hydrous) upon exposure to water vapor from a water-soaked ring of cotton wool at 25 °C.

Supplementary Video 2

A single crystal of **T1-R (**297 × 37 × 37 µm^3) transitioning to yellow (anhydrous) upon exposure to a flow of dry air at 25 °C.

Supplementary Video 3

Two single crystals of **T1-Y** (left, 310 × 85 × 85 µm^3; right, 171 × 122 × 122 µm^3), initially equilibrated at 53% RH, adsorbing water a 58% RH.

Supplementary Video 4

A single crystal of **T1-R (**272 × 63 × 83 µm^3) enveloped in a dry nitrogen stream at −60 °C. Full conversion from red to yellow occurred over 70 h.

Supplementary Video 5

A single crystal of **T1-R (**272 × 63 × 83 µm^3) enveloped in a dry nitrogen stream at −70 °C. The crystals remained red for 5 days, after which the experiment was terminated.

Supplementary Video 6

A single crystal of **T1-Y (**235 × 54 × 54 µm^3) with one of its channel faces coated with 5 min epoxy turning red after exposure to water vapor from a water-soaked ring of cotton wool at 25 °C. Uptake of water only occurred at the exposed face and the reverse was observed upon exposure to a flow of dry air at 25 °C.

Supplementary Video 7

A single crystal of **T1-R (**250 × 55 × 55 µm^3) enveloped in a dry stream of nitrogen gas at −25 °C. The (001) and ($00\bar{1}$) faces were coated with 5 min epoxy to prevent water loss.

Supplementary Video 8

VT-SCXRD model projections along the channel axis [$00\bar{1}$] of **T1-R** (left) and **T1-Y** (right) crystals during cooling from −25 to −125 °C.

Supplementary Video 9

VT-SCXRD perspective view along [210] showing the evolution of difference electron density in **T1-R** during cooling from −30 to −125 °C. Host molecules in the foreground have been omitted for clarity (see Supplementary Fig. 4).

Supplementary Video 10

VT-SCXRD projection along the channel axis [$00\bar{1}$] of **T1-R** (left) and **T1-Y** (right) crystals during heating from −125 to −25 °C.

Supplementary Video 11

VT-SCXRD perspective view along [210] showing the devolution of difference electron density in **T1-R** during heating from −115 to −30 °C. Host molecules in the foreground have been omitted for clarity (see Supplementary Fig. 4).

References

79. Piotrowska, R. *et al.* Mechanistic insights of evaporation-induced actuation in supramolecular crystals. *Nat. Mater.* **20**, 403–409 (2021).

80. Ohata, Y., Kouchi, T., Sugaya, T., Kamebuchi, H. & Tadokoro, M. Dynamic Water Nanotube Cluster Stabilized in Molecule-Based Hydrophilic Nanoporous Crystal with New Organic Spacers. *Bull. Chem. Soc. Jpn.* **92**, 655–660 (2019).

81. Ohata, Y. *et al.* Slow Dynamics of Premelting Water Molecules Confined in a Hydrophilic Nanoporous Space. *ChemistrySelect* **4**, 6627–6633 (2019).

82. Pereva, S., Nikolova, V., Angelova, S., Spassov, T. & Dudev, T. Water inside β-cyclodextrin cavity: amount, stability and mechanism of binding. *Beilstein J. Org. Chem.* **15**, 1592–1600 (2019).

83. Bhatt, P. M. *et al.* A Fine-Tuned Fluorinated MOF Addresses the Needs for Trace CO_2 Removal and Air Capture Using Physisorption. *J. Am. Chem. Soc.* **138**, 9301–9307 (2016).

84. Yamada, S., Sako, N., Yamada, K., Deguchi, K. & Shimizu, T. Porous organic hydrate crystals: Structure and dynamic behaviour of water clusters. *CrystEngComm* **17**, 5629–5633 (2015).

85. Tadokoro, M. *et al.* Transpiration of Water Molecules through Molecule-Based Porous Crystals with One-Dimensional Nanochannels. *Bull. Chem. Soc. Jpn.* **88**, 1707–1715 (2015).

86. Kraft, P. E., Weingartz, L. E. & LaDuca, R. L. Substituent effects in 4-connected zinc isophthalate coordination polymers with a pyridylnicotinamide ligand: From dimer-based layers to ribbons with embedded infinite water molecule chains. *Inorganica Chim. Acta* **432**, 283–288 (2015).

87. Matsui, H., Suzuki, Y., Fukumochi, H. & Tadokoro, M. Defect Dynamics of the Dipole Ordered Water Chain in a Polar Nanochannel. *J. Phys. Soc. Japan* **83**, 054708 (2014).

88. Stokes, S. P., Seaton, C. C., Eccles, K. S., Maguire, A. R. & Lawrence, S. E. Insight into the Mechanism of Formation of Channel Hydrates via Templating. *Cryst. Growth Des.* **14**, 1158–1166 (2014).

89. Tseng, T.-W., Luo, T.-T. & Lu, K.-H. Impeller-like dodecameric water clusters in metal–organic nanotubes. *CrystEngComm* **16**, 5516–5519 (2014).

90. Tadokoro, M., Ohata, Y., Shimazaki, Y., Isoda, K. & Sugaya, T. Pre-Melting Structure Transformation of Water Clusters in Nanoporous Molecular Crystals. *ChemPhysChem* **13**, 3267–3270 (2012).

91. Watanabe, K., Tadokoto, M. & Oguni, M. Thermal Characteristics of Channel Water Confined in Nanopores with Crystalline Pore-Wall Structure in $[M(H_2bim)_3](TMA)\cdot nH_2O$. *J. Phys. Chem. C* **116**, 11768–11775 (2012).

92. Matsui, H. & Tadokoro, M. Eigen-like hydrated protons traveling with a local distortion through the water nanotube in new molecular porous crystals $\{[M\mathrm{III}(H_2bim)_3](TMA)\cdot 20H_2O\}n$ (M = Co, Rh, Ru). *J. Chem. Phys.* **137**, 144503 (2012).

93. Amorín, M., Llamas-Saiz, A. L., Castedo, L. & Granja, J. R. Three-Dimensional Water Channel Embedded in an α,γ-Cyclic Octapeptide-Derived Organic Porous Material. *Cryst. Growth Des.* **11**, 3351–3357 (2011).

94. Luo, G.-G. *et al.* A Discrete Spirocyclic (H2O)9 Cluster and 1D Novel Water Chain with Tetrameric and Octameric Clusters in Cationic Hosts. *Cryst. Growth Des.* **11**, 1948–1956 (2011).

95. Tadokoro, M., Iida, C., Saitoh, T., Suda, T. & Miyazato, Y. One-dimensional Tube-like $\{5^{12}6^2\}_n$ Water Clusters Stabilized in a Molecular Nanoporous Framework. *Chem. Lett.* **39**, 186–187 (2010).

96. Tadokoro, M. *et al.* Anomalous Water Molecules and Mechanistic Effects of Water Nanotube Clusters Confined to Molecular Porous Crystals. *J. Phys. Chem. B* **114**, 2091–2099 (2010).

97. Hu, J.-S. *et al.* Syntheses, Structures, and Photoluminescence of Five New Metal−Organic Frameworks Based on Flexible Tetrapyridines and Aromatic Polycarboxylate Acids. *Cryst. Growth Des.* **10**, 2676–2684 (2010).

98. Watanabe, K., Oguni, M., Tadokoro, M. & Kobayashi, C. Ordering and Freezing-in Phenomena of Nanochannel Water in Crystalline Organic/Inorganic Self-Assembled Complex $[Cr(H_2bim)_3](TMA)\cdot 23.5H_2O$. *J. Phys. Chem. B* **113**, 14323–14328 (2009).

99. Nakabayashi, K. & Ohkoshi, S. Monometallic Lanthanoid Assembly Showing Ferromagnetism with a Curie Temperature of 11 K. *Inorg. Chem.* **48**, 8647–8649 (2009).

100. Li, J.-Y., Sun, D.-F., Hao, A.-Y., Sun, H.-Y. & Shen, J. Crystal structure of a new cyclomaltoheptaose hydrate: β-cyclodextrin$\cdot 7.5H_2O$. *Carbohydr. Res.* **345**, 685–688 (2010).

101. Huang, K.-L., Liu, X., Chen, X. & Wang, D.-Q. Spontaneous Assembly of 6^3 Topological Metal−Organic Nanotubes with Distinct Asymmetric Subunits for the Construction of Hydrophilic Intertube Channels Encapsulating Rare Helical Water-Chains. *Cryst. Growth Des.* **9**, 1646–1650 (2009).

102. Quan, Y.-P. *et al.* Novel lanthanide coordination polymers based on bis-tridentate chelator pyrazine-2,3,5,6-tetracarboxylate with nano-channels and water clusters. *CrystEngComm* **11**, 1679 (2009).

103. Xian, H.-D., Li, H.-Q., Shi, X., Liu, J.-F. & Zhao, G.-L. Formation of three-dimensional supramolecular water architectures containing 1D water chains via dianion templating. *Inorg. Chem. Commun.* **12**, 177–180 (2009).

104. Cui, Y., Cao, M.-L., Yang, L.-F., Niu, Y.-L. & Ye, B.-H. Water nanotubes confined to nanochannels of a (10,3)-*b* net constructed by binary building blocks via the $R^2 2(9)$ synthon. *CrystEngComm* **10**, 1288 (2008).

105. Gu, J.-Z., Lu, W.-G., Jiang, L., Zhou, H.-C. & Lu, T.-B. 3D Porous Metal−Organic Framework Exhibiting Selective Adsorption of Water over Organic Solvents. *Inorg. Chem.* **46**, 5835–5837 (2007).

106. Murugavel, R., Kumar, P., Walawalkar, M. G. & Mathialagan, R. A Double Helix Is the Repeating Unit in a Luminescent Calcium 5-Aminoisophthalate Supramolecular Edifice with Water-Filled Hexagonal Channels. *Inorg. Chem.* **46**, 6828–6830 (2007).

107. Shivaiah, V., Chatterjee, T., Srinivasu, K. & Das, S. K. A Water Pipe Held Up by a Polyoxometalate Supported Transition Metal Complex: Synthesis and Characterization of $[Cu_2(phen)_2(CH_3COO)(CH_3COOH)(H_2O)_2][Al(OH)_6Mo_6O_{18}]\cdot 28H_2O$. *Eur. J. Inorg. Chem.* **2007**, 231–234 (2007).

108. Wang, Y.-T., Tang, G.-M., Liu, Z.-M. & Yi, X.-H. Can One-Dimensional Water Be Controlled by Transformation of Substitution Groups Based on Organic Hosts? *Cryst. Growth Des.* **7**, 2272–2275 (2007).

109. Rodríquez-Llamazares, S. *et al.* The Structure of the First Supramolecular α-Cyclodextrin Complex with an Aliphatic Monofunctional Carboxylic Acid. *European J. Org. Chem.* **2007**, 4298–4300 (2007).

110. Wu, M.-Y. *et al.* A p-Sulfonatothiacalix[4]arene Supramolecular Capsule Containing a Dinuclear Copper(II) Complex. *Supramol. Chem.* **19**, 411–417 (2007).

111. Watanabe, K., Oguni, M., Tadokoro, M. & Nakamura, R. Structural ordering and ice-like glass transition on cooling the nano-channel water formed within a crystalline framework. *J. Phys. Condens. Matter* **18**, 9375–9384 (2006).

112. Tadokoro, M. *et al.* Structures and phase transition of multi-layered water nanotube confined to nanochannels. *Chem. Commun.* **1**, 1274 (2006).

113. Gawronski, J. *et al.* Trianglamines—Readily Prepared, Conformationally Flexible Inclusion-Forming Chiral Hexamines. *Chem. Eur. J.* **12**, 1807–1817 (2006).

114. Tedesco, C. *et al.* Interconnected water channels and isolated hydrophobic cavities in a calixarene-based, nanoporous supramolecular architecture. *CrystEngComm* **7**, 449 (2005).

115. Neogi, S. & Bharadwaj, P. K. An Infinite Water Chain Passes through an Array of Zn(II) Metallocycles Built with a Podand Bearing Terminal Carboxylates. *Inorg. Chem.* **44**, 816–818 (2005).

116. Glidewell, C., Low, J. N., Skakle, J. M. S. & Wardell, J. L. Glyoxal 4-nitrophenylhydrazone: triple helices linked into a three-dimensional channel structure. *Acta Crystallogr. Sect. C Cryst. Struct. Commun.* **61**, o493–o495 (2005).

117. Burrows, A. D., Harrington, R. W., Mahon, M. F. & Teat, S. J. Manipulation of molecular and supramolecular structure in nickel(II) complexes through the orientation of dicarboxylate hydrogen bonding faces. *Cryst. Growth Des.* **4**, 813–822 (2004). ‘

118. Zhao, B. *et al.* Design and Synthesis of 3d-4f Metal-Based Zeolite-type Materials with a 3D Nanotubular Structure Encapsulated ‘Water’ Pipe. *J. Am. Chem. Soc.* **126**, 3012–3013 (2004).

119. Olsher, U., Dalley, N. K., Frolow, F., Shoham, G. & Bartsch, R. A. Evolution of Organized Lariat Ether Alcohol and Diol Hydrate Macrostructures in the Solid State. *J. Incl. Phenom.* **45**, 251–255 (2003).

120. Ahlqvist, M. U. A. & Taylor, L. S. Water dynamics in channel hydrates investigated using H/D exchange. *Int. J. Pharm.* **241**, 253–261 (2002).

121. Stezowski, J. J., Parker, W., Hilgenkamp, S. & Gdaniec, M. Pseudopolymorphism in Tetradeca-2,6-O-methyl-β-cyclodextrin: The Crystal Structures for Two New Hydrates Conformational Variability in the Alkylated β-Cyclodextrin Molecule. *J. Am. Chem. Soc.* **123**, 3919–3926 (2001).

122. Imamura, K. *et al.* Hydrogen-bond network in cyclodecaamylose hydrate at 20 K; Neutron diffraction study of novel structural motifs band-flip and kink in α-(1→4)-D-glucoside oligosaccharides. *Acta Crystallogr. Sect. B Struct. Sci.* **57**, 833–841 (2001).

123. Chui, S. S. Y., Lo, S. M. F., Charmant, J. P. H., Orpen, A. G. & Williams, I. D. A Chemically Functionalizable Nanoporous Material $[Cu_3(TMA)_2(H_2O)_3]_n$. *Science* **283**, 1148–1150 (1999).

124. Aree, T. *et al.* Variation of a Theme: Crystal Structure with Four Octakis(2,3,6-tri-O-methyl)-γ-cyclodextrin Molecules Hydrated Differently by a Total of 19.3 Water. *J. Am. Chem. Soc.* **121**, 3321–3327 (1999).

125. Harata, K., Endo, T., Ueda, H. & Nagai, T. X-Ray Structure of i-Cyclodextrin. *Supramol. Chem.* **9**, 143–150 (1998).

126. Marini, A. *et al.* Dehydration of the cyclodextrins: A model system for the interactions of biomolecules with water. *J. Chem. Phys.* **103**, 7532–7540 (1995).

127. Steiner, T. & Koellner, G. Crystalline β-Cyclodextrin Hydrate at Various Humidities: Fast, Continuous, and Reversible Dehydration Studied by X-ray Diffraction. *J. Am. Chem. Soc.* **116**, 5122–5128 (1994).

128. Betzel, C., Saenger, W., Hingerty, B. E. & Brown, G. M. Topography of cyclodextrin inclusion complexes, part 20. Circular and flip-flop hydrogen bonding in β-cyclodextrin undecahydrate: a neutron diffraction study. *J. Am. Chem. Soc.* **106**, 7545–7557 (1984).

129. Benetollo, F. *et al.* Reaction of lanthanoid chlorides and macrobicyclic polyethers; crystal structure of the hydrated complex between lanthanum chloride and 1,5,12,16,23,26,29,32-octaoxa[$10^{3,14}$][5.5] orthocyclophane. *J. Chem. Soc. Chem. Commun.* 425 (1984).

130. Lindner, K. & Saenger, W. Crystal and molecular structure of cyclohepta-amylose dodecahydrate. *Carbohydr. Res.* **99**, 103–115 (1982).

131. Chacko, K. K. & Saenger, W. Topography of cyclodextrin inclusion complexes. 15. Crystal and molecular structure of the cyclohexaamylose-7.57 water complex, form III. Four- and six-membered circular hydrogen bonds. *J. Am. Chem. Soc.* **103**, 1708–1715 (1981).

132. Okada, Y., Sugai, M. & Chiba, K. Hydrogen-Bonding-Induced Fluorescence: Water-Soluble and Polarity-Independent Solvatochromic Fluorophores. *J. Org. Chem.* **81**, 10922–10929 (2016).

133. Szymkowiak, J. & Kwit, M. Electronic and vibrational exciton coupling in oxidized trianglimines. *Chirality* **30**, 117–130 (2018).

134. Kuhnert, N., Rossignolo, G. M. & Lopez-Periago, A. The synthesis of trianglimines: on the scope and limitations of the [3+3] cyclocondensation reaction between (1*R*,2*R*)-diaminocyclohexane and aromatic dicarboxaldehydes. *Org. Biomol. Chem.* **1**, 1157–1170 (2003).

135. Connolly, M. L. Solvent-Accessible Surfaces of Proteins and Nucleic Acids. *Science* **221**, 709–713 (1983).

136. Macrae, C. F. *et al.* Mercury : visualization and analysis of crystal structures. *J. Appl. Crystallogr.* **39**, 453–457 (2006).

137. Lide, D. R. *CRC Handbook of Chemistry and Physics*. (CRC Press, 2008).

138. Barbour, L. J., Achleitner, K. & Greene, J. R. A system for studying gas-solid reaction kinetics in controlled atmospheres. *Thermochim. Acta* **205**, 171–177 (1992).

139. Brown, M. E. *Introduction to Thermal Analysis*. vol. 3 (Springer Netherlands, 1988).

140. Avrami, M. Kinetics of Phase Change. II Transformation-Time Relations for Random Distribution of Nuclei. *J. Chem. Phys.* **8**, 212–224 (1940).

141. Yanagita, K. *et al.* Kinetics of Water Vapor Adsorption and Desorption in MIL-101 Metal–Organic Frameworks. *J. Phys. Chem. C* **123**, 387–398 (2019).

142. van Heerden, D. P. & Barbour, L. J. Guest-occupiable space in the crystalline solid state: a simple rule-of-thumb for predicting occupancy. *Chem. Soc. Rev.* **50**, 735–749 (2021).

143. Takei, T. *et al.* Changes in density and surface tension of water in silica pores. *Colloid Polym. Sci.* **278**, 475–480 (2000).

144. Etzler, F. M. & Fagundus, D. M. The extent of vicinal water. *J. Colloid Interface Sci.* **115**, 513–519 (1987).

145. Della Gatta, G. Direct determination of adsorption heats. *Thermochim. Acta* **96**, 349–363 (1985).

146. Kim, H. *et al.* Characterization of Adsorption Enthalpy of Novel Water-Stable Zeolites and Metal-Organic Frameworks. *Sci. Rep.* **6**, 19097 (2016).

147. Hummer, G., Rasaiah, J. C. & Noworyta, J. P. Water conduction through the hydrophobic channel of a carbon nanotube. *Nature* **414**, 188–190 (2001).

148. Steiner, T. The Hydrogen Bond in the Solid State. *Angew. Chem. Int. Ed.* **41**, 48–76 (2002).

149. Kalmutzki, M. J., Diercks, C. S. & Yaghi, O. M. Metal-Organic Frameworks for Water Harvesting from Air. *Adv. Mater.* **30**, 1704304 (2018).

150. Knight, A. W., Kalugin, N. G., Coker, E. & Ilgen, A. G. Water properties under nano-scale confinement. *Sci. Rep.* **9**, 8246 (2019).

151. Blagus, A., Cinčić, D., Friščić, T., Kaitner, B. & Stilinović, V. Schiff bases derived from hydroxyaryl aldehydes: molecular and crystal structure, tautomerism, quinoid effect, coordination compounds. *Maced. J. Chem. Chem. Eng.* **29**, 117 (2010).

152. Ogawa, K., Kasahara, Y., Ohtani, Y. & Harada, J. Crystal Structure Change for the Thermochromy of N -Salicylideneanilines. The First Observation by X-ray Diffraction. *J. Am. Chem. Soc.* **120**, 7107–7108 (1998).

153. Ogawa, K. & Fujiwara, T. Tautomerism of a Nitro Derivative of N -Salicylideneaniline in Crystals. *Chem. Lett.* **28**, 657–658 (1999).

154. Ganie, A. A. & Dar, A. A. Water Switched Reversible Thermochromism in Organic Salt of Sulfonated Anil. *Cryst. Growth Des.* **21**, 3014–3023 (2021).

155. Hadjoudis, E. & Mavridis, I. M. Photochromism and thermochromism of Schiff bases in the solid state: structural aspects. *Chem. Soc. Rev.* **33**, 579–588 (2004).

156. Ning, G.-H. *et al.* Salicylideneanilines-Based Covalent Organic Frameworks as Chemoselective Molecular Sieves. *J. Am. Chem. Soc.* **139**, 8897–8904 (2017).

157. Li, X. *et al.* Molecular Engineering of Bandgaps in Covalent Organic Frameworks. *Chem. Mater.* **30**, 5743–5749 (2018).

158. Biswal, B. P. *et al.* Pore surface engineering in porous, chemically stable covalent organic frameworks for water adsorption. *J. Mater. Chem. A* **3**, 23664–23669 (2015).

159. Kandambeth, S. *et al.* Self-templated chemically stable hollow spherical covalent organic framework. *Nat. Commun.* **6**, 6786 (2015).

160. Lesage, A. Recent advances in solid-state NMR spectroscopy of spin I = 1/2 nuclei. *Phys. Chem. Chem. Phys.* **11**, 6876 (2009).

161. Ladizhansky, V., Hodes, G. & Vega, S. Solid State NMR Study of Water Binding on the Surface of CdS Nanoparticles. *J. Phys. Chem. B* **104**, 1939–1943 (2000).

162. Kovacic, J. E. The C=N stretching frequency in the infrared spectra of Schiff's base complexes—I. Copper complexes of salicylidene anilines. *Spectrochim. Acta Part A Mol. Spectrosc.* **23**, 183–187 (1967).

163. Alcañiz-Monge, J., Linares-Solano, A. & Rand, B. Water Adsorption on Activated Carbons: Study of Water Adsorption in Micro- and Mesopores. *J. Phys. Chem. B* **105**, 7998–8006 (2001).

164. Cliffe, M. J. & Goodwin, A. L. PASCal : a principal axis strain calculator for thermal expansion and compressibility determination. *J. Appl. Crystallogr.* **45**, 1321–1329 (2012).

165. Kittaka, S. *et al.* Low temperature phase properties of water confined in mesoporous silica MCM-41: Thermodynamic and neutron scattering study. *J. Chem. Phys.* **138**, 204714 (2013).

166. Johari, G. P. Origin of the enthalpy features of water in 1.8 nm pores of MCM-41 and the large Cp increase at 210 K. *J. Chem. Phys.* **130**, 124518 (2009).

167. Laidler, K. J. Symbolism and terminology in chemical kinetics (Appendix no. V to manual of symbols and terminology for physicochemical quantities and units). *Pure Appl. Chem.* **53**, 753–771 (1981).

168. Engel, T. & Reid, P. *Thermodynamics, statistical thermodynamics, and kinetics*. (Pearson/Prentice Hall, 2006).

169. Watts, T. A., Miehls, E. K. & Swift, J. A. Time-Resolved Cooperative Motions in the Solid-State Dehydration of Thymine Hydrate. *Cryst. Growth Des.* **20**, 7941–7950 (2020).

170. Khutia, A., Rammelberg, H. U., Schmidt, T., Henninger, S. & Janiak, C. Water Sorption Cycle Measurements on Functionalized MIL-101Cr for Heat Transformation Application. *Chem. Mater.* **25**, 790–798 (2013).

171. Magott, M. *et al.* Large breathing effect induced by water sorption in a remarkably stable nonporous cyanide-bridged coordination polymer. *Chem. Sci.* **12**, 9176–9188 (2021).

172. Li, X., Li, Z., Xia, Q. & Xi, H. Effects of pore sizes of porous silica gels on desorption activation energy of water vapour. *Appl. Therm. Eng.* **27**, 869–876 (2007).

173. Wang, J. & Hou, T. Application of molecular dynamics simulations in molecular property prediction II: Diffusion coefficient. *J. Comput. Chem.* **32**, 3505–3519 (2011).

174. Goto, K., Hondoh, T. & Higashi, A. Determination of Diffusion Coefficients of Self-Interstitials in Ice with a New Method of Observing Climb of Dislocations by X-Ray Topography. *Jpn. J. Appl. Phys.* **25**, 351–357 (1986).

175. Koga, K., Gao, G. T., Tanaka, H. & Zeng, X. C. Formation of ordered ice nanotubes inside carbon nanotubes. *Nature* **412**, 802–805 (2001).

176. Devanathan, R., Chase-Woods, D., Shin, Y. & Gotthold, D. W. Molecular Dynamics Simulations Reveal that Water Diffusion between Graphene Oxide Layers is Slow. *Sci. Rep.* **6**, 29484 (2016).

177. Milischuk, A. A. & Ladanyi, B. M. Structure and dynamics of water confined in silica nanopores. *J. Chem. Phys.* **135**, 174709 (2011).

178. Nguyen, H. L. *et al.* A Porous Covalent Organic Framework with Voided Square Grid Topology for Atmospheric Water Harvesting. *J. Am. Chem. Soc.* **142**, 2218–2221 (2020).
